# 19th European Symposium on Radiopharmacy and Radiopharmaceuticals (ESRR’18): Groningen, Netherlands. 05-08 April 2018

**DOI:** 10.1186/s41181-018-0041-4

**Published:** 2018-05-15

## OP01 Novel Acyclic Chelators for ^89^Zr and ^68^Ga

### S. Ait-Mohand^1^, A. Alnahwi^1^, V. Dumulon-Perreault^2^ and B. Guérin^1,2^

#### ^1^Department of nuclear medicine and radiobiology, Faculty of medicine and health sciences, Université de Sherbrooke, Sherbrooke, Québec, Canada J1H 5N4; ^2^Centre d’Imagerie Moléculaire de Sherbrooke (CIMS), CR-CHUS, Sherbrooke, QC, Canada J1H5N4

##### **Correspondence:** B. Guérin

**Aim:** There was continued interest in developing more efficient new chelating agents for metal radionuclides mostly used for positron emission tomography (PET) imaging. We focused on the development of convenient syntheses of acyclic siderophore derivatized with four *N*-hydroxy-*N*-methyl succinamide pendant arms called 4HMS and its bifunctional analog, 4HMSA, for zirconium-89 (^89^Zr) and gallium-68 (^68^Ga) complexation. The aim of this project was to assess the suitability of 4HMS and 4HMSA for ^89^Zr and ^68^Ga-PET.

**Methods & Results:** 4HMS and 4HMSA were prepared through multiple steps starting with a spermine backbone to offer both chelating agents with high overall yield (72-58%). Both chelating agents exhibited strong selective coordination of ^89^Zr and ^68^Ga and offered a very fast labelling kinetic at room temperature as compared to DFO and DOTA/NOTA analogs. Achievable molar activity for ^68^Ga-4HMSA is almost 10 and 3 times higher compared to ^68^Ga-DOTA and NOTA analogs. Molar activity of ^89^Zr-4HMS is approximately 16 fold higher compared to ^89^Zr-DFO. Both radio-complexes *were* stable in saline, serum, as well as against *transchelation* and *transmetallation.* ^68^Ga-4HMSA showed high stability in mouse plasma *in vitro* and *in vivo* over 1h and ^89^Zr-4HMS chelator also showed high stability in mouse plasma over 7 days. Biodistribution and imaging studies were performed in balb/C mice. The background activity in various tissues was low at 1h post-injection (p.i.) for ^68^Ga-4HMSA with a rapid elimination mainly through the kidneys and liver. At the same time point, the activity was largely found in kidneys for ^89^Zr-4HMS chelator. At 24 h p.i. most of the ^89^Zr-4HMS chelator was cleared from all organs and the low amount of activity in kidneys and bone is consistent with the clearance of the intact complex. Finally, the conjugation of unprotected 4HMSA to peptides of biological interest was complete within ~4 h with overall yields of 50-60%.

**Conclusion:** 4HMSA and 4HMS show an outstanding promise as ^68^Ga and ^89^Zr chelators. In terms of Zr^4+^ chelation and stability, 4HMS ligand has proven to be a superior chelator compared to DFO.

## OP02 Improving pretargeting by applying multimerisation on a cyclic chelating scaffold

### D. Summer^1^, S. Mayr^1^, C. Rangger^1^, C. Manzl^2^, C. Decristoforo^1^

#### ^1^Department of Nuclear Medicine, Medical University Innsbruck, Anichstrasse 35, A-6020 Innsbruck, Austria; ^2^Department of General Pathology, Medical University Innsbruck, Müllerstraße 44, A-6020 Innsbruck, Austria

##### **Correspondence:** D. Summer

**Aim:** Pretargeting approaches for antigen targeting combining the excellent target affinity and selectivity of antibodies with the advantages of using short-lived radionuclides such as gallium-68 have attracted increasing interest. Among them especially the bioorthogonal inverse-electron-demand Diels-Alder (IEDDA) reaction between radiolabelled tetrazines (Tz) and trans-cyclooctene (TCO) antibody conjugates has emerged as an effective two-step approach. Here we present an attempt to improve IEDDA pretargeting by preparing multimeric Tz-ligands based on a Fusarinine C (FSC) chelating scaffold for gallium-68 with 3 primary amines for functionalization and evaluating its targeting efficiency.

**Methods:** FSC was partially acetylated, resulting in Monoacetyl- (MAFC) and Diacetyl- FSC (DAFC). Tz functionalization was performed by reaction with NHS-PEG5-Tz with FSC (resulting in the trimer FSC-(PEG5-Tz)3), MAFC (resulting in the dimer MAFC-(PEG5-Tz)2) and DAFC (resulting in the monomer DAFC-(PEG5-Tz), starting from its ferric complex with final iron removal by EDTA, HPLC purification and characterization by MS. ^68^Ga-labelling was performed in acetate buffer (pH 4.5) at high s.a., resulting complexes were characterized by HPLC, protein binding, log P and stability was assessed. Rituximab was functionalized with NHS-TCO according to published procedures (Rtx-TCO). Binding of Tz-conjugates to TCO was assessed on Rtx-TCO immobilized 96well plates as well as after binding of Rtx-TCO vs Rtx to Raji cells expressing CD20 followed by incubation with 68Ga-Tz-conjugate. Biodistribution was studied in normal balb-c mice.

**Results:** Mono- di and trimeric Tz-FSC conjugates were prepared in high yields and could be radiolabelled with gallium-68 at high s.a. revealing good stability in PBS solution and serum, protein binding exceeded 50% for FSC-(PEG5Tz)_3_, MAFC-(PEG5Tz)_2_, DAFC-(PEG5Tz), log p values were below -1. In vitro binding to TCO revealed significantly enhanced binding of the di-and trimeric constructs vs. the monomeric DAFC-(PEG5Tz) both towards isolated Rtx-TCO as well as after binding of Rtx-TCO to CD20 expressing cells (monomer 4.01 ± 0.24%; dimer 7.75 ± 0.56%; trimer 15.9 ± 0.88%). Biodistribution showed a similar profile with rapid renal excretion and moderate uptake in liver and kidneys (<10% ID/g), blood levels increased from the mono to the trimer from 1%-3%ID/g 1h p.i.

**Conclusion:** Our preliminary results show that the preparation of polyvalent Tz-conjugates based on a chelating scaffold is feasible. In vitro results revealed enhanced binding of di- and trimeric constructs vs. the monomer similar to the affects seen in receptor targeting ligands, biodistribution data revealed favourable biodistribution for these pretargeting constructs. Proof of tumour targeting in vivo in respective animal models is currently ongoing.

**Fig. 1 (abstract OP02).**
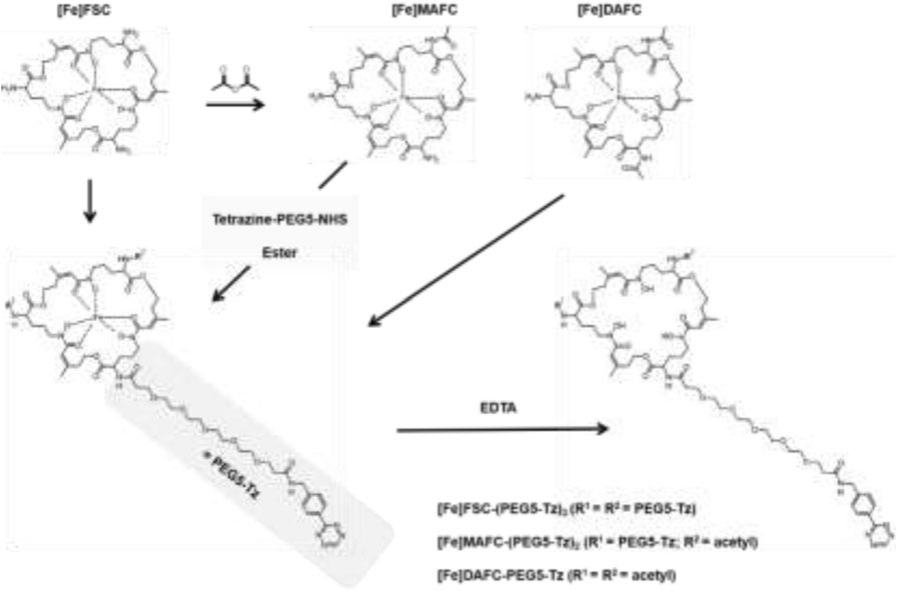
Scheme: Synthetical pathway of tetrazine modified FSC-based pretargeting agents

## OP03 ^99m^Tc-radiolabeling of a poorly soluble protein, a variable heavy chain antibody domain targeting pancreatic β-cells

### M. Ahmadi, S. Bacot, M. Debiossat, C. Ghezzi and P. Perret

#### Univ. Grenoble Alpes, Inserm U1039, LRB, 38000 Grenoble, France

##### **Correspondence:** M. Ahmadi

**Aim:** VH-13 is a single monomeric variable heavy chain antibody domain of a Lama IgG. It will be tested for pancreatic beta cells mass using small animal nuclear imaging. However, VH fragments are known to aggregate in isolation, in the absence of the light-chain partners. The aim of this study was to find the best conditions to radiolabel VH-13 with technetium-99m to avoid the potential formation of aggregates during the radiolabeling process.

**Methods:** VH-13 contains a poly-His tag in C-terminal position that is a specific site suitable for labeling with Tc-99m using the tricarbonyl method. ^99m^Tc-tricarbonyl precursor was prepared before its incubation with VH-13 in different conditions of concentration, temperature and incubation time. Due to the appearance of aggregates during radiolabeling or purification, we first studied different concentrations of several excipients to decrease their formation: 0.2, 10 and 100 mM of arginine (pH=7 or 12), 5% of tween-80 (w/w), 10 and 20% of heparin (w/w), 1 M of acetone or 4% of DMSO (v/v). In a second time, 2 variants of VH-13 were designed with mutations in the VH-VL interface of the protein to improve the solubility. All radiolabeling parameters were then adjusted. All of radiolabeled products were analyzed by radio-HPLC.

**Results:** The presence of arginine at 100 mM allowed to avoid the appearance of aggregates. But whatever the pH conditions, arginine was radiolabeled with Tc-99m. 10 or 20 % of heparin decreased the aggregation, quality control before purification showed a high radiochemical purity (RCP), but more than 80% of the radiolabeled product remained on the purification columns, probably because of a very light aggregation remaining. Adding DMSO was not compatible with the radiolabeling procedure with a RCP < 50%. Finally, the two mutants of VH-13 were radiolabeled and purified successfully. The RCP of radiolabeled products was higher than 95%.

**Conclusion:** Among all the tested excipients, 100 mM of arginine inhibited the aggregation of protein during radiolabeling. Nevertheless, it was also radiolabeled and greatly decreased the labeling yield of VH-13. The synthesis of the two novels mutants including a mutation in the VH-VL interface of protein finally represented the best solution to reduce the aggregation and to allow a successful radiolabeling.

## OP04 Synthesis of PET Radiopharmaceuticals for Cell Radiolabelling Using Anion Exchange Column and Cell Labelling

### A. Socan^1^, P. Kolenc Peitl^1^, M. Krošelj^1^, M. Petrik^2^, C. Decristoforo^3^

#### ^1^Nuclear Medicine Department, University Medical Centre Ljubljana, Ljubljana, Slovenia; ^2^Institute of Molecular and Translational Medicine, Olomouc, Czech Republic; ^3^University Clinic for Nuclear Medicine, University for Medicine, Innsbruck, Austria

##### **Correspondence:** A. Socan

**Aim:** ^111^In labelled radiopharmaceuticals are extensively used for WBC labelling in routine clinical practice. ^64^Cu (T1/2: 12.7h and ^89^Zr (T1/2: 78.4h) are alternative radiometals for synthesis of radiopharmaceuticals used for cell labelling that could enable long-term PET “in vivo” cell tracking with several attractive clinical applications. The aim of study was to prepare ^64^Cu and ^89^Zr tracers (oxine, tropolone) applying synthesis and concentration on an anion exchange column and radiolabel cells (WBCs, RBCs).

**Methods:** Small volumes (1-2 mL) of tracers (oxine, tropolone) in PBS of suitable radiochemical (n-octanol extraction) purity and activity (^64^Cu ≤102 MBq, ^89^Zr ≤3MBq) were prepared on the anion exchange column (SepPAK QMA) using a previously described method. Cells were radiolabelled, WBCs using modified (incubation time 20 min) EANM recommended ^111^In-oxine WBCs labelling method. Labelling efficiency, cell viability, assessed by Trypan Blue exclusion assay and labelling stability (efflux of radioactivity) was determined at different time points after radiolabelling.

**Results:** Synthesis and concentration on SepPAK QMA resulted in ^89^Zr-tropolone with > 79.1% yield, pH 7.0-7.5 and extraction into octanol >94.5%, for ^89^Zr-oxine solution 27.9-70.6% with pH 7-7.9 and extraction > 80%. For ^64^Cu-tropolone >92.5% yield was achieved, pH between 6.9-7.4, extraction into octanol of >89.1%, for ^64^Cu-oxine 55-91.1%, pH 6.9-7.5 and extraction >90.6%. Labelling efficiency of RBCs with ^89^Zr-tropolone was 45-50%, with ^89^Zr-oxine above 64.5%. Labelling efficiency of WBCs was above 81.1% with ^64^Cu-tropolone independent of cell numbers, for ^64^Cu-oxine up to 68% highly dependent on the amount of cells available. Viability of ^64^Cu-tropolone radiolabelled WBCs before and immediately after the labelling was 92% and 85%, respectively 240min after labelling 88% and 81%. Labelling stability of WBCs 240min after cell radiolabelling was above 68% and remained constant up to 48h after labelling. Viability of ^64^Cu-oxine radiolabelled WBCs immediately after the labelling was 71% (90% unlabelled WBCs) and 70% 240min after labelling (85% unlabelled WBCs). Labelling stability of WBCs 240min after cell labelling was above 91% but decreased to 78.1% 48h after labelling. Preliminary μPET studies in animal infection models with ^64^Cu-WBCs showed expected accumulation in infected tissue.

**Conclusion:** The applied on-column synthesis and concentration method enables formation of PET tracers (oxine, tropolone) with good yields, quality and in small volumes suitable for cell radiolabelling. ^89^Zr and ^64^Cu tracers radiolabel cells with sufficient stability and viability, this way making this approach highly promising for routine clinical use.

## OP05 Synthesis of ^18^F-AmBF_3_-losartan and preliminary *in vitro* evaluation as a novel AT_1_R PET radioligand in Oncology

### M. Sahylí Ortega Pijeira^1^, S. Nascimento dos Santos^1^, A. Pérez Nario^1^, Z. Zhang^2^, F. Bénard^2,3,4^, K-S. Lin^2,3,4^, E. Soares Bernardes^1^

#### ^1^Radiopharmacy Center, Nuclear Energy Research Institute, São Paulo, SP 05508-000, Brazil; ^2^Department of Molecular Oncology, BC Cancer Agency, Vancouver, BC V5Z 1L3, Canada; ^3^Department of Functional Imaging, BC Cancer Agency, Vancouver, BC V5Z 4E6, Canada; ^4^Department of Radiology, University of British Columbia, Vancouver, BC V5Z 1M9, Canada

##### **Correspondence:** E. Soares Bernardes

**Aim:** Angiotensin II type 1 receptor (AT_1_R) is a G protein-coupled receptor recognized as a promising cancer therapeutic target. AT_1_R expression has been reported to drive tumor development and progression for several cancers. Losartan, an AT_1_R inhibitor widely used for the treatment of hypertension and congestive heart failure, has been shown to inhibit cancer cell proliferation and angiogenesis. Moreover, losartan derivatives labeled with fluorine-18 (^18^F) and carbon-11 were reported for AT_1_R imaging by positron emission tomography (PET); however, they were mostly employed as AT_1_R PET renal tracers. The present study reports the synthesis of ammoniumethyl-trifluoroborate-losartan (^19/18^F-AmBF_3_-losartan), a new AT_1_R PET radioligand for cancer imaging.

**Methods:** ^19^F-AmBF_3_-losartan was prepared via a copper-catalyzed alkyne-AmBF_3_ and azide-modified losartan cycloaddition at 45 ^o^C for two hours, followed by semi-preparative HPLC purification. Then, ^19^F-AmBF_3_-losartan (25 nmol, 15.3 μg) was radiolabeled with fluoride-18 (555-925 MBq) via an ^18^F–^19^F isotope exchange reaction in aqueous phase at 80 ^o^C for 20 minutes and purified by solid phase extraction using a C18 Light Sep-Pak cartridge. *In vitro* AT_1_R binding studies were performed at 4 ^o^C for one hour using AT_1_R-positive MDA-MB-231 breast cancer cells, AT_1_R-expressing CHO AT_1_R cells, and AT_1_R-negative CHO cells. The *in vitro* studies were conducted in presence or absence of the AT_1_R blocker losartan potassium (100 μM). AT_1_R expression in cells was confirmed by quantitative real-time reverse transcription polymerase chain reaction (RT-PCR).

**Results:** Losartan potassium was converted into tetrazole-protected losartan, azido-modified tetrazole-protected losartan, azide-modified losartan and ^19^F-AmBF_3_-losartan with 83%, 77%, 96% and 52% yields, respectively. Mass spectrometry confirmed their identities. ^18^F-AmBF_3_-losartan was manually prepared in ~35 minutes, with 11 – 18 % radiochemical yield, > 97% radiochemical purity, and 1.8 – 2.9 GBq/μmol specific activity. The identity of ^18^F-AmBF_3_-losartan was confirmed by co-injection with the cold compound on analytical HPLC. *In vitro* studies showed that uptake of ^18^F-AmBF_3_-losartan increased in AT_1_R-expressing MDA-MB-231 and CHO AT_1_R cells in comparison to control AT_1_R-negative CHO cells. Pre-incubation with losartan potassium effectively blocked ^18^F-AmBF_3_-losartan binding to AT_1_R-expressing cells.

**Conclusion:** We developed a facile radiolabeling method to synthesize ^18^F-AmBF_3_-losartan that showed specific binding to AT_1_R-expressing cells *in vitro*. These results demonstrate that ^18^F-AmBF_3_-losartan might be a promising tracer for imaging AT_1_R-expressing tumors.

**References**

1. Liu Y, An S. Ward R, Yang Y, Guo X, Li W, Xu T,[2016], Cancer Lett. 376:226-239

2. Liu Z, Lin K, Bénard F, Pourghiasian M, Kiesewetter DO, Perrin D, Chen X, [2015], Nat. Protoc. 10: 1423-1432

## OP06 Time is Money and Radiation Burden - a carbon-11 ‘two-in-one-pot’ production system

### C. Vraka^1^, C. Philippe^1^, T. Zenz^1^, M. Mitterhauser^1,2^, M. Hacker^1^, W. Wadsak^1,3^, V. Pichler^1^

#### ^1^Medical University of Vienna, Department of Biomedical Imaging and Image-guided Therapy, Vienna, Austria; ^2^Ludwig Boltzmann Institute Applied Diagnostics, Vienna, Austria; ^3^CBmed, Graz, Austria

##### **Correspondence:** C. Philippe

**Aim:** The use of positron emission tomography (PET) for specific molecular examinations is increasing steadily and therefore the demand for selective and specific PET-tracers is rising accordingly. Currently, only one tracer per synthesizer can be produced (non-cassette based) within a time frame of approximately 2 h and a radiation burden of approximately 1 h. A minimum decay time of 6 half-lives (around 2 h) between two carbon-11 productions within the same hot-cell is essential. Therefore, in clinical routine (8 h day) only two syntheses of carbon-11 labeled compounds per day are possible. Consequently, the number of examinations with ^11^C-labeled tracers is extremely limited (number of productions; high synthesis costs and few production runs due to number of hot cells and synthesizers). To improve this situation, the aim of this study was the simultaneous production of two ^11^C-PET-tracers using a ‘two-in-one-pot’ reaction reducing time, cost, and radiation burden. Exemplarily, this simultaneous production was successfully performed for two commonly used brain PET-tracers, [^11^C]Harmine and [^11^C]DASB.

**Methods:** Production runs were performed using a commercially available GE Tracerlab FX C Pro. 1 mg of the precursors, MASB and Harmol, were dissolved in DMSO and 5 M NaOH was added to the solution. [^11^C]CH_3_I was subsequently bubbled through the precursor solution. After a reaction time of 2 min at 100°C, the crude dual-tracer mixture was purified by means of semi-preparative HPLC. The synthesis module was expanded with a self-constructed semi-automated formulation unit (Fig. 1) to ensure parallel SPE-purification and formulation of both tracers after HPLC (Fig. 1).

**Fig. 1 (abstract OP06).**
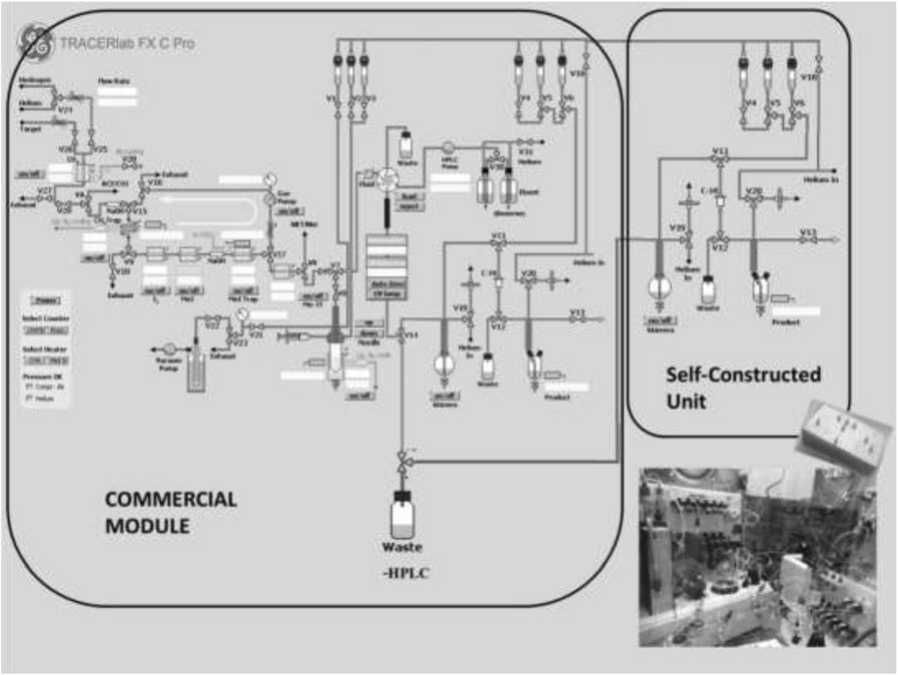
Scheme of the synthesizer including the self-constructed unit for the formulation of the second tracer

**Fig. 2 (abstract OP06).**
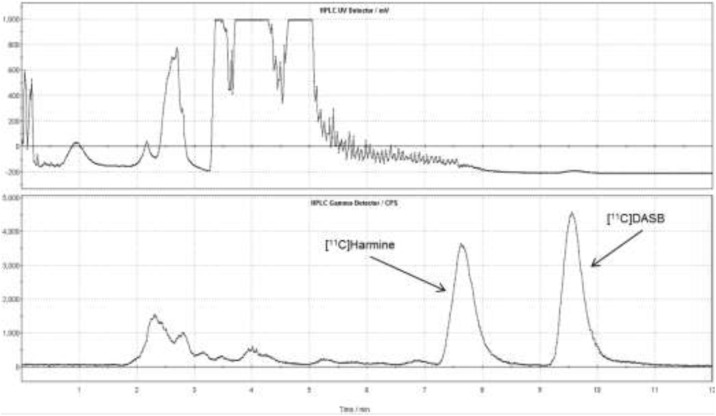
Exemplary RP-HPLC chromatogram for the separation of [^11^C]Harmine and [^11^C]DASB in a single run

**Results:** Both PET-tracers were prepared simultaneously in a ‘two-in-one-pot’ reaction (*n* = 3) and successfully purified using one single HPLC run. Radiochemical yield was 2.0 ± 0.3 GBq (2.3 ±0.5% *not corrected for decay; based on [*^*11*^*C]CO*_*2*_ *@EOB*) for [^11^C]DASB and 2.0 ± 0.7 GBq (2.2 ± 0.8%) for [^11^C]Harmine, respectively. Hence, both products were received in the same amount (ratio 1:1). The qualities of both tracers complied with the European Pharmacopoeia monographs.

**Conclusion:** We herewith describe the first simultaneous production of two ^11^C-PET-tracers in a ‘two-in-one-pot’ reaction. Both products were in full accordance with quality control parameters fulfilling the standards for parenteral human application. This simultaneous radiopharmaceutical preparation lead to a significant reduction of radiation burden, reduction of amount of operator time (-50%), cost reduction (-46.2%) and, subsequently, to considerable gain in overall efficiency of the production process.

## OP07 ^18^F-labelled BODIPY-steroid hormone conjugates as potential bimodal PET and fluorescence receptor imaging agents

### H. ALi^1^, R. Ouellet^1^, A. Pérez Nario^1^, F. Marques^2^, B. Guérin^1^, J. E. van Lier^1^

#### ^1^Department of nuclear medicine and radiobiology, Faculty of medicine and health sciences, Université de Sherbrooke, Sherbrooke, Québec, Canada J1H 5N4; ^2^Centro de Ciências e Tecnologias Nucleares, Instituto Superior Técnico, Universidade de Lisboa, Portugal

##### **Correspondence:** B. Guérin

**Aim:** 4,4-Difluoro-4-bora-3a,4a-diaza-s-indacene (BODIPY) has been used as a fluorescent probe to label a variety of different ligands. BODIPY derivatives have also been radiolabeled with radionuclides for the development of bimodal PET and fluorescent imaging agents. Previously we reported the synthesis of BODIPY-estradiol and androgen conjugates as potential fluorescent probes for receptor imaging in breast and prostate cancers.^1^ Relative binding affinities of a series of BODIPY-estradiol conjugates revealed that only the analog featuring an eight carbon spacer between the two entities showed good receptor binding affinity.^2^ We recently confirmed by *in vitro* fluorescence imaging that this analog localizes through a receptor-mediated process on cancer cells that over-express the estrogen receptor.^3^ As a continuation of these studies, we evaluated various routes to prepare the analogous bimodal ^18^F-labelled BODIPY-steroid conjugates.

**Method & Results:** ^18^F-labeled 4-iodophenyl substituted BODIPY was first prepared by the SnCl_4_-assisted ^18^F-^19^F isotopic exchange method (50-70%).^4^ This radiolabeled moiety was subsequently conjugated in high yield to the C17α-position of estradiol and androgen derivatives under Sonogashira cross coupling reaction conditions using Pd-catalyst, base and CuI. Direct labelling of the conjugates using SnCl_4_-assisted ^18^F-^19^F isotopic exchange method resulted in the simultaneous addition of a Cl-atom to the C17α-ethynyl group and a poor molar activity.

**Conclusion:** Our studies confirm the potential to prepare 18F-labelled analogs of BODIPY-steroid conjugates. We are currently evaluating an alternative approach using 4-dimethylaminopyridine BODIPY as precursor to allow the efficient incorporation of 18F to potentially generating higher molar activity conjugates suitable for receptor-based bimodal imaging studies.

**References**

1. Osati S, Ali H, Guérin B, van Lier JE [2017] Steroids 123: 27–36

2. Osati S, Ali H, Marques F, Paquette M, Beaudoin S, Guérin B, Leyton JV, van Lier JE [2017] Bioorg Med Chem Lett 27: 443-446

3. Marques F, et al. [2017] unpublished data

4. Liu S, Li D, Zhang Z, Prakash GKS, Conti PS, Li Z [2014] Chem Commun 50: 7371

## OP08 Copper-mediated radiofluorination of aryl pinacol boronates in the presence of pyridinium sulfonates

### D. Antuganov^1^, M. Zykov^1^, K. Timofeeva^1^, V. Timofeev^1^, V. Orlovskaya^2^, R. Krasikova^2^

#### ^1^National Almazov Medical Research Centre, Saint-Petersburg, Russian Federation; ^2^N.P. Bechtereva Institute of Human Brain, Russian Academy of Science, Saint-Petersburg, Russian Federation

##### **Correspondence:** D. Antuganov

**Aim:** Nowadays copper-mediated radiofluorination of arylboronic acids pinacol esters (ArylBPin) found ample application for ^18^F-labeling of various electron-rich arenes.^1^ The improvement of this methodology, in particular, ^18^F-recovery step, has been in focus of the recent researches.^2,3^ The suggested use of aqueous solutions of non-ionic bases for ^18^F-elution was efficient, however in non-aqueous solutions these weak bases cannot reach sufficient degree of protonation for ^18^F-recovery. Non-aqueous solutions of 4-dimethylaminopyridinium sulfonates may constitute an amenable alternative. These salts may serve both as a source of co-ligand for Cu(OTf)_2_py_4_ catalyst, increasing its efficiency, ^4^ and as appropriate PTC agents.

**Methods:** Following earlier suggested procedure^2^ an aqueous [^18^F]fluoride was loaded onto an anion exchange cartridge from the male side (OASIS WAX 1cc, 30 mg, preconditioned with 0.5 M NaHCO_3_ and water). The cartridge was rinsed with 1 ml of *i-*PrOH in the same direction and dried by compressed air. [^18^F]Fluoride was eluted from female side with 25 μmol of 4-dimethylaminopyridinium tosylate (4-DMPTs) or 4-dimethylaminopyridinium triflate (4-DMPTf) in 0.5 mL of DMA into the vial containing 5.3 μmol of Cu(OTf)_2_py_4_ and 17.9 μmol of ArylBPin precursor in 0.5 ml of DMA. The mixture was heated (110 °C, 20 min) in a sealed vial under air. After cooling radiochemical conversion (RCC) was determined by radioTLC.

**Results:** Both sulfonates provided similar ^18^F-elution efficiency (EE), however higher RCC were achieved using triflate salt. Notably, with this protocol relatively small amount of precursor and catalyst was required. Its feasibility was confirmed for a series of model aromatic substrates.

**Conclusion:** Easily accessible 4-dimethylaminopyridinium triflate has shown to be suitable ^18^F-eluting agent for alcohol-enhanced Cu-mediated radiofluorination. The developed procedure is very simple, avoids any solvents evaporation steps and easy adaptable to automation. Work is now in progress to adapt this procedure to the preparation of clinically relevant radiotracers. Research support: RFBR grant № 16-54-12062\16.

**References**

1. Tredwell A et al., [2014], Angew. Chem. Int. Ed. 53: 7751-77552. Zlatopolskiy BD et al., [2015], Chem. Eur. J. 21: 5972-59793. Mossine AV et al., [2017], Sci. Rep. 7: 233-24

4. Antuganov D et al., [2017], ChemSelect, 2: 7909-7912

**Table 1 (abstract OP08).** See text for description

№	Precursor	Salt	EE, %	RCC,% (*n* = 3)
1	4-Biphenylboronic acid pinacol ester	4-DMPTs	70±1	65±5
2	4-Biphenylboronic acid pinacol ester	4-DMPTf	78±1	96±3
3	3,4-Dimethoxyphenylboronic acid pinacol ester	4-DMPTf		94±2
4	2-Methoxyphenylboronic acid pinacol ester	4-DMPTf		83±4
5	4-Methoxyphenylboronic acid pinacol ester	4-DMPTf		89±5

## OP09 Development of biocompatible and functionalised polymer nanoparticles for the specific vectorisation of an imaging agent

### N. Lepareur^1,2^, E. Vène^2^, C. Bouvry^1,3^, S. Cammas-Marion^2,4^, P. Loyer^2^

#### ^1^Centre Eugene Marquis, Radiopharmacy, Rennes, France; ^2^Institut NuMeCan UMR 1241 Inserm/INRA/Rennes University, Rennes; ^3^Rennes University, ISCR CNRS UMR 6226, 35708, Rennes, France; ^4^ENSCR, ISCR CNRS UMR 6226, Rennes, France

##### **Correspondence:** N. Lepareur

**Aim:** Nanomedicine, the application of nanotechnologies to the medical domain, is a fast-growing research field, especially the production of nanoparticles enabling encapsulation then controlled and targeted release of molecules of interest, such as a cytotoxic drug. Diagnostic could also benefit from the use of such nanovectors containing one or more imaging agent(s) (fluorescent dye, contrast agent, radionuclide) to image a tumour.

**Methods:** Benzyl polymalate and its derivatives (pegylated and biotinylated) were prepared according to the method previously described. ^(1)^ Different techniques have been used to formulate the nanoparticules and to incorporate an imaging agent (DiD-Oil, fluorescein amine and a ^99m^Tc-based radiotracer). The resulting nanoparticles were characterised using various techniques (DLS, zetametry, AF4, TEM, EDS). Preliminary in vitro studies have also been done.

**Results:** Prepared nanoparticles were monodisperse and stable. To encapsulate fluorescent DiD-Oil and lipophilic ^99m^Tc-SSS radiotracer, nanoprecipitation method was not suitable and had to be modified. ^(2)^ The resulting nanoparticles were also monodisperse and stable, with a slightly higher diameter. Electron-dispersive spectroscopy coupled to TEM demonstrated the presence of sulphur in the nanoparticles, thus confirming the encapsulation of the radiotracer. Various peptides have been grafted through streptavidin and their affinity to different hepatoma cell lines was tested.

**Conclusion:** We have developed a family of nanoparticles based on degradable, biocompatible and functionalisable polymers, enabling the binding of specific targeting agents (eg. peptides) and incorporating an imaging agent, either for optical or scintigraphic imaging. These objects deserve further investigation to gain a deeper understanding on their properties and in vivo behaviour. Direct grafting of the most promising peptides is underway. Encapsulation of a therapeutic radiotracer is also planned.

**References**

1. Huang ZW Laurent V, Chetouani G, Ljubimova JY, Holler E, Benvegnu T, Loyer P, Cammas-Marion S, [2012], *Int J Pharm* 423: 84-92.

2. Lepareur N, Leal E Costa L, Bocqué M, Blondelle C, Ruello C, Desjulets M, Noiret N, Cammas-Marion S, [2015], Front Med (Lausanne) 2:63

## OP10 Development of a new generation propylene cross-bridged chelator as versatile platform for antibody radiolabeling with Cu-64

### S. Sarkar^1^, R.Pal^1^, Y. Su Ha^1^, P. Tu Huynh^1^, W. Lee^1^, N. Soni^1^, J-M Jung^1^, J.Y. Kim^2^, K. Chul Lee,^2^ J. Yoo^1^

#### ^1^Kyungpook National University, Department of Molecular Medicine, BK21 Plus KNU Biomedical Convergence Program, Daegu, South Korea; ^2^Department of RI-Convergence Research, Korea Institute of Radiological and Medical Sciences, Seoul, South Korea

##### **Correspondence:** S. Sarkar

**Aim:** Monoclonal antibodies have been widely exploited for both diagnostic and therapeutic purposes. For antibody radiolabeling with Cu-64, a bifunctional chelator is required which can seize the radio metal quite robustly into its cavity. Thus development of cross-bridged chelator is in need. Unlike the other biomolecules (e.g., peptide, aptamer), antibody radiolabeling condition is tricky and needs sophisticated and mild labelling conditions which limits the application of cross-bridged chelator in immuoPET. We report a new kind of cross-bridged macrocyclic chelator (PCB-TE2A-alkyne), which can be converted to any copper free clickable moiety or any functional group for antibody conjugation (Fig. 1). It thus enables a general platform for antibody tagging with Cu-64.

**Methods:** The propylene cross-bridged chelator was modified to different clickable moieties and various functional groups in a single step prior to radiolabeling. To confirm its applicability radiolabeled chelator was then conjugated to trastuzumab and biodistribution and microPET imaging was done in NIH3T6.7 tumor-bearing BALB/c nude mice.

**Results:** Starting from cyclam the PCB-TE2A-alkyne was synthesized in five consecutive steps with 32% overall yield. It was then modified to different functional groups, viz., tetrazine, NHS-ester, maleimide, in a single step in excellent yield. Modifications to different functional groups did not require any HPLC purification. In further, the tetrazine modified chelator was radiolabeled with Cu-64 in high yield and conjugated to trastuzumab within 10 minutes at room temperature.

**Fig. 1 (abstract OP10).**
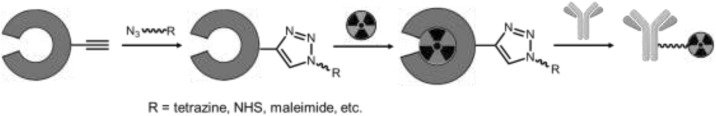
See text for description

**Conclusion:** A versatile propylene cross-bridged macrocyclic chelator for radiolabeling of heat-sensitive antibody with Cu-64 was demonstrated.

**Acknowledgements**

This work was supported by NRF (2016R1A2B4011546, 2013R1A4A1069507, 2017M2C2A1014006, 2017M2A2A6A02018506, 2017R1D1A1B03033974, HI17C0221, & KRF 7072016H1D3A1907667) and BK21 Plus KNU Biomedical Convergence Program, Korea.

## OP11 *Pseudomonas aeruginosa* infection imaging with Ga-68 labelled pyoverdine

### M. Petrik^1^, E. Umlaufova^1^, V. Raclavsky^2^, A. Palyzova^3^, V.Havlicek^3,4^, Z. Novy^1^, C. Decristoforo^5^, M. Hajduch^1^

#### ^1^Institute of Molecular and Translational Medicine, Faculty of Medicine and Dentistry, Palacky University, Olomouc, Czech Republic; ^2^Department of Microbiology, Faculty of Medicine and Dentistry, Palacky University, Olomouc, Czech Republic; ^3^Institute of Microbiology of the Czech Academy of Sciences, v.v.i., Prague, Czech Republic; ^4^Regional Centre of Advanced Technologies and Materials, Department of Analytical Chemistry, Faculty of Science, Palacky University, Olomouc, Czech Republic; ^5^Clinical Department of Nuclear Medicine, Medical University Innsbruck, Innsbruck, Austria

##### **Correspondence:** M. Petrik

**Aim:** *Pseudomonas aeruginosa* (*P.a.*) is an increasingly prevalent opportunistic pathogen that causes a variety of life-threatening nosocomial infections, especially in immunocompromised hosts. The diagnosis of infections caused by *P.a.* can be challenging due to inconvenient diagnostic methods, which are often slow, invasive or lack sensitivity and/or specificity. Novel diagnostic tools are urgently needed. One of the novel diagnostic strategies for the specific detection of *P.a.* infections could be utilization of radiolabelled siderophores. Siderophores are small molecules produced by many microorganisms to scavenge essential iron. Replacing iron in siderophores by suitable radiometal could open approaches for targeted imaging of infection. Here we report on the preclinical evaluation of Ga-68 labelled pyoverdine, siderophore produced by *P.a.*, for specific diagnosis of *P.a.* infections.

**Methods:** Radiolabelling of pyoverdine isolated from *P.a.* with Ga-68 was performed using acetate buffer. Radiochemical purity was analyzed by RP-HPLC and ITLC-SG. Protein binding, partition coefficient and stability values of ^68^Ga-pyoverdine in human serum and in the excess of competing chelator and metal were determined. *In vitro* uptake was tested in various microbial cultures. *Ex vivo* biodistribution was studied in normal Balb/c mice. Uptake of ^68^Ga-pyoverdine by *P.a. in vivo* was studied in respiratory and muscle infection animal models using PET/CT imaging. *In vivo* specificity of ^68^Ga-pyoverdine for *P.a.* was compared with other radiopharmaceuticals.

**Results:** Pyoverdine was labelled with ^68^Ga with high (>95%) radiochemical purity. The resulting complex showed hydrophilic properties (log P = -3.07±0.08), low protein binding (<3% up to 120 min incubation) and ~95% stability in human serum. *In vitro* uptake of ^68^Ga-pyoverdine was highly dependent on iron load and type of microbial culture. In *P.a.* cultures high uptake under iron-deficient conditions was observed that could be blocked. Furthermore in all other tested microbial cultures the uptake of ^68^Ga-pyoverdine was significantly lower. In normal mice ^68^Ga-siderophore showed rapid renal excretion and low blood values (0.09±0.01 %ID/g) even at a short time period (90 min) after application. PET/CT imaging in infected animals displayed specific accumulation of ^68^Ga-pyoverdine in infected tissues and better distribution than other, clinically used radiopharmaceuticals.

**Conclusion:** We have shown that pyoverdine can be labelled with Ga-68 with high affinity and radiochemical purity. ^68^Ga-poverdine displayed suitable *in vitro* characteristics and excellent pharmacokinetics. The high and specific uptake of ^68^Ga-pyoverdine by *P.a.* was confirmed both *in vitro* and *in vivo*, proving its potential for specific imaging of *Pseudomonas* infections.

**Acknowledgement**

We gratefully acknowledge the financial support of Technology Agency of the Czech Republic (Project No. TE01020028).

## OP12 Modifying the siderophore triacetylfusarinine C for molecular imaging applications

### P. Kaeopookum^1,4^, D. Summer^1^, L. Kochinke^1^, T. Orasch^2^, B. Lechner^2^, M. Petrik^3^, Z. Novy^3^, C. Rangger^1^, H. Haas^2^, C. Decristoforo^1^

#### ^1^Department of Nuclear Medicine, Medical University Innsbruck, Innsbruck, Austria; ^2^Division of Molecular Biology, Biocenter, Medical University Innsbruck, Innsbruck, Austria; ^3^Faculty of Medicine and Dentistry, Institute of Molecular and Translation Medicine, Palacky University, Olomouc, Czech Republic; ^4^Research and Development Division, Thailand Institute of Nuclear Technology, Nakhonnayok, Thailand

##### **Correspondence:** P. Kaeopookum

**Aim:** Invasive pulmonary aspergillosis (IPA) mainly caused by *Aspergillus fumigatus* (*AFU*) is a major cause of mortality in immunosuppressed patients mainly due to the lack of sensitive and specific diagnostic procedures. *AFU* secrets the siderophore triacetylfusarinine C (TAFC) to sequester iron for acquisition and uptake via the MirB transporter. This system is essential for *AFU* virulence and is highly upregulated during infection. We have shown that TAFC can be radiolabeled with ^68^Ga thereby exhibiting excellent targeting properties in an *AFU* infection model [1]. Here we aimed to modify TAFC and investigate the influence of introduced substituents on preservation of *AFU*-targeting characteristics *in vitro* and *in vivo* by μPET/CT imaging.

**Methods:** TAFC derivatives with various substituents (different carbon chain lengths, charges, fluorescent dye) were synthesized starting from the deacetylated forms of TAFC, characterized by HPLC and MS and radiolabeled with ^68^Ga. Stability, protein binding and logP values were determined. *In vitro* uptake by *AFU* was performed in iron-depleted and iron-replete cultures. Selected compounds with highest, lowest and comparable uptake ratio to TAFC were studied regarding their biodistribution behaviors in normal BALB/c mice as well as via μPET/CT imaging in healthy and *AFU*-infected Lewis rats.

**Results:** 15 different TAFC derivatives with varying substitutions were synthetized in high yields and could be labeled with ^68^Ga at high specific activity. Lipophilicities as expressed in logP were -0.38 to -3.80 ([^68^Ga]TAFC -2.1)*. In vitro* uptake studies revealed retained recognition by the MirB transporter with reduced uptake efficiency with increasing number of substitutions (mono-, >di, > tri). Introduction of fluorescent dye (FITC) allowed imaging of uptake and processing of TAFC analogs. Three selected compounds, [^68^Ga]DABuFC, [^68^Ga]TPFC and [^68^Ga]FSC(suc)_3_, displayed low protein binding and were stable in PBS and serum. Biodistribution behavior and image contrast by μPET/CT of [^68^Ga]DABuFC was comparable to [^68^Ga]TAFC whereas [^68^Ga]TPFC showed higher uptake in intestine. The derivative with the lowest *in vitro* uptake, [^68^Ga]FSC(suc)_3_, displayed no signal in μPET/CT image of infected Lewis rats.

**Conclusion:** This study shows the possibility of TAFC modification without losing its *in vitro* and *in vivo* properties to target *AFU* via specific recognition of the MirB transporter. Substitution of one acetyl group of TAFC by functionalities such as fluorescent dyes opens alternative strategies for theranostics of infectious diseases.

**Reference**

1. Petrik M, Haas H, Dobrozemsky G, et al. [2010], J Nucl Med 51(4): 639-645.

## OP13 *In vitro* and *in vivo* comparison of the novel ^89^Zr chelator DFO-cyclo* with DFO

### R. Raavé^1^, G. Sandker^1^, S. Heskamp^1^, O. Boerman^1^, M. Rijpkema^1^, F. Mangin^2^, M. Meyer^2^, J-C. Chambron^2^, M. Moreau^2^, C. Bernhard^2^, V. Goncalves^2^, F. Denat^2^

#### ^1^Department of Radiology and Nuclear Medicine, Radboud Institute of Molecular Life Sciences, Radboud university medical center, Geert Grooteplein-Zuid 10, Nijmegen, the Netherlands; ^2^Institut de Chimie Moléculaire de l'Université de Bourgogne (ICMUB), UMR CNRS 6302, Université de Bourgogne–Franche-Comté, 9 avenue A. Savary, BP 47870, 21078 DIJON Cedex, France

##### **Correspondence:** R. Raavé

**Aim:** The current “gold standard” chelator to label antibodies with ^89^Zr for immunoPET is desferrioxamine (DFO). Preclinical studies have shown that the ^89^Zr-DFO complex is partly unstable *in vivo*, resulting in release of ^89^Zr and subsequent accumulation in mineral bone tissue. This bone uptake may prevent the detection of bone metastases, and hampers accurate estimation of the radiation dose to the bone marrow in dose planning for radioimmunotherapy. Therefore, there is a need for a more stable ^89^Zr chelator. Here we report DFO-cyclo*, a preorganized extended DFO derivative introducing an octacoordination, and investigate the stability of its ^89^Zr complex over the unsaturated hexacoordinated ^89^Zr-DFO complex *in vitro* and *in vivo*.

**Methods:** DFO-cyclo* was prepared by coupling of a cyclic hydroxamate group to DFO. Trastuzumab was conjugated with DFO-cyclo*-*p*Phe-NCS or DFO-*p*Phe-NCS and radiolabeled with ^89^Zr. Stability of the labeled antibody conjugates was evaluated in human plasma and in PBS with a 1000-fold molar excess of EDTA or DFO at 37°C up to 7 days. The immunoreactive fraction, IC_50_ and internalization capacity of ^89^Zr-DFO-cyclo*-trastuzumab or ^89^Zr-DFO-trastuzumab were evaluated *in vitro* using HER2-expressing SK-OV-3 cells. The *in vivo* distribution of ^89^Zr-DFO-cyclo*-trastuzumab and ^89^Zr-DFO-trastuzumab was investigated in mice with subcutaneous SK-OV-3 xenografts by *ex vivo* tissue analyses and PET/CT imaging.

**Results:** Labeling efficiencies exceeded 99% and specific activities > 150 MBq/mg were reached for both ^89^Zr-DFO-cyclo*-trastuzumab and ^89^Zr-DFO-trastuzumab. When challenged with an excess of EDTA or DFO at 37°C for 7 days, ^89^Zr-DFO-cyclo*-trastuzumab showed significantly higher stability than ^89^Zr-DFO- trastuzumab: 99 ± 1% vs. 61 ± 1%, and 55 ± 3% vs. 44 ± 3%, respectively. Immunoreactive fractions of 65% and 61% and IC_50_ values of 2.89 nM and 2.93 nM were found for ^89^Zr-DFO-cyclo*-trastuzumab and ^89^Zr-DFO-trastuzumab, respectively. Internalization after 2 h was significantly higher for ^89^Zr-DFO-cyclo*-trastuzumab (26.6 ± 1.1%) compared to ^89^Zr-DFO-trastuzumab (22.4 ± 1.5%) (*p* < 0.005). Bone uptake (%ID/g) was significantly lower for ^89^Zr-DFO-cyclo*-trastuzumab compared to ^89^Zr-DFO-trastuzumab in knee (3.6 ± 0.4% vs. 5.9 ±0.6%), femur (2.2 ± 0.2% vs. 3.4 ± 0.3%), and sternum (3.5 ± 0.4% vs. 4.5 ± 0.4%) at 72 h after injection (*p* < 0.005). Tumor uptake and blood clearance did not differ significantly.

**Conclusion:** ^89^Zr-DFO-cyclo*-trastuzumab shows improved *in vitro* and *in vivo* stability compared to ^89^Zr-DFO-trastuzumab. In immunoPET, less radiation exposure to bone marrow, improved bone metastasis detection and improved radioimmunotherapy dose planning may be achieved using DFO-cyclo*.

## OP14 Characterization by Radio_HPLC of Cell Effluxes and Cell Extracts of ^99m^Tc-HMPAO Human Leukocytes

### E. Fernandez Muñoz, M.A. Asensio Ruiz, A. Abella Tarazona, T. Martínez Martínez

#### Unidad de Radiofarmacia. Hospital Virgen de la Arrixaca, Murcia, Spain

##### **Correspondence:** T. Martínez Martínez

**Aim:** The labelling of autologous WBCs is a standard clinical practice for the scintigraphic detection of infectious or inflammatory disease. The mechanism of cell labeling is based on the lipophilic complex formed upon reconstitution, which can freely cross the cell membrane and once in the cytoplasm, is transformed into a secondary complex. However, some efflux from cell has been reported, causing errors of interpretation in the scintigraphic studies, since the activity detected does not always correspond with true cellular uptake. In this sense, it is postulated by some authors that the conversion to secondary complex is reversible, and over the time, a percentage of the primary complex tends to appear and elute from cells.

**Methods:** Leukocytes (≈10^8^) from healthy human volunteers (*n* = 5) were labelled following guideline, splitted into two aliquots of 0.5 cc in plasma and saline and incubated under stirring at 37°C, for 2h and 4h. After incubation, samples were centrifuged at 2000xg and the supernatant was analyzed by HPLC in a C18 (4 μm, 3.9x150 mm) column with a gradient mobile phase of 0.05M sodium acetate/tetrahydrofuran at a flow rate of 1.5 ml/min. The percentages of primary and secondary complex and free ^99m^Tc pertechnetate were determined by their retention time (tr). ^99m^Tc-HMPAO-human leukocytes (*n* = 3) were mechanically lysed and extracted in 0.5 ml of water for injection, centrifuged at 2000xg and analyzed as described.

**Results:** Percentages of 17.8±0.7 of free ^99m^ Tc pertechnectate (tr = 1,5 min) and 82.1±0.7 % of secondary complex (tr = 4,5 min) were found in saline at 2 and 4 h. Results in plasma were of 21.7±2.1 % for ^99m^Tc free pertechnectate and 78.3 ± 2.1% for secondary complex. No primary complex was detected (tr = 8 minutes) at any time of incubation. Cell extracts showed percentages of 8.1±1.1 % of free ^99m^ Tc pertechnectate and 92.1±1.2% of secondary complex. No primary complex was detected.

**Conclusion:** While secondary complex was detected inside and outside the cells, primary complex was absent in extracts and effluxes at any time of study. The percentage of 99m Tc pertechnetate is probably due to oxidation during the incubation period. According to our results, the activity detected in cell effluxes is related to the exit of secondary complex, rather than the exit of primary complex after reversibility of the original conversion inside the cell.

**References**

1. De Vries EF, Roca M, Jamar F, Israel O, Signore A. 2010. Eur J Nucl Med Mol Imaging. 37(4):842-8.

2. Neirinckx RD, Burke JF, Harrinson RC, Forster AM, Andersen AR, Lassen NA. 1988. J Cereb Blood Flow Metab. Vol 8(Suppl 1): 1-11.

3. Kao CH, Wang YL and Wang SJ. 1992. J Nucl Med Technol; 20(4):224-7

4. Hung JC, Corlija M, Volkert WA. 1988. J Nucl Med.29(9):1568-76

## OP15 *In vivo* imaging of mGluR1 neuroreceptor kinetics in mouse brain with [^11^C]ITDM microPET

### Š. Korat^1^, D. Bertoglio^1^, J. Verhaeghe^1^, I. Munoz-Sanjuan^2^, C. Dominguez ^2^, L. Liu^2^, L. Mrzljak^2^, S. Staelens^1^, L. Wyffels^1^

#### ^1^Molecular Imaging Center Antwerp, University of Antwerp, Antwerp, Belgium; ^2^CHDI Foundation, Princeton, New Jersey, USA

##### **Correspondence:** Š. Korat

**Aim:** Glutamate, the predominant central nervous system excitatory neurotransmitter, binds to Group 1 metabotropic glutamate receptors: mGluR1 and mGluR5. mGluR1 levels are the highest in extrastriatal areas, such as thalamus and cerebellum. Therefore, mGluR1 PET ligands could present a potential biomarker for glutamateric system in these extrastriatal areas involved in the pathophysiology of Huntington’s disease.

**Methods:** We validated [^11^C]ITDM as a PET probe for imaging the kinetics of mGluR1 in mouse brain. [^11^C]ITDM was prepared by modification of a previously described method^1^ in a module (Comecer Netherlands) adapted for automated production. In a cooled solution containing the arylstannane precursor (1.9mg), K_2_CO_3_ (2.1mg), CuCl_2_ (1.5mg), Pd_2_(dba)_3_ (1.3mg) and P(o-tol)_3_ (1.7mg) in DMF purged with N_2_, [^11^C]MeI was bubbled, followed by reaction at 65°C for 5min. The mixture was purified by reverse phase semi-preparative HPLC (t_R_=8.5min), using MeCN:H_2_O:Et_3_N (6:4:0.01, *V/V/V*) as mobile phase (2.7mL/min) followed by sterile formulation into ethanolic saline containing 0.5% ascorbic acid, using a C18 Sep-Pak cartridge. For *in vivo* evaluation, WT Q175DN mice (*n* = 9; 11 months old) were intravenously (i.v.) injected with [^11^C]ITDM (5.69±2.4MBq; injected mass=0.87±0.33μg/kg) and dynamically scanned for 90min (Siemens Inveon μPET/CT). To validate target engagement, blocking and displacement studies with mGluR1 antagonist YM-202074 (20mg/kg; i.v.; 2min before and 30min after tracer injection, respectively) were performed. Total volume of distribution (V_T_) (from 2TCM) was calculated noninvasively using an image-derived input function (IDIF) in several brain regions.

**Results:** [^11^C]ITDM was successfully implemented with a synthesis time of 34min from EOB (end of bombardment) including formulation, molar activity (A_M_) of 122.4±22.2GBq/μmol at EOS (end of synthesis), radiochemical purity of >99% and decay corrected radiochemical yield of 3.7±1.5% (based upon [^11^C]CO_2_). V_T_ quantification of [^11^C]ITDM PET imaging confirmed tracer uptake primarily in cerebellum (9.1±2.1mL/cm^3^) and thalamus (8.2±2.1mL/cm^3^). Furthermore, YM-202074 administration showed significant blockade and displacement of [^11^C]ITDM in all investigated brain regions, confirming target engagement of mGluR1.

**Conclusion:** [^11^C]ITDM was successfully implemented in high purity and good A_M_. Our initial *in vivo* evaluation confirmed [^11^C]ITDM selective binding to mGluR1 in all brain regions, having repercussions for reference region selection.

**Reference**

1. Fujinaga M et al, [2012], JMC 55: 11042-11051

## OP16 Anesthesia affects P-glycoprotein function at the Blood-Brain Barrier: A PET study with [^18^F] MC225 in rats

### L. García Varela, D. Vállez García, A. Van Waarde, J.W.A. Sijbesma, R.A.J.O. Dierckx, A. Schildt, C. Kwizera, P.H. Elsinga and G. Luurtsema

#### University of Groningen, University Medical Center Groningen, Department of Nuclear Medicine and Molecular Imaging, Hanzeplein 1, 9713 GZ Groningen, The Netherlands

##### **Correspondence:** L. García Varela

**Aim:** P-glycoprotein transporters (P-gp) at the Blood-Brain barrier (BBB) are efflux pumps that play an important role in protecting the brain against harmful substances. The expression and function of these proteins is of great interest in neurodegenerative diseases and drug resistance. Its function can be measured in vivo with positron emission tomography (PET) using the novel tracer 5-(1-(2-[18F]fluoroethoxy))-[3-(6,7-dimethoxy-3,4-dihydro-1H-isoquinolin-2-yl)-propyl]-5,6,7,8-tetrahydronaphthalen, [^18^F]MC225. Preclinical PET scans are performed under anesthesia, and in most longitudinal studies the animals are repeatedly anesthetized. However, the potential effect of anesthesia on P-gp function has not been explored yet using this tracer. Therefore, the aim of this study is to assess the effect of a pre-exposure to anesthesia on P-gp function in rats with [^18^F]MC225.

**Methods:** Six rats were anesthetized with isoflurane for 90 minutes. Five other rats were not subjected to anesthesia. One week later, all rats underwent a dynamic PET scan (60 min) with arterial blood sampling. [18F]MC225 with a dose of 32±6 MBq and a molar activity higher than 29000 GBq/mmol was injected into a tail vein as a bolus of 1ml/min. After tracer injection, blood samples (0.15 ml) were collected to measure radioactivity in whole blood and plasma, besides the parent fraction of the tracer and its radioactive metabolites. The images were processed with PMOD software, and registered to a tracer-specific brain template. A total of 16 regions inside the brain were selected from a Wistar brain rat atlas. The volume of distribution (VT) which reflects P-gp function was calculated by Logan analysis using plasma activity corrected for metabolites as input function.

**Results** Significant differences in V_T_ of the whole brain were observed (p=0.002) between the 2 groups. In control animals, V_T_ of the whole brain was 11.04±1.25, whereas in animals pretreated with anesthesia it was 7.89±1.25, a decrease of 29%. Moreover, significant decreases in V_T_ were found between groups in all the brain regions analyzed such as amygdala, hippocampus, hypothalamus, midbrain. Tracer concentration in whole-blood and plasma, and the rate of metabolism were not significantly different between the groups.

**Conclusion:** Our results suggest that anesthesia has a prolonged effect on P-gp function at the BBB, lasting at least one week. The group with additional anesthesia displayed a significant decrease of tracer uptake in brain indicating an up-regulation of P-gp.

## OP17 An^18^F-labeled derivative of baclofen for imaging GABA_B_ receptors in mouse brain

### R. Naik, H. Valentine, R. F. Dannals, D. F. Wong, A. G. Horti

#### Division of Nuclear Medicine and Molecular Imaging, Department of Radiology, The Johns Hopkins University School of Medicine, Baltimore, 21287 USA

##### **Correspondence:** A. G. Horti

**Aim:** GABA (4-aminobutanoic acid) is the major inhibitory neurotransmitter in the central nervous system. GABA_B_ receptors are G-protein coupled receptor subtypes that play an essential role in various central and peripheral disorders. Molecules that modulate GABA_B_ receptors are of great medicinal interest for the possible treatment of many disorders and conditions including autism, alcohol dependence, anti-nociception, spasticity, fragile X syndrome, Down’s syndrome, Austin’s disease and retinal ganglion cell degeneration. A PET radiotracer for quantification of GABA_B_ receptors is not available yet. Our goal is to develop a PET radiotracer for imaging GABA_B_ receptors that would provide a significant advance in the understanding of autism and other GABA_B_-related CNS disorders. It could also facilitate novel GABA_B_ drug development.

**Methods:** New GABA_B_ agonists, fluoropyridylmethoxy analogues of baclofen, were synthesized. The *in vitro* potency of these new compounds was determined commercially. The most potent compound of the series, (*R*)-4-amino-3-(4-chloro-3-((2-fluoropyridin-4-yl)methoxy)phenyl) butanoic acid (**1**), was radiolabeled with ^18^F. The regional brain distribution of the radiolabeled [^18^F]**1** was studied in CD-1 male mice.

**Results** Substitution of the aromatic ring of *R*-baclofen with the fluoro 4-pyridyl ether moiety resulted in an increase (>10 times) of the GABA_B_ agonistic properties. The baclofen analog [^18^F]**1** was radiolabeled via the corresponding bromo-precursor with a radiochemical yield of 12-18%, specific radioactivity in the range of 330-515 GBq/μmol and radiochemical purity greater than 97%. In the animal experiments [^18^F]**1** entered the mouse brain (1% ID/g tissue) followed by washout. The accumulation of [^18^F]**1** in the mouse brain was inhibited (35%) by pre-injection of a selective GABA_B_ agonist, suggesting that the radiotracer binding is partially mediated by GABA_B_ receptors.

**Conclusion:** New GABA_B_ agonists, fluoropyridylmethoxy analogues of *R*-baclofen, were synthesized and radiolabeled with ^18^F. The mouse experiments with the most potent compound of the series, [^18^F]**1,** demonstrated the feasibility of *ex vivo* quantification of GABA_B_ receptors in the animal brain; however, its specific binding was insufficient for translation to human subjects. Our future research on GABA_B_ PET imaging will target radiotracers with improved specific binding and greater blood-brain barrier permeability.

## OP18 Measurement of blood brain barrier transport using radiolabeled antibodies

### G.Charest^1^, S. Ait-Mohand^1^, O. Sarrhini^1^, J. Rousseau^1^, D. Stanimirovic^2^, R. Hutchison^3^, D. Fortin^1^ and B. Guérin^1^

#### ^1^Department of nuclear medicine and radiobiology, Faculty of medicine and health sciences, Université de Sherbrooke, Sherbrooke, Québec, Canada, J1H 5N4; ^2^National Research Council Canada - Conseil national de recherches du Canada, Ottawa, Ontario, Canada K1A 0R6; ^3^biOasis, Richmond, British-Columbia, Canada V6X 2W8

##### **Correspondence:** B. Guérin

**Aim** Antibodies targeting transporters can function as carriers for the delivery of drugs in the brain for detecting and treating diseases of the central nervous system at the molecular level. Presently there is a need to develop efficient non-invasive method to validate the transport of these antibodies across the blood brain barrier (BBB). In this study, we investigated the potential of novel radiolabeled antibodies for measuring their engagement at the BBB. We compared different modes of delivery (Intravenous (IV), intra-arterial (IA)) to estimate their brain uptake and assess their biodistribution profiles.

**Methods:** The antibodies were conjugated to NOTA chelator for ^64^Cu-radiolabeling. The new [^64^Cu]NOTA-antibody conjugates were tested in normal rats using both IA and IV modes of administration with or without pre-injection of unlabeled material and were compared to a control [^64^Cu]NOTA-antibody.

**Results** We showed that the injection in the right carotid artery of our new ^64^Cu-NOTA-antibody conjugates resulted in a brief but important radioactivity exposure in the right brain hemisphere, which persisted after exsanguination. The right to left hemisphere ratios remained constant across the different concentrations of unlabeled conjugates and exceed that obtained with the control ^64^Cu-NOTA-antibody.

**Conclusion:** This study demonstrates that our new [^64^Cu]NOTA-antibodies allow for a transitory and specific brain uptake. These successful results are promising for the use of bi-specific radiolabeled antibody that can engage a target within the brain.

## OP19 Synthesis and ^18^F-Radiolabelling of Novel Benzoimidazotriazines for Imaging of Phosphodiesterase 2A (PDE2A)

### R. Ritawidya, B. Wenzel, R. Teodoro, M. Scheunemann, W. Deuther-Conrad, P. Brust

#### University of Groningen, University Medical Center Groningen, Department of Nuclear Medicine and Molecular Imaging, Hanzeplein 1, 9713 GZ Groningen, The Netherlands

##### **Correspondence:** R. Ritawidya

**Aim:** Cyclic nucleotide phosphodiesterases (PDEs) are a class of intracellular enzymes that inactivate the secondary messenger molecules cyclic adenosine monophosphate (cAMP) and cyclic guanosine monophosphate (cGMP). Thus, PDEs regulate the signaling cascades mediated by these cyclic nucleotides and affect fundamental cellular processes, such as proliferation, differentiation, migration, survival, and apoptosis. Accordingly, they are promising therapeutic targets. Since PDE2A was found to be related to a variety of tumors, it is our aim to synthesize novel PDE2A inhibitors based on the benzoimidazotriazine (BIT) moiety that might be a prospective lead compound for the development of an F-18 labelled ligand for PDE2A imaging with PET.

**Fig. 1 (abstract OP19).**
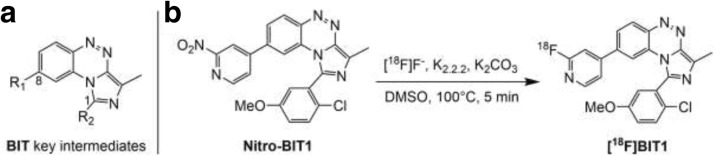
**a BIT** key intermediates, **b** Radiosynthesis of **[**^**18**^**F]BIT1**

**Methods:** Based on BIT key intermediates (Fig. 1a), a small series of novel fluorinated BIT derivatives was successfully prepared (overall in 7-10 steps) and the affinities towards PDE2A and other PDE subtypes were estimated. The most promising compound, **BIT1**, was radiolabelled by using the corresponding nitro precursor. The reaction was optimized by choosing different solvents, amounts of precursor, modes of heating (conventional or microwave), temperatures, and reaction times. Afterwards, best conditions (Fig. 1b) were transferred to an automated synthesis module (TracerLab FX2 N, GE Healthcare). The radiotracer was isolated by semi-preparative HPLC (Reprosil-Pur AQ column, 250×10mm, 46 % ACN/aqu. 20 mM NH_4_OAc, flow 5.5 ml/min) followed by purification with a Sep-Pak C18 Plus light cartridge and formulation in isotonic saline containing 10% ethanol.

**Results BIT1** showed a high affinity towards PDE2A (IC_50_ PDE2A3 = 3.33 nM) and selectivity over other PDE subtypes. [^**18**^**F]BIT1** was successfully synthesized with a radiochemical yield of 51.9 ± 1.3 % (*n* = 3), molar activities between 46 – 100 GBq/μmol and radiochemical purities of ≥ 99%.

**Conclusion:** Radiofluorination of a novel PDE2A ligand **[**^**18**^**F]BIT1** was obtained with appropriate radiochemical yield and molar activity. First biological investigations are planned to estimate the potential of **[**^**18**^**F]BIT1** as imaging agent for PDE2A.

**Acknowledgement**

1. Deutsche Forschungsgemeinschaft (German Research Foundation, Project Number: SCHE 1825/3-1).

2. Scholarship Program for Research and Innovation in Science and Technology Project (RISET-PRO)-Indonesia Ministry of Research, Technology and Higher Education.

## OP20 ^64^Cu-labelled anti-miRNA peptide nucleic acids as probes for molecular imaging of miRNA expression

### S. Croci^1^, A. Manicardi^2^, S. Rubagotti^3^, M. Bonacini^1^, M. Iori^3^, P.C. Capponi^3^, G. Cicoria^4^, M. Parmeggiani^1^, C. Salvarani^5^, A. Versari ^3^, R.Corradini^2^, M. Asti^3^

#### ^1^Clinical Immunology, Allergy, and Advanced Biotechnologies Unit, AUSL-IRCCS, Reggio Emilia, Italy; ^2^Department of Chemistry, University of Parma, Parma, Italy; ^3^Nuclear Medicine Unit, AUSL-IRCCS, Reggio Emilia, Italy; ^4^Medical Physics Department, University Hospital “S. Orsola-Malpighi”, Bologna, Italy; ^5^Rheumatology Unit, AUSL-IRCCS, Reggio Emilia, Italy

##### **Correspondence:** M. Asti

**Aim:** MiRNA are single stranded RNAs of 18-22 nucleotides and they have been found to be promising diagnostic and prognostic markers for several pathologies, such as tumors, neurodegenerative, cardiovascular and autoimmune disease. In the present work the development and characterization of the first anti-miRNA ^64^Cu-radiolabeled probes based on peptide nucleic acids for a potential non-invasive molecular imaging *in vivo* of giant cell arteritis are described.

**Methods:** MiR-146a and miR-146b-5p were selected as targets because they have been found up-regulated in this disease. Anti-miR-PNA and scramble-PNA probes were synthesized and linked to carboxyfluorescein or DOTA. The affinity of the probes for the targets was assessed by circular dichroism and melting temperature. Differential uptake and functional activity of fluoresceinated anti-miRNA probes were tested on BCPAP and A549 cell lines, expressing different levels of miR-146a and -146b-5p. DOTA anti-miRNA probes were then labelled with copper-64 to function as non-invasive molecular imaging tools and their stability and uptake were assessed in the same cell lines.

**Results** From circular dichroism studies it was possible to note that both PNA sequences (anti-miR-146a and -146b-5p) were able to recognize both miRNA sequences. The scrambled PNA sequence did not show any ability instead. Fluorescence of both BCPAP and A549 cells treated with anti-miR PNAs was higher than that of cells treated with the negative control scrambled-PNA. ^64^Cu-DOTA-anti-miR-146a PNA showed an almost doubled uptake in BCPAP cells with respect to the A549 cells after 42 hours. Conversely, the uptake of the ^64^Cu-DOTA-anti-miR-146b-5p PNA was comparable in the two cell lines. The uptake of the ^64^Cu-DOTA-scramble PNA was really low in both the cell lines and can be assigned to unspecific binding.

**Conclusion:** The experiments confirmed that anti-miR-146a PNA can selectively bound the miRNA target and its uptake is higher in miRNA overexpressing cells *in vitro*. ^64^Cu-anti-miR-146a PNA might be further investigated for non-invasive PET imaging of miR-146a and miR-146b-5p overexpressing diseases.

## OP21 Correlation between ^89^Zr-DFO-Trastuzumab-DM1 Delivery versus the Cytotoxicity and Response of T-DM1 on HER2 Expressing Breast Cancer Xenografts

### N. Al-saden^1^, Z. Cai^1^, C. Chan^1^, R.M. Reilly^1,2,3,4^

#### ^1^Department of Pharmaceutical Sciences, University of Toronto, Toronto, ON, Canada; ^2^Department of Medical Imaging, University of Toronto, Toronto, ON, Canada; ^3^Joint Department of Medical Imaging, University Health Network, Toronto, ON, Canada; ^4^Toronto General Research Institute, University Health Network, Toronto, ON, Canada

##### **Correspondence:** N. Al-saden

**Aim** Trastuzumab-DM1 (T-DM1; Kadcyla) is an antibody drug immunoconjugate (ADC) composed of the humanized IgG1 HER2 antibody trastuzumab linked to emtansine (DM1), a potent microtubule inhibitor. Our objective was to determine the correlation between the tumour uptake of ^89^Zr-DFO-T-DM1 assessed by imaging and biodistribution studies with the cytotoxicity of T-DM1 in vitro and in vivo response.

**Methods:** A panel of cell lines with different HER2 expression were treated in vitro with increasing concentrations of T-DM1 or trastuzumab (0-100 μg/mL) for 72 hours and the threshold of HER2 expression required for cytotoxicity was compared in clonogenic assays. In order to study the delivery of T-DM1 to tumours, we constructed T-DM1 labeled with the positron emitter ^89^Zr. Imaging of tumor uptake of ^89^Zr-T-DM1 was studied in mice bearing subcutaneous BT-474, MDA-MB-361, MDA-MB-231, and TrR1 xenografts. Mice were injected with a therapeutic dose of T-DM1 incorporating ^89^Zr-DFO-T-DM1 (10 μg, 6 ± 1.2 MBq). Mice were imaged on a micro-PET/CT system at 96 h post-injection. Tumour and normal-tissue biodistribution was determined. Another group mice of similar tumour models were treated with T-DM1 (3.6 mg/kg) every 3 weeks or with an equivalent volume of PBS for up to 7 weeks to determine tumour response.

**Results** Clonogenic assays demonstrated a significantly higher cytotoxicity of T-DM1 on HER2 positive tumour cell lines sensitive or resistant to trastuzumab. Resistant cell lines, such as TrR1 (breast cancer) and SK-OV3 (ovarian cancer) showed significantly higher cytotoxicity with T-DM1 at 10 μg/mL and 0.01 μg/mL, respectively. There was a strong correlation between HER2 density measured by flow cytometry and in vivo uptake of ^89^Zr-DFO-T-DM1 (incorporated into a therapeutic dose) with r^2^ = 0.9892. A strong and direct correlation between in vivo response (doubling time) and uptake of ^89^Zr-DFO-T-DM1 obtained with r^2^ = 0.8202. A strong indirect correlation was established between in vivo response (doubling time) and in vitro % survival, r^2^ = 0.9208.

**Conclusion:** We conclude that there was a strong correlation between tumour uptake of ^89^Zr-DFO-T-DM1, in vitro cytotoxicity, and in vivo response. ^89^Zr-DFO-T-DM1 has application for PET imaging of the delivery of T-DM1 to tumours in patients with HER2-positive BC. Imaging may inform on which patients are likely to benefit from T-DM1 treatment.

## OP22 Physicochemical and *in vitro* evaluation of a ^99m^Tc labelled NPY1 short analogue as potential breast cancer imaging agent

### M.E. Cardoso, E. Tejería, M. Terán, A. Rey

#### Área de Radioquímica, Facultad de Química, Universidad de la República (UdelaR), Uruguay

##### **Correspondence:** A. Rey

**Aim:** Neuropeptide Y receptors subtype Y1 (NPY1) are overexpressed in primary breast carcinomas and metastases and consequently radiolabelled agonists for these receptors would allow *in vivo* targeting of tumours for diagnostic and therapeutic purposes. With the aim to develop a potential ^99m^Tc breast cancer imaging agent based on the NPY1 structure we developed a short analogue (MA-Cys-Tyr-Arg-Leu-Arg-BPA-Nle-Pro-Asn-Ile-OH) containing the active sequence of the peptide with the addition at the amino-terminal end of a cysteine-mercaptoacetic acid moiety to coordinate the radiometal through the formation of a 3+1 nitrido complex.

**Methods:** Labelling was performed in two steps, namely: the preparation of a Tc(V)N precursor by reduction of pertechnetate (2,5-12,5mCi) with SnCl_2_ (0,1mg) in the presence of succinic dihydrazide (1mg) for 20 minutes followed by incubation (1 hour,80°C) of the nitrido precursor (in 1mL) with the peptide (100μg), tris(2-cyanoethyl) phosphine (0,5mg) and hydroxypropyl-gamma cyclodextrin (2mg). Radiochemical Purity (RP) of the final product was assessed by RP-HPLC using CH_3_CN/0,1%TFA and H_2_O/0,1%TFA as mobile phases. Lipophilicity was determined with n-octanol and phosphate buffer (0.1M, pH=7.4). Plasmatic protein binding (PPB) was measured by size exclusion chromatography. Stability in plasma and in labelling milieu was assessed by HPLC at 2 and 4 hours after labelling, respectively. Cysteine challenge (100 fold molar excess) was also performed. Cellular uptake, internalization and membrane binding studies were performed using t MCF-7(ATCC® HTB-22TM) cells.

**Results** Labelling of the peptide yielded a single species with a retention time of 11.4 min and a RP>90%. The complex was stable in reaction milieu and human plasma. Challenge with cysteine revealed no ligand exchange for up to 2 hours. Physicochemical evaluation showed a log P of -0.4+0.1 and a PPB of 16+2%. A cellular uptake of 3.1±0.2 (at 4 hours) was obtained. Internalization and membrane binding studies showed that 15.2% of the activity is bound to the membrane and 84.8% is internalised.

**Conclusion:** A ^99m^Tc-with agonist was obtained high RP, good stability and adequate physicochemical properties. In vitro characterization showed a promising uptake in MCF-7 cells. To establish whether it is a good diagnostic agent for breast cancer, it is necessary to deepen biological studies in tumour-bearing animals.

**Acknowledgements**

ANII (POS_NAC_2016_1_130455), Pedeciba-Química.

## OP23 ^177^Lu-DOTA-MGS5: the long-awaited theranostic probe for targeting cholecystokinin-2 receptor expression in medullary thyroid carcinoma and other tumours

### M. Klingler^1^, C. Rangger^1^, D. Summer^1^, J. Foster^2^, J.K. Sosabowski^2^, E. von Guggenberg^1^

#### ^1^Department of Nuclear Medicine, Medical University Innsbruck, Innsbruck, Austria; ^2^Centre for Molecular Oncology, Barts Cancer Institute, Queen Mary University of London, London, United Kingdom

##### **Correspondence:** M. Klingler

**Aim:** The theranostic use of mingastrin (MG)-based radioligands targeting cholecystokinin-2 receptor (CCK2R) expression in tumours is restricted by high kidney uptake or low enzymatic stability. Recently we have reported on the possibility to stabilise MG analogues against degradation by introducing specific amino acid modifications in the C-terminal receptor binding sequence (Trp-Met-Asp-Phe-NH_2_). In this study we have further refined our approach leading to a highly promising new targeting probe.

**Methods:** The new MG analogue DOTA-MGS5 was designed based on a combination of the substitutions applied in peptide derivatives previously developed by our group (1). The *in vitro* and *in vivo* characterisation after radiolabelling with In-111 and Lu-177 included stability analyses of the radioligands incubated in rat tissue homogenates and after injection into BALB/c mice. Receptor interaction in terms of receptor affinity and cell uptake was analysed using A431 human epidermoid carcinoma cells transfected with human CCK2R (A431-CCK2R) and mock-transfected cells (A431-mock). The targeting potential was evaluated by small animal dual modality single photon emission computed tomography (NanoSPECT/CT) as well as *ex vivo* biodistribution studies in tumour bearing BALB/c nude mice up to 4 h after injection.

**Results** DOTA-MGS5 and the complexes with different natural isotopes showed a high CCK2R affinity in the low nanomolar range. Interestingly, ^111^In-DOTA-MGS5 showed a particularly high internalisation (>40% and >80% after 1 h and 4 h respectively) into A431-CCK2R cells. The somewhat lower resistance against enzymatic degradation observed in rat tissue homogenates did not translate into a similar effect *in vivo*. Even though the stability in blood of ^111^In-DOTA-MGS5 was comparable to other radioligands previously studied (>80% intact peptide after 10 min), the tumour uptake with values of 23.5±1.3% IA/g at 4 h p.i. was incredibly high, leading to extraordinary performance in NanoSPECT/CT. Also for ^177^Lu-DOTA-MGS5 a similar stability in blood (>80% after 10 min) and tumour uptake (24.5±3.1% IA/g at 4 h p.i.) was found combined with favourable tumour-to-organ ratios of >3 for stomach and >6 for kidneys.

**Conclusion:** The combination of substitutions from earlier investigations allowed us to further improve our concept of stabilising MG analogues in the receptor-specific C-terminal sequence. ^177^Lu-DOTA-MGS5 with its extremely high tumour uptake seems to be the long-awaited theranostic probe for targeting CCK2R expression in tumours.

**Reference**

Klingler M, Rangger C, Summer D, Foster J, Sosabowski JK, von Guggenberg E, [2017], EJNMMI 44 (Suppl2), S228

**Fig. 1 (abstract OP23).**
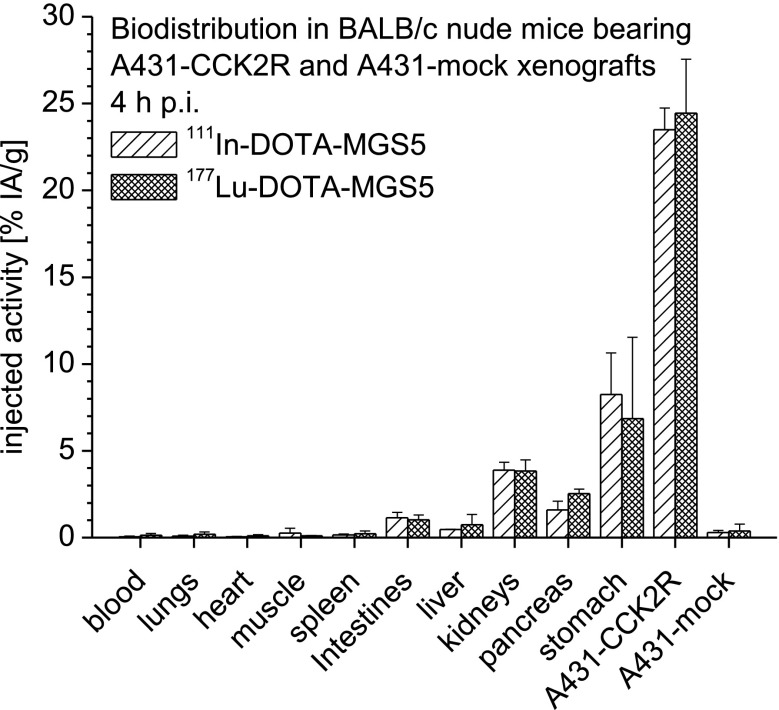
See text for description

## OP24 *In vivo* evaluation of biocompatible ^99m^Tc-bisphosphonate-coated MNPs designed as potential theranostic agents

### M. Mirković, M. Radovic, D. Janković, A. Vukadinović, M. Perić, Z. Milanović, S. Vranješ-Đurić

#### Laboratory for radioisotopes, Vinča Institute of Nuclear Sciences, University of Belgrade, Serbia

##### **Correspondence:** M. Mirković

**Aim:** Magnetic nanoparticles (MNPs) have the application potentiality in innovative diagnostic and therapeutic modalities because of their multifunctionality [1,2]. MNPs coated with two hydrophilic bisphosphonate ligands, i.e., methylene diphosphonate (MDP) and 1-hidroksietan diphosphonate (HEDP) were synthetized and labeled with ^99m^Tc. The aim was to examine if the above nanosystems, can be used as theranostic nanoagents, for hyperthermia application and nuclear diagnostic imaging.

**Methods:** The bisphosphonate coated MNPs were synthesized using co-precipitation method [3]. The heating ability for cancer hyperthermia therapy application was quantified through the specific power absorption (SPA) measurement. Radiolabeling of both bisphosphonate-coated Fe_3_O_4_ nanoparticles with ^99m^Tc were carried out using SnCl_2_ as a reducing agent. ^99m^Tc-MNPs were additional used for i*n vitro* stability studies in saline and human serum and *in vivo* biodistribution studies in normal Wistar rats.

**Results:** The obtained SPA values for Fe_3_O_4_-MDP and Fe_3_O_4_-HEDP are presented in Table 1. Radiolabeling of both bisphosphonate-coated Fe_3_O_4_ nanoparticles with ^99m^Tc was performed at high yields without purification (> 95 %). Incubation of both radiolabeled preparations in saline and serum showed that the bisphosphonate-iron oxide bonding was very stable with only 10 and 15 % of ^99m^Tc detaching from the iron oxide after 24 h, respectively. The highest uptake was observed at 1 h p.i. in the liver followed by the spleen (Fig. 1).

**Table 1 (abstract OP24).** SPA values of the aqueous dispersion of MNPs in the applied magnetic field, 23.9 kA/m

MNPs	SPA (W/g)		
	252 kHz	397 kHz	577 kHz
Fe_3_O_4_-MDP	67.3	143	183
Fe_3_O_4_-HEDP	55	78.5	131

**Fig. 1 (abstract OP24).**
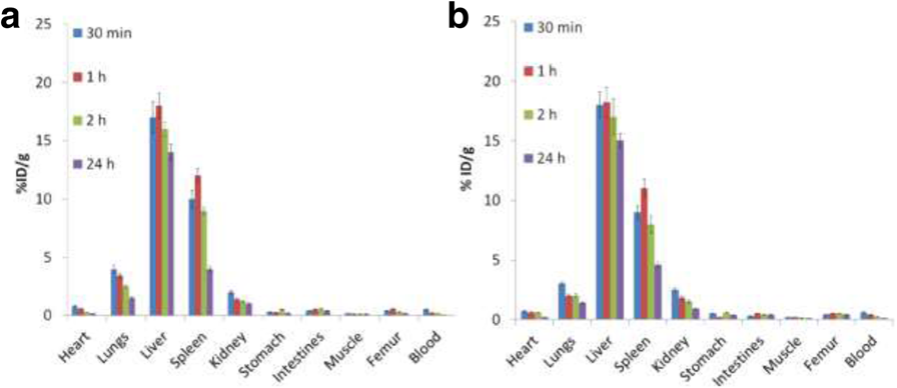
Biodistribution results of (**a**) ^99m^Tc-Fe_3_O_4_-MDP and (**b**) ^99m^Tc-Fe_3_O_4_-HEDP MNPs

**Conclusion:** The obtained results of specific power absorption demonstrate potential therapeutic applications of Fe_3_O_4_-MDP and Fe_3_O_4_-HEDP. Such radiolabeled biocompatible bisphosphonate MNPs with high labeling yield (> 95 %), *in vitro* and *in vivo* stability, showed the great promise as theranostic nanoagent which combines magnetic hyperthermia and SPECT imaging.

**References**

1. Yahara K, Ohguri T, Yamaguchi S, [2015], Int J Hyperthermia 31( 6): 600-608

2. Tromsdorf UI, Bruns OT, Salmen SC, Beisiegel U, Weller H, [2009], Nano Lett 9 (12): 4434-4440

3. Massart R, Cabuil VJ, [1987], Chim Phys 84: 967-973

## OP25 In vitro therapeutic efficacy of ^67^Ga-trastuzumab

### M. Faiz Othman^1^, M.S. Cooper^1^, C. Imberti^1^, M. T Ma^1^, V.J. Lewington^2^, P.J. Blower^1^, S.Y.A. Terry^1^

#### ^1^King’s College London, Department of Imaging Chemistry and Biology, School of Biomedical Engineering and Imaging Sciences, London, SE1 7EH, United Kingdom; ^2^Guy’s & St Thomas’ NHS Foundation Trust, Nuclear Medicine Department, London, SE1 9RT, United Kingdom

##### **Correspondence:** S.Y.A. Terry

**Aim:** Despite its desirable half-life and high-energy Auger electrons, ^67^Ga therapy has been neglected due to lack of suitable chelators and targeting molecules. With the advent of ^68^Ga-PET, excellent new chelators allow us to re-evaluate ^67^Ga for therapy. Previously, we showed that ^67^Ga causes DNA damage in cell-free systems and and cell kill in non-targeted cell studies. Here, we expand this work by targeting ^67^Ga to breast cancer cells using trastuzumab and comparing toxicity to well-described Auger electron emitter, ^111^In.

**Methods:** Trastuzumab, dialysed against 50 mM EDTA, washed and recovered in metal-free HEPES buffer, was conjugated with tripodal tris(hydroxypyridinone) (THP) (3.23 mg trastuzumab, 0.8 mM H_3_THP-Ph-SCN; 260 μL) and purified by size exclusion chromatography (SEC). DOTA-trastuzumab was also prepared similarly. DOTA-trastuzumab was labelled with ^111^In chloride at 40°C, pH 5.5 for two hours whereas THP-trastuzumab was labelling with ^67^Ga chloride at room temperature, pH 6.5, for 15 minutes. SEC purification was performed when labelling efficiencies were <95%. Radiopharmaceuticals (0.19 MBq/μg) were tested for their internalisation and effects on viability (dye exclusion) and clonogenicity of Her-2-positive (HCC1954) and –negative (MDA-MB-231) cell lines. Microautoradiography of cells in 18% gelatine was also performed.

**Results** Labelling efficiencies for ^67^Ga-THP-trastuzumab and ^111^In-DOTA-trastuzumab were 90% and 98%, respectively, giving 0.26±0.08 and 0.61±0.11 MBq/μg. At 4nM, ^67^Ga-THP-trastuzumab showed significantly higher cell binding uptake (10.69±1.32%) than ^111^In-DOTA-trastuzumab (6.15±1.64%; p=0.01) although the proportions internalised were equal; 62.08±1.43% and 60.78±15.45%, respectively. At 100nM however, cell binding percentages were equal: 1.15±0.98% ^67^Ga-THP-trastuzumab and 0.83±0.85% ^111^In-DOTA-trastuzumab. Controls ^67^Ga-THP-Ac and ^111^In-DOTA did not bind HCC1954 cells and binding of ^67^Ga-THP-trastuzumab and ^111^In-DOTA-trastuzumab in MDA-MB-231 cells was minimal (<0.2%). Microautoradiography showed that radioactivity bound to individual cells within the population varied considerably (from <10 to >90 silver grains per cell). Viability and clonogenicity decreased with increasing radiolabeled trastuzumab concentration, bound per cell. Radiopharmaceutical treatment of MDA-MB-231 cells or non-internalised activity in HCC1954 cells did not affect cell viability or clonogenicity. In HCC1954 cells, the surviving fraction after treatment at approximately 0.1 Bq/cell ^67^Ga-THP-trastuzumab, reduced to 0.38±0.13, more than for ^111^In-DOTA-trastuzumab (0.55 ± 0.16; p=0.03).

**Conclusion:** ^67^Ga-THP-trastuzumab and ^111^In-DOTA-trastuzumab both bind specifically HER2-positive cells and reduce their viability and clonogenicity. This shows ^67^Ga holds promise as a therapeutic radionuclide as a targeted radiopharmaceutical however non-homogeneous uptake amongst cells needs further investigation.

**Acknowledgments**

The project was in part supported by the Academy of Medical Sciences and Malaysian Ministry of Education.

## OP26 ^90^Y-labeled phosphate-coated magnetic nanoparticles designed for possible medical applications

### M. Radović, M. Mirković, D. Janković, A. Vukadinović, M. Perić, D. Stanković, Đ. Petrović, S. Vranješ-Đurić

#### Laboratory for radioisotopes, Vinča Institute of Nuclear Sciences, University of Belgrade, Serbia

##### **Correspondence:** M. Radović

**Aim:** Magnetic nanoparticles (MNPs) have been intensively used for a wide variety of biomedical applications. Only few reports on the *in vivo* biodistribution of functionalized MNPs with potential uses in magnetic hyperthermia (MHT) therapy are available [1, 2]. Radiolabeled MNPs coated with hydrophilic phosphate ligands, i.e., imidodiphosphate (IDP) and inositol hexaphosphate (IHP), were developed as multifunctional agents to localize both radioactivity and magnetic energy at a tumor site.

**Methods:** MNPs were synthesized by co-precipitation of ferric and ferrous salts in a basic solution [3, 4]. The heating ability of MNPs was quantified through the specific power absorption (SPA) measurements. The MNPs were labeled with 37 MBq ^90^YCl_3_, at room temperature for 1 h and were used in i*n vitro* stability studies in saline and human serum and *in vivo* biodistribution studies in normal Wistar rats.

**Results** The SPA values obtained for synthesized MNPs (46–81 W g^-1^) in different physiological media indicated their possible application in hyperthermia treatment (Fig. 1). Both types of coated MNPs were ^90^Y-labeled in a reproducible high yield (>98 %) and exhibited high *in vitro* stability in saline and human serum. The results of biodistribution, showed high uptake in the liver and spleen as well as *in vivo* stability up to 72 h. (Fig. 2).

**Fig. 1 (abstract OP26).**
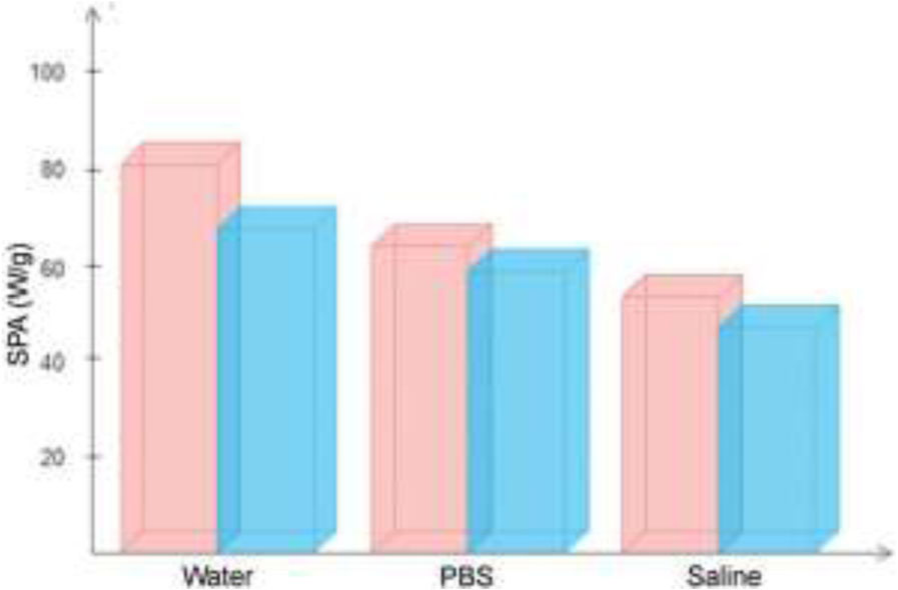
SPA values of Fe_3_O_4_-IDP (red) and Fe_3_O_4_-IHP (blue) MNPs under 23.9 kA m^-1^ and 577 kHz

**Fig. 2 (abstract OP26).**
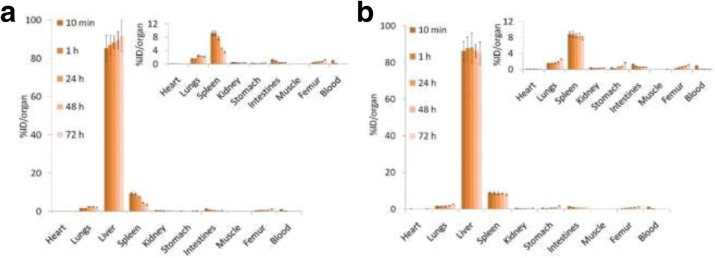
Biodistribution results of (**a**) ^99^Y-Fe_3_O_4_-IDP and (**b**) ^99^Y-Fe_3_O_4_-IHP MNPs

**Conclusion:** ^90^Y-coated MNPs were radiolabeled for two purposes: to use the radiotracer to obtain an accurate biodistribution profile of the MNPs and to produce potential radiotherapeutical agents. Both ^90^Y-labeled phosphate-coated MNPs exhibited favorable properties that justify further investigations toward their potential use in combined radiotherapy–hyperthermia cancer treatment.

**References**

1. Mitra A, Nan A, Line BR, Ghandehari H, [2006], Curr Pharm Res 12: 4729-4749.

2. Goya GF, Grazu V, Ibarra MR, [2008], Current Nanoscience 4(1): 1-16.

3. Massart R, Cabuil V, [1987], J Chim Phys 84: 967-973.

4. Radović M, Mirković M, Perić M, Janković D, Vukadinović A, Stanković D, Petrović Đ, Bošković M, Antić B, Marković M, Vranješ-Đurić S, [2017], J Mater Chem B 5**:** 8738-8747.

## OP27 Automation of FDG QC on Tracer-QC system

### R. Nair, A. Lebedev, A. Elizarov

#### Trace-Ability, Inc., 6160 Bristol Pkwy., Suite 200, Culver City, CA 90230, Canada

##### **Correspondence:** A. Elizarov

**Aim:** Quality Control (QC) of PET tracers is the most labor-intensive part of clinical tracer production. Tracer-QC system is being used in the US for complete automation of QC for FDG production meeting the requirements set by United States Pharmacopeia. Here we present the technology developed to address European Pharmacopeia requirements for radiochemical and chemical purity.

**Methods:** A QC automation system has been developed that consists of Tracer-QC device integrated with an HPLC. The HPLC includes a quaternary pump, UV-Vis detector and a radioactivity detector. Notably, no electrochemical detector is included. An innovative coupling was developed that provides for automated HPLC injection of the radioactive sample by a pipette from the deck of Tracer-QC robot. The above described system is enabled by a consumable cartridge that incorporates all reagents needed for analysis of color, clarity, residual kryptofix, ethanol, acetonitrile, bacterial endotoxin, radioactivity concentration, radiochemical purity (including ^18^F-fluoride and ^18^F-FDM), half-life, residual concentration of Cl-DG. Pre-column derivatization of sugars with PMP reagent was utilized to quantify Cl-DG content. Robotic component of Tracer-QC system allows for precise and fast mixing of the liquids.

**Fig. 1 (abstract OP27).**
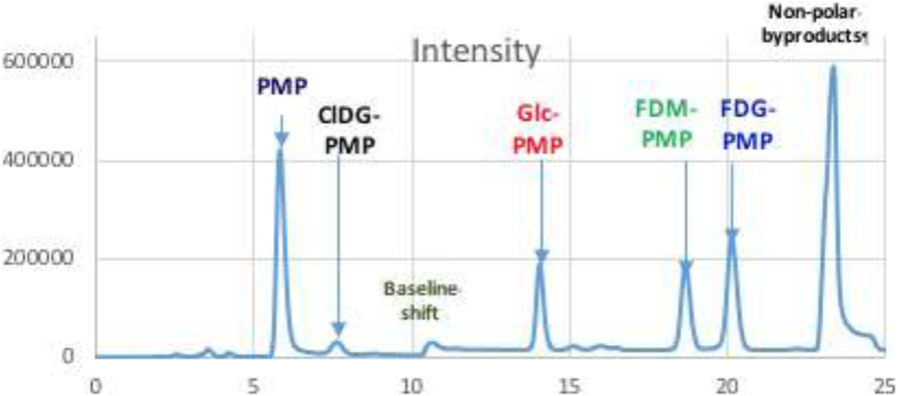
HPLC analyses of the mixture of sugars commonly analyzed in FDG quality control

**Results** The system presented here allowed for quantification of all impurities commonly analyzed in the production of FDG in Europe, while keeping the analysis time under 45 minutes. The derivatization and separation method was optimized to separate derivatized Cl-DG, Glucose, FDM and FDG with resolution exceeding Pharmacopeia requirements (Fig. 1). Limit of quantification of Cl-DG was determined to be 15 ppm, Limit for detection for ^19^F-FDG is ~1 ppm. ^18^F-FDG was fully converted to ^18^F-FDG – PMP derivative which was detected on the radiation detector. TLC analysis confirmed that ^18^F-FDG – PMP is the only radioactive product of the derivatization, with no competing decomposition.

**Conclusion:** These developments enabled an automated QC platform that contains all hardware needed for analysis of FDG as well as other ^18^F-based tracers. On this platform all analyses and sample manipulations are performed automatically, providing for maximum walk-away time – the user sets up a consumable cartridge and adds one sample. Then they only need to come back to collect the complete report. It is expected that such systems will have a dramatic impact in both routine production and development of new PET tracers.

**Disclaimer**

Reported Research was supported by the National Cancer Institute of the National Institutes of Health under Award Number R44CA192499.

The content is solely the responsibility of the authors and does not necessarily represent the official views of the National Institutes of Health.

## OP28 Simultaneous determination of the potentially toxic chemical impurities in the radiopharmaceuticals by capillary electrophoresis

### D. Antuganov^1^, Y. Antuganova^1^, T. Zykova^1^, R. Krasikova^2^

#### ^1^National Almazov Medical Research Centre, Saint-Petersburg, Russian Federation; ^2^N.P. Bechtereva Institute of Human Brain, Russian Academy of Science, Saint-Petersburg, Russian Federation

##### **Correspondence:** D. Antuganov

**Aim:** The implementation of recently introduced metal-mediated “late-stage” approaches for nucleophilic n.c.a. ^18^F-labelling of non-activated aromatics into routine production of the radiotracers is currently on-going. These new strategies employed or copper (**II**)^1^ catalysts that combined with stannyl- or Ni-based^2,3^ precursors. Moreover, classical kryptofix is often replaced by tetraethylammonium hydrogen carbonate (TEAHC)^2,3^. Therefore there is an urgent need in the development of simple analytical methods to control the removal of these toxic impurities from the final preparations until the allowable limits. Here we suggest the application of capillary electrophoresis (CE) for simultaneous determination of Cu, Ni, Sn and TEAHC in the analytical sample.

**Methods:** CE analysis was performed using «Capel-105M» system that equipped with UV spectrophotometric detector. The sample was loaded via electrokinetic injection (10 s, 25 kV) under following conditions: column 60 cm × 75 μm; background electrolyte: benzimidazole 20 mmol/L + acetic acid 40 mmol/L; UV 230 nm, separation voltage 25 kV, 20 °C. Stock solutions of Ni^2+^, TEAHC, Cu^2+^ and Me_3_SnCl in normal saline at a concentration of 10, 1000, 100 and 200 ppm respectively were prepared. Calibration standards were than prepared by serial dilution of the stock solutions. Before analysis the samples were fourfold diluted by deionized water. Under these conditions the retention times (RT) for Ni^2+^, TEAHC, Cu^2+^ and Me_3_SnCl were 4.4, 5.0, 5.6 and 7.8 min. All the peaks were well distinguished from that of sodium (3.9 min).

**Results** The conditions for simultaneous determination of toxic impurities in the buffer solution by CE technique CE analytical procedure have been developed. Our data (Fig. 1) show that the suggested method had a high sensitivity with detection limit for impurities according to ICH Guideline of Elemental Impurities Q3D.

**Fig. 1 (abstract OP28).**
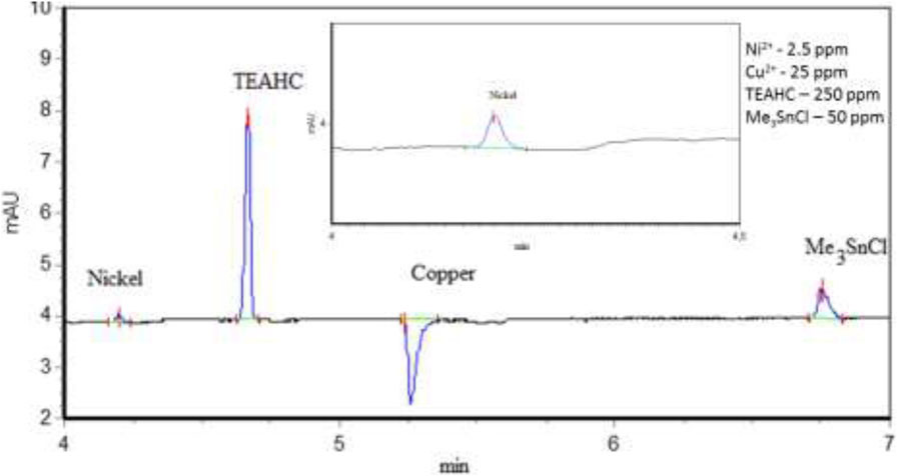
See text for description

**Conclusion:** New analytical CE method was proposed for routine quality control of the radiotracers, synthesized via metal-mediated reactions. The method is fast, simple, reliable and can be easy adapted to any CE equipment. This research was supported by RFBR grant № 16-54-12062\16.

**References**

[1]. Preshlock S et al., [2016], Chem. Rev. 116: 719-766

[2]. Zischler J et al., [2017], Chem. Eur. J. 23:3251-3256

[3]. Craig AS, [2017], Eur. J. Med. Mol. Imaging, 44(S2): 5382

## OP29 Evaluation of factors influencing the Ga-68 yield and Ge-68 breakthrough of a SnO_2_ based Gallium-68 generator

### SM Rubow, JS le Roux

#### Western Cape Academic PET/CT Centre, Stellenbosch University, Stellenbosch, South Africa

##### **Correspondence:** SM Rubow

**Aim:** Breakthrough of Ge-68, and the yield of Ga-68 are important aspects of Ge-68/Ga-68generator function. At the Western Cape Academic PET/CT Centre, SnO_2_-based Ga-68 generators produced by iThemba LABS are used. These generators are eluted with 0.6 M or 1 M HCL and Ge-68 breakthrough has been observed. The aim of this study was to evaluate the influence of the age of the generator, the number of elutions performed, and the interval between elutions on Ga-68 yield and Ge-68 breakthrough in order to optimise use of Ga-68.

**Methods:** Elution records of 7 generators, used between January 2013 and July 2017 were reviewed. The first 5 generators were eluted with 1 M HCl, and the last 2 generators with 0.6 M HCl. Ge-68 breakthrough was measured after decay of eluted Ga-68. The age of the generator and the time interval between elutions were calculated and plotted against breakthrough and yield.

**Results** The number of elutions per generator ranged between 28 and 151, and the generators were used for periods ranging from 166 to 372 days. Yield within the first month were between 119 % and 133 % of the nominal Ge-68 activity on the column for generators eluted with 1 M HCl, and 107 to 110 % for those eluted with 0.6 M HCl. By 160 days the yields had decreased to 106 % to 110 % with 1 M HCl elutions and 80 % to 99 % with 0.6 M HCl eluant. Three generators, including those eluted with 0.6 M, showed Ge-68 breakthrough within the first 10 days of use. By 160 days age, the Ge-68 breakthrough varied between 0.02 % and 1.0 %, with the lower acidity eluant giving the second highest value. There were no correlation between interval between elutions and Ge-68 breakthrough and Ga-68 yield.

**Conclusion:** Due to irregular intervals between elutions, it is difficult to compare elution yield at various ages of generators. All generators however clearly functioned poorer with increased use. The increasing Ge-68 breakthrough, and especially breakthrough early in the lifespan of the 2016 and 2017 generators is worrying, as it creates long-living radioactive waste. Elution with a less acidic eluant seems to provide a slightly lower yield but also lower Ge-68 breakthrough.

## OP30 iTLC Method for Analysis of ^68^Ga Radiophamaceuticals

### A.G. Makichyan, A.A. Larenkov

#### Burnasyan Federal Medical Biophysical Center, Moscow, Russia

##### **Correspondence:** A.A. Larenkov

**Aim:** Development of ^68^Ga radiophamaceuticals (RPs) requires a lot of tests to be made during labeling conditions optimization, stability evaluation, etc. It is critically important not only to evaluate radiochemical purity (RCP) value, but to know exact value of every radiochemical impurity content. There’s no doubt that TLC analysis is the most convenient method for the determination of all the radiochemical species content, given that the TLC system is adequate. Previously new method for determination of RCP of ^68^Ga-RPs was developed [1]. The method is handy, fast and informative with a good correlation with pharmacopoeial and other frequently used methods. The method allows to determine ^68^Ga-colloid, unbounded ^68^Ga and labeled molecule content with a single strip. The method can be routinely used with ^68^Ga-PSMA-617, ^68^Ga-DOTA/NODAGA-RGD/TOC/-TATE, etc. However the method has a drawback: sometimes adequate chromatographic separation is not available with acyclic chelators (DATA/HBED-CC/DTPA, etc.). Also molecular weight has an impact on *Rf* of the complexes (main peak shift can be about ±0.3 *Rf*). The aim of this study is to develop a new method allowing to overcome these drawbacks.

**Methods:** ^68^Ge/^68^Ga generator (Cyclotron Ltd, Obninsk, Russian Federation) was used. All chemicals and solvents were of high-purity or pharmaceutical grade and were purchased from Sigma-Aldrich or Panreac. Radiopharmaceutical precursors (PSMA-617, DOTA/NODAGA-RGD/TOC/-TATE, etc.) were purchased from ABX. iTLC-SG strips of different manufacture periods were used (Pall/Varian/Thermo). PET-MiniGita (Raytest) radio-TLC scanner was used for analysis of radioactivity distribution.

**Results** The decision was made to develop a method, which allows to obtain retention factors as follows: *Rf*=0.0-0.1 for ^68^Ga-colloid, *Rf=*0.5-0.6 for unbounded ^68^Ga and for labeled molecules *Rf*=0.9-1.0. This separation pattern was obtained for labeled compounds listed above using mixtures with w:o = 1:1 or 1:3 (w – water, saline; o – acetonitrile/ethanol/methanol). The presence of small amount of acid (TFA/HCl/HNO_3_, ect.) is essential. This amount should be kept very precise to provide pH 1.6±0.4. The most informative peak separation can be obtained with saline:acetonitrile mixture (1:1 ÷1:3) containing 0.06-0.08% TFA. Experiment details and critical features will be presented. This system is applicable for the most of ^68^Ga-RPs under study. It was found that chromatographic pattern is not the same when using iTLC-SG strips of different manufacture periods. This fact should be carefully taken into account.

**Conclusion:** New highly effective TLC method for ^68^Ga labeled compounds analysis was developed. The method is applicable during pharmaceutical development and routine clinical practice.

**References**

Larenkov A.A., Maruk A.Ya. [2016] WASET International Journal of Chemical, Molecular, Nuclear, Materials and Metallurgical Engineering 10:1120-1127.

**Fig. 1 (abstract OP30).**
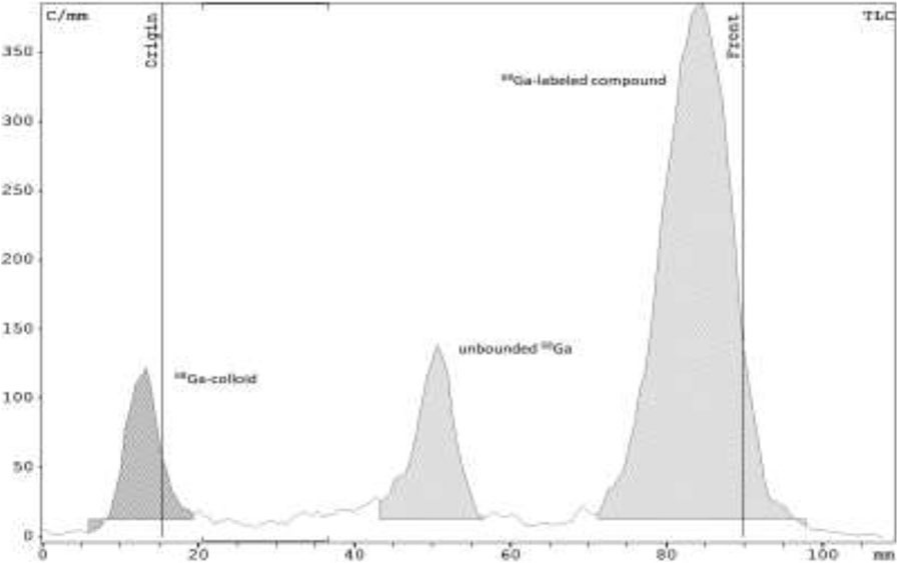
See text for description

## OP31 Optimzation of [^18^F]-FPSMA1007 Synthesis HPLC free on Fastlab Platform

### E. Cazzola, P. Colombo, A. Dangelo, D. Peruzzi, A. Purgato, J. Amico, A. Peroni, M. Malachini, C. Frassanito, G. Gorgoni

#### Radiopharmacy and Cyclotron Dept, Sacro Cuore Don Calabria Hospital, Negrar, (Vr), Italy

##### **Correspondence:** E. Cazzola

**Aim:** Over the past years, many different PET agents have been developed to investigating on Prostate Cancer (PC) to make the non invasive approach a reality, in order to replace the biopsy and the related complications. The PC is the more common cancer that affect the male population. Due to the high incidence of this pathology are mandatory to investigate on a fluorine-18 tracer that give the possibility to overcame the gallium-68 tracers limitations.[1] The aim of this study is to optimize an automatic synthesis for [^18^F]FPSMA1007 on Ge FASTLab® module, in a stable high yield with a wide range of inlet activity, and setting up a faster, efficient and EU Pharmacopoeia compliant quality control to shorten the time of product realize. [2]

**Methods:** The synthesis method is based on one step synthesis using a new precursor commercialized by ABX and is tuned on Ge FASTLab® synthesizer. All the reagents are included on a single use cassette. The [^18^F]Fluorine was trapped on QMA and eluted with a mixture of TBAHCO_3_/ACN or K222/ACN/K_2_CO_3_ and after drying at 125°C on synthesis reactor, the ABX precursor dissolved in DMSO was added to proceed with the nucleophilic [^18^F]-Fluorination. The reaction mixture was heated up at 95°C for 10 min after the reaction step the mixture was cooled at 35°C to starting the purification step followed by formulation. The total process takes place on 37 minutes. HPLC analysis was performed on an Agilent 1260 Infinity HPLC equipped with an Agilent 1260 UV detector and a Raytest gamma-ray detector, controlled with Gina Star software V.5.9. The analysis was performed on a 4,6x100 Eclipse Plus C18 3.5μm (Agilent) in isocratic conditions using CH_3_CN and 0.1 % TFA (70/30, run time 15 min). TLC analysis was performed on Merk Silica Gel 60 on PET (10x5 cm, developed over 2/3 of the plate), mobile phase MeOH/NaCl 0.9% a Perkin Elmer Cyclone. Other analysis were performed according to European Pharmacopoeia “Radiopharmaceutical Preparations”.

**Results** Two different elution solution was used to compare the final process yields, at the same time, high activity runs were performed, in different inlet activity range, to evaluating the yield and product stability in final formulation. For stability study a range of 1-2.5 GBq/ml radioactive concentration was evaluated at room and at 40°C for up to 12h. According to final product formula specification the synthesis yield was stable on range 30-50 % at the inlet activity range (55-170 GBq) with a very high A_m_ ( 800-3500 GBq/ 훍mol) at EOS. The radiochemical purity for all the runs was > 96 % and chemical impurities were < 0.1 mg/V. The new HPLC/TLC method allow to make the quality control in terms of general indications of European Pharmacopoeia Monographs.

**Conclusion:** All the synthesis performed by using the K222 elution solution shows slightly lower yield compared to TBA, at the same time any difference, in quality control profile and stability were found, all the products collected were stable and in high chemical/radiochemical purity. The HPLC and TLC methods setup allow to perform a complete purity evaluation on less than 15 min.

**References**

1. Giesel F.L., et al [2017], EJNMMI 44:678-688.

2. Martin R., et al [2017], J label comp radioph 60: 594.

## OP32 Synthesis and evaluation of radioiodinated and astatinated prosthetic groups for bioorthogonal conjugation to antibodies for nuclear imaging and therapy

### L. Navarro^1^, S. Gouard^1^, C. Alliot^2^, M. Chérel^1^, F. Pecorari^1^, F. Guérard^1^, JF. Gestin^1^

#### ^1^CRCINA, Inserm, CNRS, Université d’Angers, Université de Nantes, Nantes, France; ^2^GIP Arronax - Saint-Herblain, France

##### **Correspondence:** F. Guérard

**Aim:** Radioiodine and astatine are increasingly studied for therapeutic or diagnostic purposes in nuclear medicine [1]. Particularly, ^211^At (t_1/2_ = 7.2 h, α-emitter) is a promising radioisotope for targeted α-particle therapy. To associate these radiohalogens to cancer targeting biomolecules (peptides, antibodies), the conventional approach consists in performing the radiohalogenation of a precursor bearing a *N*-Hydroxysuccinimidyl (NHS) ester function for conjugation to the biomolecule [2]. However this approach requires basic aqueous conditions leading to competitive hydrolysis of the NHS ester, resulting in sub-optimal conjugation yields (<50%). To overcome this issue, we have initiated the investigation of click chemistry based bioorthogonal conjugation approaches aiming at increasing the coupling yields and improving the radiolabelling procedure.

**Methods:** A series of bifunctional precursors bearing clickable functionalities (tetrazine, azide or alkyne) were specifically designed for radiolabelling with radioiodine and ^211^At. Bioorthogonal ligation kinetics of the labelled prosthetic groups were evaluated on model peptides bearing the complementary clickable functions (*trans*-cyclooctene (TCO), bicyclononyne (BCN), alkyne) for identification of the best system, and transferred to antibody radiolabelling.

**Results** All ligation reactions tested on model peptides produced quantitative yields from less than 1 minute to 9 hours with the following reactivity order: [tetrazine-TCO] > [copper(I) catalyzed alkyne-azide] > [tetrazine-BCN] > [azide-BCN], as expected from literature data[3]. The fastest system [tetrazine-TCO] was then tested on an anti-CD138 mAb, providing quantitative radiolabelling yields (>99%) without need of purification within less than a minute, confirming the high efficacy of this approach. Immunoreactivity against CD138 was preserved (80 ± 2%).

**Conclusion:** The results obtained highlight the higher efficiency of click chemistry in comparison with the conventional approach. They open perspectives for quantitative radiolabelling with reduced conjugation time that may facilitate the transfer of ^211^At-labelled antibodies to clinical applications. Assessement of the in vivo behaviour of such radiolabelled biomolecules is now in progress to fully validate this promising approach.

**References**

1. Adam M, Wibur S, [2005], Chem. Soc. Rev., 34: 153-163

2. Zalutsky MR, Narula AS, [1988], Int. J. Rad. Appl. Instrum. A., 39: 227-232

3. Lang K, Chin JW, [2014], ACS Chem. Biol., 9: 16-20

**Fig. 1 (abstract OP32).**
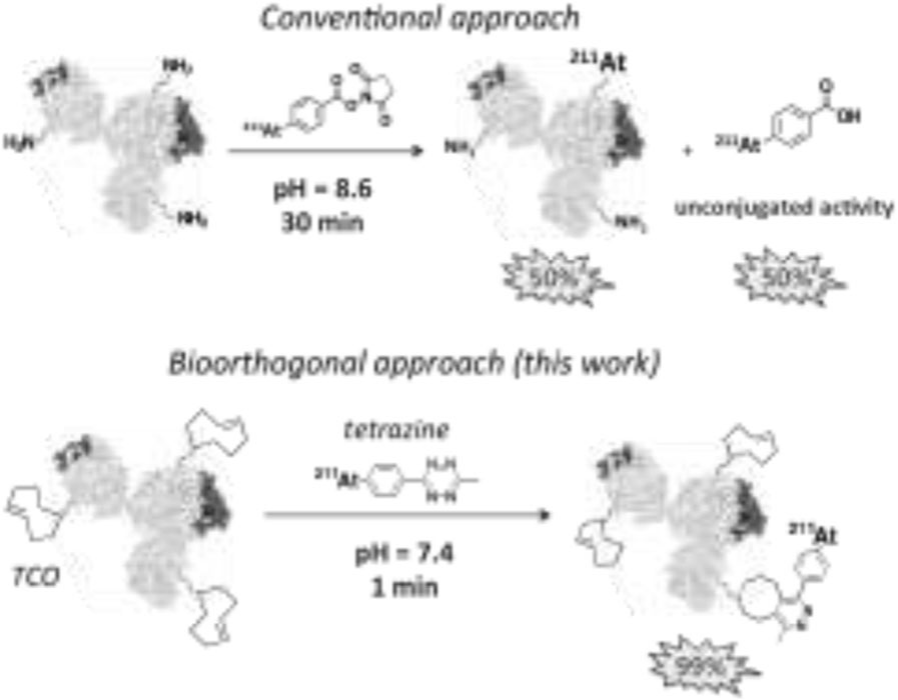
Comparison between the “NHS-lysine” and “bioorthogonal” radiolabelling approach

## OP33 Relative biological effectiveness (RBE) of ^177^lutetium-NOTA-panitumumab F(ab’)_2_ fragments for radioimmunotherapy of pancreatic cancer cell lines

### A. J. Boyle^1^, Zh.Cai^1^, D.W. Hedley^2^, R.M. Reilly^1^

#### ^1^Leslie Dan Faculty of Pharmacy, University of Toronto; Toronto, ON, Canada; ^2^Centre for Pharmaceutical Oncology, Toronto, ON, Canada

##### **Correspondence:** A. J. Boyle

**Aim:** The 5-year survival for PnCa patients is only 6% due to late-stage diagnosis and inadequate treatment options. There is an urgent need for new treatment strategies. Human epidermal growth factor receptor (EGFR) is overexpressed on up to 90% of PnCa tumours. Panitumumab is an antibody against EGFR that can be modified with a NOTA chelator for radiolabeling with ^177^Lu to treat PnCa cells as a radioimmunotherapy agent (^177^Lu-RIT). This study aims to determine the RBE of ^177^Lu-RIT compared to γ-radiation and examine EGFR positivity as a predictor of PnCa response to ^177^Lu-RIT.

**Methods:** *Flow cytometry*: EGFR density was measured on AsPC-1, PANC-1, MiaPACA-2, and Capan-1 cell lines. Cells were incubated with panitumumab for 60 min, then with Alexa Fluor 647 anti-human IgG immunoconjugates for 30 min at 4°C, then run on a flow cytometer. *Clonogenic survival assays*: Cells were treated with γ-radiation (0-8Gy) or ^177^Lu-RIT (0-3MBq/72nM) then seeded in into 6-well plates and allowed to grow at 37°C, 5% CO_2_, for 14 days. Then colonies were fixed/stained and counted. *Subcellular fractionation*: Cells were treated with ^177^Lu-RIT (1MBq/72nM) for 1, 3, 4, and 24 hours. Radioactivity associated with cell surface, cytoplasm, and nucleus was separated then measured in a γ-counter.

**Results** EGFR density was +++, +++, +, and ++, for AsPC-1, PANC-1, MIAPaca-2, and Capan-1 cells, respectively. Clonogenic survival after γ-radiation was reduced to 10% (D_10_) for AsPC-1, PANC-1, MIAPaca-2, and Capan-1 cells at 3.2 Gy, 5.0 Gy, 4.0 Gy, and 2.5 Gy. ^177^Lu-RIT caused D_10_ at 3.1MBq, 1.1MBq, 6.0MBq, and 1MBq for AsPc-1, PANC-1, MIAPaca-2, and Capan-1 cells, respectively. Subcellular fractionation studies showed that PANC-1 had the highest nuclear localization of ^177^Lu-RIT and results from these studies allowed microdosimetry to be performed to convert MBq to Gy. The D_10_ after ^177^Lu-RIT was 16.9 Gy, 5.7 Gy, 23.3 Gy, and 8.2 Gy for AsPc-1, PANC-1, MIAPaca-2, and Capan-1, respectively, resulting in RBE of 0.2, 1.0, 0.2, and 0.3.

**Conclusion:** RBE of ^177^Lu-RIT is less than γ-radiation and varies significantly between PnCa cell lines with no correlation to EGFR density. EGFR positivity is not an adequate predictor of response to RIT with ^177^Lu-RIT.

## OP34 Biological Assessment of a Radiolabelled LXXLL-Peptide for Breast Cancer Theranostics

### F. Vultos^1^, M. Belo^1^, M. Scheepstra^2^, F. Silva^1^, C. Fernandes^1^, M. C. Oliveira^1^, F. Mendes^1^, J.D.G. Correia^1^, L. Brunsveld^2^, D. Viertl^3^, L. Gano^1^

#### ^1^Centro de Ciências e Tecnologias Nucleares, Instituto Superior Técnico, Universidade de Lisboa, Estrada Nacional 10, km 139.7, 2695-066 Bobadela LRS, Portugal; ^2^Eindhoven University of Technology-TU/e, Eindhoven, Neederlands; ^3^Service of Nuclear Medicine, University Hospital of Lausanne, CH-1011 Lausanne, Switzerland

##### **Correspondence:** L. Gano

**Aim:** Breast cancer (BC) remains the most common invasive cancer diagnosed among women and the second most frequent cause of cancer-related death in women worldwide. The majority of BC cases are hormone-responsive and approximately 75% express the estrogen receptor (ER), a well-established biomarker for prognosis and guiding treatment of patients. ER has also been used as a target for BC imaging. In spite of the improved BC survival rate due to endocrine therapies that modulate ER action, resistance to treatment is a major clinical concern. Thus, new molecular imaging agents and more effective therapies are needed to improve BC management. Theranostics is particularly suitable for that purpose as the ER status profiling acquired by imaging can be used to targeted treatment. Peptides containing the LXXLL sequence have demonstrated high ER affinity and could be of potential value to develop targeted radiopharmaceuticals. Thus, our goal was to assess the potential value of a radiolabelled LXXLL peptide as theranostic agent for BC. For that purpose, we have selected from the literature a peptide with recognized ER affinity and have radiolabelled it with two different radionuclides, ^111^In and ^125^I.

**Methods:** A LXXLL-peptide was synthesized and conjugated to the bifunctional chelator DOTA by microwave-assisted solid phase synthesis. The peptide conjugate was radiolabelled with ^111^In. ^125^I-labelling was performed by direct radioiodination in the histidyl residue using the oxidative method of chloramine-T. ER binding affinities were evaluated by a fluorescent polarization assay with the corresponding inactive In(III) complex or iodinated peptide. Cellular uptake was assessed in MCF-7 (ER+) and MDA-MB-231 (ER-) human breast cancer cells. Biodistribution was assessed in tumor-bearing Balb/c mice induced with MCF-7 cells and microSPECT imaging studies are underway.

**Results:** The LXXLL peptide was successfully radiolabelled with both radionuclides with high radiochemical yield and purity at high specific activity. Chemical identity was ascertained by comparing its HPLC profile with that of the inactive congeners. Inactive LXXLL-peptide derivatives retained ER binding affinity. Both radiolabelled peptides have showed rapid and high uptake in MCF-7 cells (ER+). Biodistribution studies in tumor- bearing mice indicated high in vivo stability, fast blood clearance and high uptake in ER rich organs and MCF-7 xenografts.

**Conclusion:** The favourable biological performance of radiolabelled LXXLL peptides in cellular and tumor-bearing mice models suggests their potential as theranostic agents.

**Acknowledgements**

F. Vultos thanks FCT for PhD grant (SFRH/BD/84509/2012) and COST CM 1004. The work was supported by EXCL/QEQ-MED/0233/2012 and UID/Multi/04349/2013.

## PP01 Optimization of Biological Quality Control of Radiochemical Precursors used for Radiopharmaceuticals Formulations – A step towards Good Radiopharmacy Practice

### A. Mitra^1^, S. Lad^2^, S. Kaisar^2^, S. Kulkarni^2^, S. Banerjee^1,2^

#### ^1^Medical Cyclotron Facility, BRIT, TMH Annexe, Parel, Mumbai 400 012, India; ^2^Radiation Medicine Centre, BARC, TMH Annexe, Parel, Mumbai 400 012, India

##### **Correspondence:** A. Mitra

**Aim:** The present study was designed to develop and validate methods for biological quality control (bacterial endotoxin and sterility testing) for various radiochemical precursors (RCPs) viz. ^68^GaCl_3_, ^177^LuCl_3_, H^18^F, Na^99m^TcO_4_, Na^188^ReO_4_ and CH_3_COO^90^Y. These RCPs are considered to be active pharmaceutical ingredient (API) for various radiopharmaceutical formulations. The validation of biological quality control procedures for RCPs is a mandatory requirement for small scale radiopharmaceutical preparations (SSRP) in order to maintain good radiopharmacy practice (GRPP).

**Materials:** ^68^GaCl_3_, ^177^LuCl_3_, H^18^F, Na^99m^TcO_4_, Na^188^ReO_4_, and CH_3_COO^90^Y, Lysate(λ:0.03EU/mL), Tris-base (0.25/0.2/0.4/2N), Ca^++^ and Mg^++^ free PBS(PO_4_^-^ ion concentration: 7.8nM). Cultures of *S.aureus, P.aeruginosa, B. subtilis, C.albicans*.

**Methods:** The endotoxin limit(EL) for RCPs was fixed at 5EU/mL, based on the volume used for radiosynthesis and alert limit. BET assays were performed by gel-clot method. Maximum valid dilution (MVD) was calculated considering the sensitivity of lysate and EL. Sterility testing was validated by direct inoculation method using FTM and SCD media. Positive controls for *S.aureus, P.aeruginosa, B.subtilis, C.albicans* were set up at bacterial concentration <100 CFU (serial dilution- spread plate method) and corresponding negative controls were set up with and without RCPs. Positive and negative controls of RCPs were set up considering single patient dose (125-150 and 50-100 mCi of RP in a volume of 0.5 and 2.0mL meant for therapeutic and diagnostic radiopharmaceutical formulations respectively).

**Results** In BET assay, ^177^LuCl_3_ and CH_3_COO^90^Y exhibited inhibition due to extreme pH conditions. Inhibition could be resolved by neutralizing with endotoxin negative (<0.25EU/mL) 0.25N Tris-base at first dilution. ^68^GaCl_3_ exhibited enhancement due to presence of excess Na^+^ and inhibition because of extreme pH. The enhancement in BET test was resolved using endotoxin negative PBS as diluting agent instead of LRW and inhibition was taken care of by neutralization with 0.25N Tris-base. BET assay of Na^99m^TcO_4_, Na^188^ReO_4_ and H^18^F, did not demonstrate any inhibition. In sterility testing, luxuriant growth was observed for positive control culture in the presence of 1-2 mL (50-100 mCi) of Na^99m^TcO_4_, Na^188^ReO_4_and H^18^F following incubation for 48 hours. Growth of all these microorganisms was inhibited (<100 CFU) in 0.5-1.0 mL ^68^GaCl_3,_^177^LuCl_3_ and CH_3_COO^90^Y. However, inhibition was reversed on neutralization with sterile Tris-buffer (0.2/0.4/2N). This was also confirmed by counting CFU by spread plate method. No growth was seen in any of these RCPs in negative controls.

**Conclusion:** The procedures for quantification of endotoxin limit and sterility tests of various RCPs used in radiopharmaceuticals formulations was standardized and validated.

## PP02 Production of ^64^Cu using indigenously developed solid target assembly in Medical Cyclotron Facility in Radiation Medicine Centre

### K. Kushwaha^1,2^, P. Maletha^1^, A. Mitra^3^, R. Kashid^1^, S. Kamble^4^, J. Babu^4^, K. Pathak^4^, N.K. Prasad^4^, S. Banerjee^1,2,3^

#### ^1^Radiation Medicine Centre, BARC, TMH Annexe, Parel, Mumbai 400012, India; ^2^Homi Bhabha National Institute, BARC, Anushakti Nagar, Mumbai 400094, India; ^3^Medical Cyclotron Facility, BRIT, TMH Annexe, Parel, Mumbai 400012, India; ^4^Technical Physics Division, BARC, Trombay, Mumbai 400085, India

##### **Correspondence:** K. Kushwaha

**Aim:** The GE- PET trace 800, a medical cyclotron facility in RMC provides proton beam of 16.5 MeV, with varying beam current from 1μA to 75 μA. This cyclotron has liquid and gaseous irradiation target cavities for production of non-metallic radioisotopes viz ^18^F, ^11^C,^13^N etc. In order to extend the utility of the cyclotron for producing medically useful isotopes viz ^64^Cu, ^111^In etc using the solid targets, a prototype semi-automated solid target assembly has been designed and fabricated indigenously. In this study the feasibility of this target for irradiation, to produce ^64^Cu was tested. Materials: Natural Ni powder < 150 μm, 99.999% trace metal basis purchased from Aldrich, ORION gamma-ray spectrometer coupled with a high purity Ge-detector

**Methods:** A proton beam of 2 μA was irradiated for 30 min on natural Ni (electroplated in-house with thickness 120 μm). The parameter such as the vacuum, He pressure, γ field and neutron flux was observed at regular time intervals during irradiation. After irradiation, target disc was transferred into a lead capsule. The γ spectrum of irradiated sample was recorded with HPGe detector. Quantity of ^64^Cu produced was calculated by analysing the spectrum using InterWinner 7.0 software.

**Results** The vacuum was found to be stable indicating proper sealing of the solid target assembly with cyclotron port. The He gas pressure was constant showing no leak of coolant from the target assembly. The γ field and neutron flux measured was found to be 38 mR/h and 236 n/cm^2^/s respectively during the irradiation. The HPGe spectrum showed a peak at 1.34 MeV corresponding to ^64^Cu along with other peaks. The quantity of ^64^Cu produced was estimated to be 29 μCi at the EOB.

**Conclusion:** The indigenously developed solid target assembly was found to be compatible with GE-PET trace 800 cyclotron, having only liquid and gaseous target cavities. The target was successfully tested for irradiation at 2μA current for 30 m time duration with natural Ni target. This indigenous target can be used for production of higher amount of ^64^Cu while using enriched ^64^Ni with high current.

## PP03 Microfluidic reactor in a PDMS chip for [^18^F]F-radiopharmaceuticals

### L. Fernandez-Maza^1^, B Salvador^2^, D. Orta-Castello^1^, A. Corral^1^, I. Fernandez-Gomez^1^, A. Luque^2^, J.M. Quero-Reboul^2^

#### ^1^Centro Nacional de Aceleradores. Universidad de Sevilla, CSIC, Junta de Andalucia, Spain; ^2^Departamento de Ingenieria Electronica. Escuela Tecnica Superior de Ingenieria, Universidad de Sevilla, Spain

##### **Correspondence:** L. Fernandez-Maza

**Aim:** Polydimethylsiloxane (PDMS) is a cheap and easly available material for microfluidic devices for radiopharmaceutical synthesis. Literature describes interactions of this material with [^18^F]Fluoride (references 1,2,3,4). Some authors discard PDMS and others suggest the use of fluoropolymer thin films to reduce [^18^F]Fluoride adsorption. The aim of this work was to evaluate [^18^F]Fluoride retention and elution in a PDMS reactor chamber, when it is under heating and vacuum, normaly used for [^18^F] PET radiosynthesis.

**Methods:** Ten 30 μL reactor chambers of PDMS microchips for PET radiopharmaceuticals were tested to evaluate possible adsorption phenomena. No cover films were used, so the [^18^F]Fluoride interacted directly with the PDMS. Activities from 8 MBq to 2500 MBq of [^18^F]Fluoride were assayed. First, [^18^F]Fluoride was preconcentrated in a QMA resin and eluted with K_2_CO_3_ 6 mg/mL. The radioactive solutions (K_2_CO_3_ aqueous solution/acetonitrile 1:1) were pippeted manually into the reactors, heated until complete evaporation (100-140°C) with vacuum (700 mbar, Sykam S1021 pump) to mimic azeotropic distillation and nucleophilic substitution. An average temperature of 130°C was maintained during 10 minutes, measured by a coupled termocouple. [^18^F]Fluoride was then eluted with water for injection and the reactor chambers were dried with compressed air. Radioactivity was measured after filling, evaporation and water elution in a Comecer IBC Dose Calibrator.

**Results:**

**Table 1 (abstract PP03).** See text for description

PDMS Reactor	Loading [^18^F]F- Activity (MBq)	Activity After evaporation (MBq)	Elution Water for injection (MBq)	Residual activity at reactor after elution (MBq)
1	8.62	7.88	6.96	0.10
2	12.58	11.08	10.20	0.20
3	28.12	26.27	22.60	1.03
4	51.80	48.84	40.70	1.70
5	66.60	59.29	50.90	1.80
6	240.05	217.6	206.88	0.74
7	802.10	726.90	672.66	18.07
8	925.30	804.22	710.45	7.05
9	1257.01	1122.7	955.34	7.03
10	2544.80	2372.1	2152.03	7.40

**Conclusion:** In our experience, [^18^F]Fluoride was practically not adsorbed to PDMS when this material is under heating and vacuum conditions commonly used in [^18^F]Fluoride preconcentration and nucleophilic substitution. On the contrary, [^18^F]Fluoride was retained and almost completely eluted with water for injection, so we can suggest this cheap and easily available material for lab-on-chip Radiopharmacy.

**Refernces**

1. Zacheo A, Arima V. Pascali G, Salvadori PA, [2011], Microfluid Nanofluid 11:35-44

2. Zhou JW, Ellis AV, Voelcker NH, [2010], Electrophoresis 31(1):2-16

3. Leonardis F, Pascali G, Salvadori PA, Watts P, Pamme N, [2011] J. Chromatogr. A, 1218:4714-4719

4. Elizarov AM, et al. [2010], J Nucl Med 51(2):282-287

## PP04 Development and comparison of dual generator elution methods on a MultiSyn Synthesizer (iPHASE technologies Pty Ltd) - application to the synthesis of 68Ga-peptides

### L. Morandeau^1^, S. Poniger^2,3^, A.H. Asad^1^, J. Ioppolo^1^, S. Chan^1^, R.I. Price^1,4^

#### ^1^Department of Medical Technology and Physics, Sir Charles Gairdner Hospital, Perth, WA, Australia; ^2^iPHASE technologies Pty Ltd, Melbourne, VIC, Australia; ^3^The Austin Hospital, Melbourne, VIC, Australia; ^4^School of Physics, University of Western Australia, Nedlands, WA, Australia

##### **Correspondence:** L. Morandeau

**Aim:** Increasing patient demand for 68Ga-peptides in our facility has created the need for higher number of doses per batch produced. We have investigated several options for dual generator elution for the synthesis of 68Ga-DOTATATE on an iPHASE MultiSyn Synthesizer, using two ITG 68Ge/68Ga generators.

**Methods:** The efficiency of three double elution methods of ITG generators using HCl 0.05M were compared:

(i) Fractionated elution: The elution profile of one ITG generator was first determined. The most concentrated fractions from two generators were then collected and measured. (ii) “In series” elution: Two generators were connected in series and the elution profile determined. The optimum fraction and rate of elution were established.
(ii) Pre-concentration ^(1)^: the individual eluates from both generators were passed through a Strata SCX cartridge, and the trapped 68Ga eluted with acidified 5 M NaCl. The synthesis of 68Ga-DOTATATE was then optimised for elution methods (i) and (iii). TLC of the crude reaction mixture was performed before purification. After synthesis, activity of all the synthesis components were measured and decay corrected to EOS.

**Results:** The % recovery for the three methods was determined as follows: double elution recovered activity/sum of activity recovered from 4 mL single elution of each generator (1-3 days prior) x 100. (i) Fractionated elution: 87% of 68Ga was recovered in 4.5 min from the collection of the 2-5 mL fractions from both generators. (ii) “In series” elution: the 2-8mL fraction was collected in 6.5 min, yielding 80% of recovered activity. (iii) The pre-concentration/elution method took 5.5 min and recovered 91% of the activity. 68Ga-DOTATATE synthesis: Using method (i) the precursor (25μg) in 1.5 mL 0.25M Na-acetate buffer was reacted with the 6 mL eluate (pH reaction 3.5-4) for 7 min at 90-95°C. The RCP of 68Ga-DOTATATE before purification was 98.4% (TLC). 68Ga-DOTATATE was obtained in 83% yield (n.d.c), 13% activity was found in the waste and was mostly accounted for the discarded fractions. Using method (iii), the precursor in 1 mL buffer + 3 mL Trace Select water was reacted with 0.8 mL eluate (pH reaction 3.5-4). Before purification, the RCP of 68Ga-DOTATATE was 95.6% (TLC). 68Ga-DOTATATE was obtained in 84.5% yield (n.d.c), 4.2% activity remained on the Strata X and 6% in the waste.

**Conclusion:** 68Ga-DOTATATE was successfully synthesized in excellent yields on a MultiSyn Synthesizer starting from the fractionated and pre-purification double elution methods of two ITG generators.

## PP05 Radiolabelling of DTPA-silk fibroin nanoparticles with ^111^In for nanoparticle biodistribution studies

### A.A. Lozano-Pérez^1^, M Alejandra Asensio Ruiz^2^, E. Fernández Muñoz^2^, Á.s García Aliaga^2^, A. Abella Tarazona^2^, T. Martínez Martínez^2^, L. Meseguer Olmo^3^

#### ^1^Departamento de Biotecnología, Instituto Murciano de Investigación y Desarrollo Agrario y Alimentario (IMIDA), Murcia, Spain; ^2^Unidad de Radiofarmacia. Hospital Clínico Universitario Virgen de la Arrixaca, Murcia, Spain; ^3^Regeneration and tissue repair Group at Catholic University of Saint Anthony (UCAM), Guadalupe, Murcia, Spain

##### **Correspondence:** T. Martínez Martínez

**Aim:** In the last decades the Silk Fibroin Nanoparticles (SFNs) have received considerable attention for drug delivery due their high binding capacity for various drugs and controlled drug release properties. Up to date, multiple formulations of SFNs with different encapsulation methods and drugs have been described but it is still unknown their biodistribution *in vivo* after intravenous administration or tissue injection. Our aim in this work is to label the SFNs with a radioisotope to allow the visualization of the SFNs distribution *in vivo*. Post-labelling approach is proposed, nanoparticles are first functionalized with DTPA and then incubated with ^111^InCl_3_.

**Methods:** SFNs were prepared by a nanoprecipitation in MeOH [1]. The DTPA-functionalized silk fibroin nanoparticles (DTPA-SFNs) were prepared by amide coupling reaction between the amine groups of SFNs and one of the carboxylic groups of DTPA by using the EDC/NHS activating system [2].

**Fig. 1 (abstract PP05).**
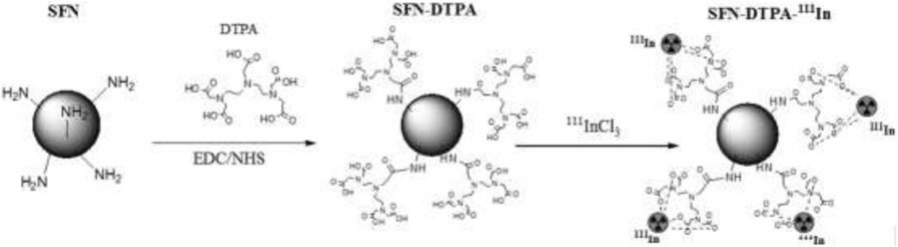
See text for description

Suspensions of DTPA-SFNs (Zaverage = 191.1 ± 0.7 nm, PdI 0.115 and ζ= -32.1 ± 0.6 mV; pH 7.5) at concentration of 7 mg/mL were labelled with activities ranging from 22 to 79 MBq of ^111^InCl_3_ (Mallinckrodt Radiopharmaceuticals Spain, S.L.U) at room temperature up to 3 hours under stirring. After incubation, suspensions were washed twice with deionized water. Labelling yield was calculated as radioactivity in SFNs divided by total radioactivity (SFNs and supernatant).

**Results** Radiolabelling yield was 63.69±2.15 % (*n* = 8). Activity measured in supernatants after washing was under 3% (*n* = 8). ^111^In labelled nanoparticles were stable in suspension along the incubation experiment.

**Conclusion:** The coupling of DTPA followed by incubation with ^111^In chloride could be a promising method for SFNs radiolabelling. Further studies are needed in order to optimize the radiolabelling procedure, including stability tests, cytotoxicity in cell cultures and biocompatibility *in vivo*.

**References**

1. Lozano-Pérez A.A., *et al.* [2014] *Int.J Nanomedicine*, 9, 4507–4520.

2. Montri Ratanajanchai, *et al*, [2014] *J. of Colloid and Interface Science*, 415, 70–76.

## PP06 First approaches to radiolabelling of silk fibroin nanoparticles with ^99*m*^Tc

### A.A. Lozano-Pérez^1^, E. Fernández Muñoz^2^, M.A. Asensio Ruiz^2^, Á. García Aliaga^2^, A. Abella Tarazona^2^, T. Martínez Martínez^2^, L. Meseguer Olmo^3^

#### ^1^Departamento de Biotecnología, Instituto Murciano de Investigación y Desarrollo Agrario y Alimentario, Murcia, Spain; ^2^Unidad de Radiofarmacia. Hospital Clínico Universitario Virgen de la Arrixaca, Murcia, Spain; ^3^Regeneration and tissue repair Group at Catholic University of Saint Anthony (UCAM), Guadalupe, Murcia, Spain

##### **Correspondence:** T. Martínez Martínez

**Aim:** Nanoparticles have recently gained great interest in biomedical applications. Among them, silk fibroin nanoparticles (SFNs) present excellent properties as vectors for drug delivery and several biomedical applications have been recently reviewed [1]. *In vivo* tracking of these nanoparticles is an actual challenge for researchers and labelling with radioisotopes would be an attractive alternative to other invasive biodistribution studies [2]. This work describes how silk fibroin nanoparticles (SFNs) are capable of adsorbing ^99m^Tc compounds by using different strategies. Radioisotope labelling of SFNs was optimized in terms of radioisotope/SFNs ratio (MBq/mg SFN), time and temperature of incubation.

**Methods:** SFNs were prepared by a previously described nanoprecipitation method [3]. Aqueous suspensions of SFNs (Zave=168.1±2.5 nm, PdI = 0.095, ζ= -21.2±0.7 mV; pH 7.5) at two concentrations (5 and 12.5 mg/ml) were radiolabeled with activities ranging from 250 to 1050 MBq of ^99m^Tc-DTPA (Technescan DTPA, Mallinckrodt Radiopharmaceuticals Spain, S.L.U.) and ^99m^Tc-HMPAO (Exametazima, Radiopharmacy Laboratory Ltd), either at room temperature or 4 °C, up to 3 hours under constant stirring and recovered by centrifugation and washed twice with deionized water in order to remove weakly bounded radioisotopes. Labelling yield was calculated as radioactivity in SFNs divided by total radioactivity (SFNs and supernatant). Radioisotope-labelled silk fibroin nanoparticles were further characterized in diameter (Z-Average) and Z-potential (ζ) by DLS.

**Results** Results of labeling yield in the assayed conditions are summarized in the following figure.

**Fig. 1 (abstract PP06).**
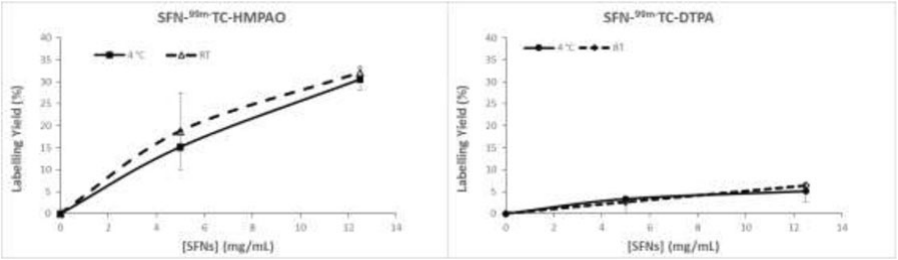
See text for description. *RT=Room temperature, *n* = 8

**Conclusion:** According to our results, labelling with ^99m^Tc-HMPAO reaches higher yields (32.1 ±1.4 %) compared to ^99m^Tc-DTPA (6.4±0.4 %). Higher concentration of nanoparticles shows higher isotope retention. Temperature of incubation does not significantly affect labelling yields. On one hand, DLS measurements showed that ^99m^Tc-HMPAO-SFNs are slightly bigger in diameter and negatively charged than the unloaded SNFs. On the other hand ^99m^Tc-DTPA-SFNs are significantly bigger in diameter but are less negatively charged than the unloaded SNFs. These are very preliminary outcomes and further studies are needed to optimize the radiolabelling procedure.

**References**

1. Zhao Z., et al.. [2015] Int. J. Mol. Sci. 16, 4880-4903.

2. Pereira MA, et al. [2008] Eur J Pharm Sci. 33(1):42-51.

3. Lozano-Pérez AA, et al. [2017] Int J Pharm. 518(1-2):11-19.

## PP07 Investigation of radiopharmaceutic potential of a new radiolabelled graft polymer for using in the therapy and in the molecular imaging on albino wistar rats

### Buket Ateş^1^, Uğur Avcıbaşı^1^, Perihan Ünak^2^, Fikriye Gül Gümüşer^3^, Sinan Akgöl^4^, Volkan Tekin^2^

#### ^1^Department of Chemistry, Faculty of Art and Science, Manisa Celal Bayar University, 45140 Manisa, Turkey; ^2^Ege University, Institute of Nuclear Sciences, Department of Nuclear Applications, Bornova, İzmir 35100, Turkey; ^3^Department of Nuclear Medicine, Manisa Celal Bayar University, School of Medicine, 45030 Manisa, Turkey; ^4^Department of Biochemistry, Faculty of Science, Ege University, 35100, İzmir, Turkey

##### **Correspondence:** Buket Ateş

**Aim:** The main reason to use the nanoparticles is to make active substrate not to decay at the injection site, to target impact zones, to provide continuous active substrate oscillation and to improve the bioeffect of administration of drug. To assist intravenously applied anticancer drugs in overcoming barriers (such as Chemical, Biological, Physical, and Clinical Barriers) and to improve the balance between their efficiency and their toxicity, a large number of drug delivery systems have been developed over the years, ranging in nature from ‘simple’ liposomes, polymers [1] and micelles, to bacterially derived ‘Minicells’ and temporally targeted ‘Nanocells’ [2].

**Methods:** This study deals with grafted lysine aminoacid (Lys), metacryl derivate of a hydrophobic side chained aminoacid containing poly(2-hydroxyethylmethacrylate) based magnetic graft-Lys-poly(HEMA) nanoparticles, and the attempts to radiolabel this compound with a appropriate radionuclide (such as ^99m^Tc) and evaluate the usefulness of the resultant radiopharmaceutical for the diagnosis and management of malignant tumors using nuclear methods. Hydrophobic nature of the nanoparticle, possibly increases the uptake of nanoparticle in different organs and tissues.

**Results** Magnetic graft-Lys-poly(HEMA) was labeled with ^99m^Tc and radiopharmaceutical potential was investigated using animal models in this study. Quality control procedures were carried out using thin layer radiochromatography (TLRC). The labeling yield of radiolabeled polymer, ^99m^Tc-m-graft-Lys-poly(HEMA), was found to be about 100%. Then, stability and lipophility studies were done for this radiolabeled polymer. The n-octanol/water partition coefficient (lipophilicity) of ^99m^Tc-m-graft-Lys-poly(HEMA) was determined. The lipophilicity was found to be 0.17. The results of the serum stability experiments demonstrated that approximately 100% of ^99m^Tc-m-graft-Lys-poly(HEMA) existed as an intact complex in the human serum within 240 min. Biological activity of ^99m^Tc-m-graft-Lys-poly(HEMA) was determined on female Albino Wistar rats by scintigraphy and biodistribution studies. The biodistribution study showed high uptake in the stomach, the pancreas, brain, ovarian, intestines and the breast.

**Conclusion:** In conclusion, ^99m^Tc-m-graft-Lys-poly(HEMA), which has diagnostic and therapeutic application potentials in nuclear medicine, was first radiolabeled using the SnCl_2_ method and investigated to evaluate its biodistribution. Radiolabeled m-graft-Lys-poly(HEMA) proved a useful tool for assessing the *in vivo* behavior of the drug in rats.

**References**

1. Kopecek J, Kopeckova P, Minko T, Lu ZR, Peterson CM, [2001], J Control Release 74:147-158.

2. Sengupta S, Eavarone D, Capila I, Zhao G, Watson N, Kiziltepe T, Sasisekharan R, Nature [2005] 436: 568-572.

## PP08 Production of Lutetium-177 DOTATATE/PSMA-617 on a MultiSyn radio-synthesizer module for use in Molecular Radiotherapy

### A.H. Asad^1^, L. Morandeau^1^, S. Chan^1^, J. Ioppolo^1^, S. Poniger^2,3^ and R.I. Price^1,4^

#### ^1^Department of Medical Technology and Physics, Sir Charles Gairdner Hospital, Perth, Western Australia; ^2^iPHASE technologies Pty Ltd, Melbourne, VIC, Australia; ^3^The Austin Hospital, Melbourne, VIC, Australia; ^4^School of Physics, University of Western Australia, Nedlands, WA, Australia

##### **Correspondence:** A.H. Asad

**Aim:** ^68^Ga-PSMA & ^68^Ga-DOTATATE PET/CT have been established as a useful modality in the diagnostic imaging of prostate cancer (PCa) and neuroendocrine tumours (NETs) respectively. Their therapeutic “companions” ^177^Lu-PSMA and ^177^Lu-DOTATATE have also been developed for the treatment of these tumours (Nanabala et al., 2016; Maus et al., 2014). The aim of this work was to develop the production and quality control of ^177^Lu-PSMA and ^177^Lu-DOTATATE for clinical applications. In order to avoid hand doses to staff and reduce the risk of ^177^Lu contamination, we investigated the automated synthesis of ^177^Lu-PSMA and ^177^Lu-DOTATATE on a MultiSyn radiosynthesizer (iPHASE technologies Pty Ltd). The preliminary development phase presented here was investigated with 10 GBq per synthesis.

**Methods:** The syntheses were carried out on a MultiSyn automated synthesizer located into a dedicated shielded hotcell. ^177^Lu-DOTATATE was prepared by incubating ^177^LuCl_3_ (~10 GBq in 500μL of 0.04M HCl) with 120 μg of DOTATATE in 500μL of 0.4 M sodium acetate/50μL of 20% ascorbic acid solution at 85^o^C for 30 min. After cooling, 0.5 mL of DTPA (1 mg DTPA in 0.9%NaCl) was added. The final product was diluted and transferred through a sterile filter (Cathivex GV) into the final collection vial containing 25 mg of sodium ascorbate. Radiochemical purity as well as radiochemical stability of the product was determined by radio-TLC and radio-HPLC methods.

**Results** ^177^Lu-DOTATATE was prepared in yield 86.7% ± 0.2% (*n* = 3) with a radiochemical purity greater than 99%. The final product passed all recommended quality control specifications (pH, sterility, pyrogen test & bubble test). The synthesis of ^177^Lu-DOTATATE was completed in ~ 45 min. A similar approach for the ^177^Lu-PSMA is in progress.

**Conclusion:** High activities of ^177^Lu-DOTATATE were successfully prepared in good yields using a MultiSyn radiosynthesizer, passing all QC requirements. The automated synthesis was performed in a dedicated hotcell for proper radiation protection. This optimised method will be used to provide ^177^Lu-labelled PSMA and DOTATATE for clinical use at Sir Charles Gairdner Hospital in 2018.

**References**

1. Nanabala R, Sasikumar A, Joy A, Pillai M, [2016]. JNMRT 7:5

2. Maus S, de Blois E, Ament SJ, Schreckenberger M, Breeman WAP, [2014], IJDI 1:1

## PP09 Production of norepinephrine transporter tracer, [^18^F]NS12137, via copper-mediated nucleophilic ^18^F-fluorination

### S. Lahdenpohja, T. Keller, A. K. Kirjavainen

#### University of Turku, Radiopharmaceutical Chemistry Laboratory, Turku PET Centre, Turku, Finland

##### **Correspondence:** S. Lahdenpohja

**Aim:** [^18^F]NS12137 (3-[(6-[^18^F]fluoro-2-pyridyl)oxy]8-azabicyclo[3.2.1]octane) is a highly selective norepinephrine transporter (NET) tracer [1]. Recently [^18^F]NS12137 has been produced via electrophilic pathway [1]. Herein we introduce the synthesis of protected [^18^F]NS12137 by a copper-mediated ^18^F-labelling starting from a stannylated NS12137 precursor (Fig. 1).

**Methods:** Radiolabelling was performed with a partly automated synthesis device, by applying a previously reported ^18^F-labelling procedure [2]. Dry [^18^F]KF/K_222_ complex was formed by azeotropic drying of the cyclotron produced [^18^F]fluoride at 120 °C. Cu(OTf)_2_(py)_4_ was added to the reaction vessel in anhydrous MeCN at room temperature and the reaction mixture was stirred for 10 minutes. The stannylated precursor was added to the reaction vessel in dimethylacetamide (DMA) or MeCN. When using DMA as a solvent, MeCN was first evaporated at 120 °C. The reaction vessel was then heated at 120 °C and samples for analytical radio-HPLC were collected during 30 minutes every 5 minutes. When using MeCN as a solvent, the reaction vessel was heated at 80 °C and samples for analytical radio-HPLC were collected during 75 minutes every 15 minutes. Deprotection has been performed by changing the solvent (DMA to THF), evaporating THF to dryness and then treating with 48 % HBr.

**Results** Protected [^18^F]NS12137 was successfully prepared from the stannylated precursor via copper-mediated nucleophilic [^18^F]fluorination. The radiochemical yield (RCY, non-decay corrected, based on radio-HPLC analysis of the crude reaction mixture) was up to 94% when using DMA as a reaction solvent after 5 minutes reaction. When MeCN was used, the RCY was up to 0.5% after 30 minutes reaction.

**Conclusion:** Protected [^18^F]NS12137 was produced with a high RCY by the copper-mediated synthesis. The RCY obtained is higher than with electrophilic ^18^F-fluorination. Future work will include deprotection and isolation of the product.

**Acknowledgements**

We are grateful to Dan Peters from DanPET AB (Sweden) for providing the stannylated NS12137 precursor. This work was supported by the Academy of Finland (grant no. 266891 and 307924).

**References**

1. Kirjavainen AK, Forsback S, López-Picon FR, Marjamäki P, Takkinen J, Haaparanta-Solin M, Peters D, Solin O, [2018], NMB, 56:39-46

2. Keller T, Krzyczmonik A, Forsback S, Kirjavainen AK, López-Picón FR, Takkinen J, Dollé F, Rinne JO, Haaparanta-Solin M, Solin O, [2017], J Label Compd Radiopham 60:S423

**Fig. 1 (abstract PP09).**
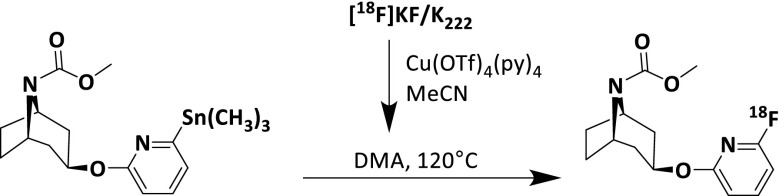
Synthesis of protected [^18^F]NS12137

## PP10 Production, applications and status of zirconium-89 immunoPET Agents; an IAEA new Coordinated Research Project

### A. R. Jalilian, J.A. Osso

#### Radioisotope Products and Radiation Technology Section, Department of Nuclear Sciences and Applications, International Atomic Energy Agency (IAEA), Vienna International Centre, PO Box 100, 1400 Vienna, Austria

##### **Correspondence:** A. R. Jalilian

Zirconium-89 has attracted huge interest and is used in tracing and quantification of slow biological processes and labelling of long half-live biomolecules such as monoclonal antibodies for pharmacokinetic studies and clinical trials. A new Coordinated Research Project (CRP), planned to be initiated by IAEA in late 2018 will investigate the targetry, irradiation data, separation and coordination chemistry of zirconium-89 by all participants from developed and developing countries. A detailed work plan on conjugation and 89Zr radiolabelling of biomolecules shall be addressed. The participation of 15 Member States for 4 years is expected in this project.

**Keywords**: IAEA, CRP, 89Zr, Production, Radiolabelling, Monoclonal antibody

**Fig. 1 (abstract PP10).**
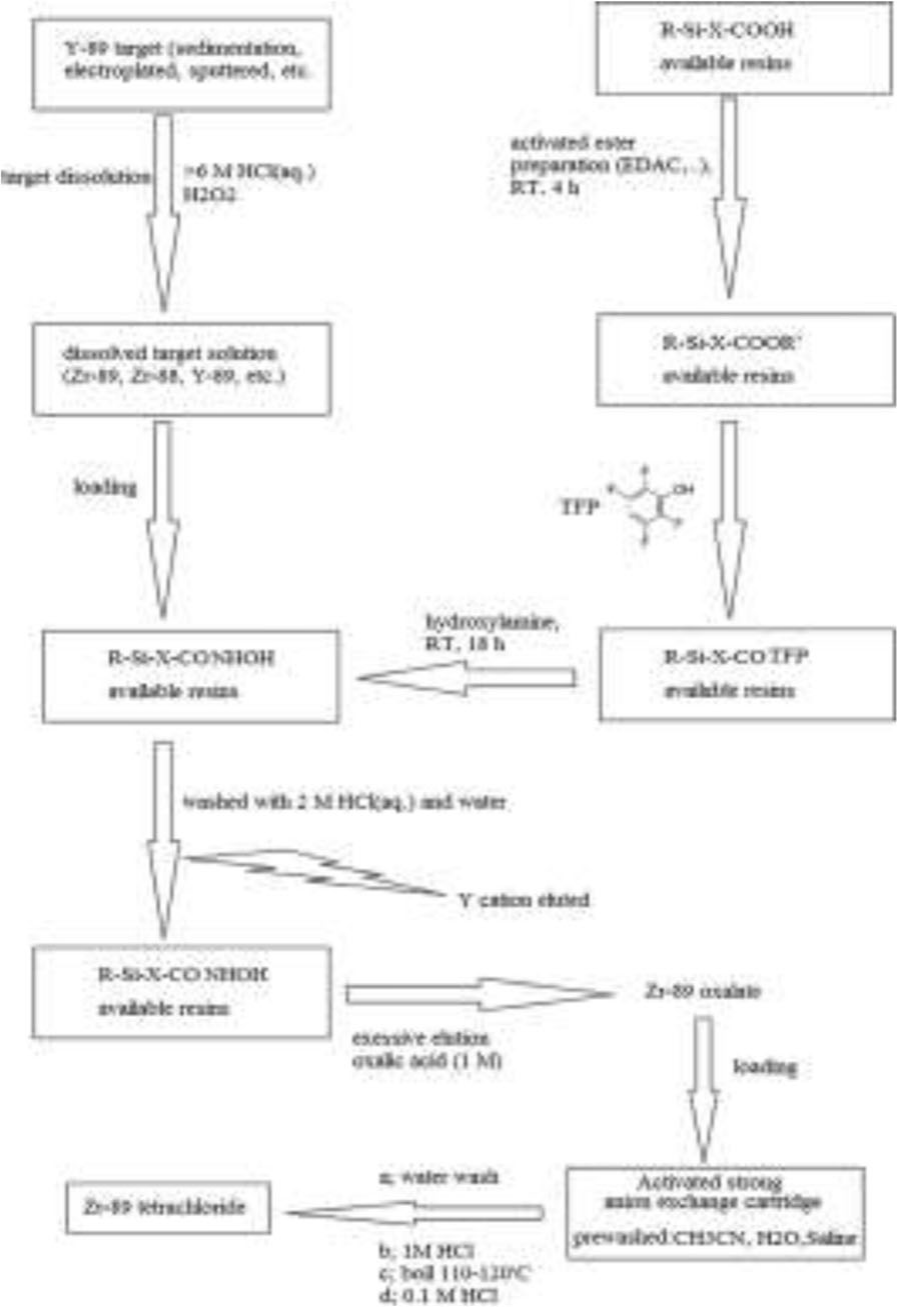
See text for description

**Fig. 2 (abstract PP10).**
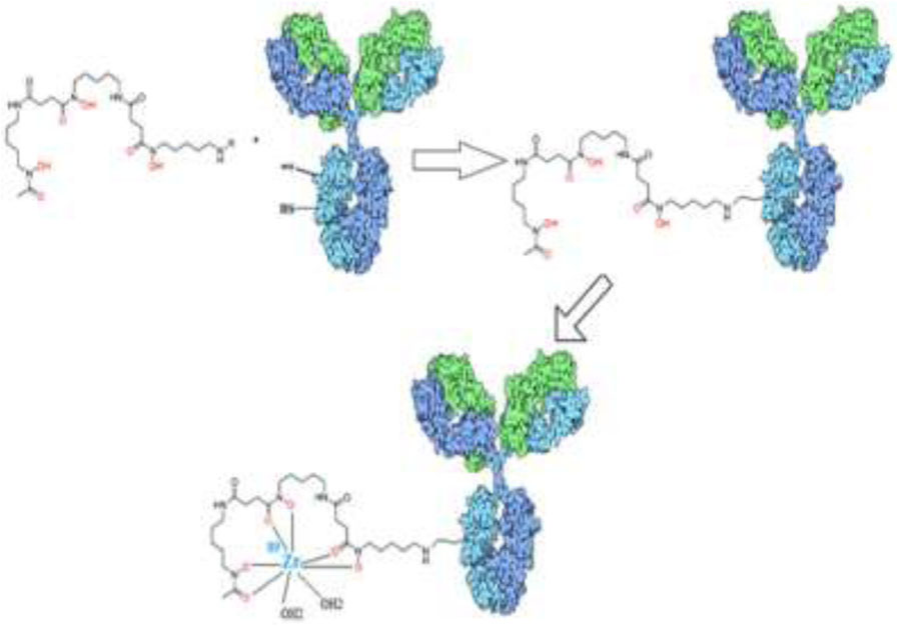
See text for description

**References**

1. Jalilian AR, Osso, Jr. JA, [2017], J Radioanal Nucl Chem 314:7–21

2. https://cra.iaea.org/cra/index.html

## PP11 Assay of Bacterial Endotoxins in Radiopharmaceuticals by Microplate Reader

### H. Kvaternik, E. Plhak, B. Rumpf, D. Hausberger, RM. Aigner

#### Division of Nuclear Medicine, Department of Radiology, Medical University of Graz, Austria

##### **Correspondence:** H. Kvaternik

**Aim:** Radiopharmaceutical preparations for parenteral use must comply with the test for bacterial endotoxins according to the European Pharmacopoeia [1], whereby the endotoxin test can be carried out after release for the in-vivo application. For this purpose dedicated endotoxin testing devices are commercially available. Some nuclear medicine sites additional perform bioanalytical tests using an optical microplate reader. We aimed to approve and validate a method for endotoxin testing of radiopharmaceuticals with analysis and evaluation by a microplate reader.

**Methods:** For endotoxin testing the quantitative kinetic chromogenic LAL assay Kinetic-QCL, (Lonza Group Ltd, Basel, Switzerland) with a 96 well pyrogen-free microplate was used. The dynamic measurements were performed with a FLUOstar OPTIMA multi-detection reader (BMG Labtech, Ortenberg, Germany) [2]. The specific analysis was programmed with the operating software MARS. The test measures the reaction time (y) to reach the predetermined absorbance (threshold of 0.2 OD). 100 μl of standards (0.005, 0.05, 0.5, 5.0 EU/ml) and unknowns were measured at 405 nm in duplicate for a total of 100 min. The endotoxin concentrations (x) were calculated by linear regression fit with the formula log(y)=a*log(x)+b. As assay control, spiked samples (0.5 EU/ml) of the unknowns were measured in duplicate. To validate interfering factors, 6 samples of unknowns (^68^Ga-peptides in PBS matrix, 5 % EtOH) were diluted 1:10; 1:20; 1:50 and 1:100 and measured without/with a spike in duplicate.

**Results** In 3 validation runs accuracy, reparability, precision, and linearity were studied. All samples in the studies were valid with a spike recovery between 75% and 100%. The correlation of the calibration fit was >0.997 and LLOQ was found to be 0.005 EU/ml. The solvent matrix of ^68^Ga-peptides showed no interferences at a dilution of 1:10. Considering this dilution, the range of the endotoxins test stretched from 0.05 to 50 EU/ml. According to Ph. Eur. the limit for bacterial endotoxins in ^68^Ga-peptides solution for injection is defined with 175 EU per dose. Calculating 10 ml per dose the limit of 17.5 EU/ml is well covered.

**Conclusion:** We have demonstrated that an optical microplate reader is well suitable to test bacterial endotoxins in retain samples of ^68^Ga-peptides solutions. Up to 20 samples can be tested as bundle onto a 96 well microplate within 3 hours. After validation this analysis method was integrated successfully into the parametric release of our routinely prepared ^68^Ga-peptides.

**References**

1. Ph. Eur. 9.3, 2.6.14 (01/2018)

2. Quinlan C, Peters C. [2016] In: Application Notes Binder vol. 4. BMG LABTECH GmbH; AN237 p. 31-32. https://www.bmglabtech.com/fileadmin/06_Support/Download_Documents/Brochures/2016-ApplicationNotesBinderLR.pdf

## PP12 Ga-68 labeled quinazoline monomers and dimers bearing the HBED-CC chelator as PET tracers for EGFR-TK imaging

### C. Liolios^1^, A. Shegani^2^, I. Roupa^2^, C. Kiritsis^2^, A. Makarem^1^, M. Paravatou-Petsota^2^, M. Pelecanou^3^, P. Bouziotis^2^, M. Papadopoulos^2^, K. Kopka^1^, I. Pirmettis^2^

#### ^1^Division of Radiopharmaceutical Chemistry, German Cancer Research Center, Heidelberg, Germany; ^2^Institute of Nuclear and Radiological Sciences & Technology, Energy and Safety, National Center for Scientific Research “Demokritos”, Athens, Greece; ^3^Institute of Biosciences and Applications, National Center for Scientific Research “Demokritos”, Athens, Greece

##### **Correspondence:** C. Liolios

**Aim:** Epidermal growth factor (EGFR) receptor is over-expressed in several solid tumors. EGFR tyrosine kinase inhibitors (EGFR-TKI) bind to the intracellular TK domain and compete with adenosine triphosphate (ATP), preventing thus tumor cell proliferation, angiogenesis, and protection from apoptosis. In the present study, we aimed towards the synthesis of potential ^68^Ga labeled monomers and dimers of the EGFR-TKI pharmacophore (3-bromophenyl)quinazoline-4,6-diamine pharmacophore[1,2]. **1**, bearing the complexing agent HBED-CC (N,N′-bis[2-hydroxy-5-(carboxyethyl)benzyl]ethylenediamine-N,N′-diacetic acid). Lead structure **1** was also linked to a 4-aminobutanoic acid **2**. HBED-CC was conjugated to the aforementioned pharmacophores (**1** and **2**) *via* its functionalized propionic acid moieties, resulting in monomers **3** and **4** or dimers **5** and **6**, respectively. Ligands **3-6** were tested *in vitro* for TK inhibition, radiolabeled with ^68^Ga and tested *in vivo* for their biodistribution profile in mice bearing A431 (human epidermoid carcinoma cell line) tumors.

**Methods:** The cytotoxicity of **1-6** was determined by the MTT assay against A431 cells by treating them with varying concentrations of **1-6** for 72 h (10 nM-1000 μΜ)^1^. HBED-CC analogues **3-6** (1.0 nM in HEPES buffer 2.4 mM, 90-100 μL) were radiolabeled with [^68^Ga]Ga^3+^ eluate (40 μL, 40-50 MBq), 98°C, 10 min, and evaluated for radiolabeling efficiency *via* radio-RP-HPLC. Tracers [^68^Ga]**i** (**i = 3-6**), were investigated *in vivo* for their tumor accumulation and biodistribution in A431 tumor-bearing SCID mice at 5, 60 and 120 min post injection.

**Results** *In vitro* MTT assays for **3-6** showed IC_50_ values in the μM range. Monomer **3** (43.60 ± 7.49 μM) was significantly better than **4** (62.84 ± 6.34 μM) and **6** (68.80 ± 3.71 μM) and identical with dimer **5**. Radiolabeling of **3-6** resulted in a single radioactive product [^68^Ga]**i** (**i = 3-6**), while radiochemical yield ranged between 98-100%. Biodistribution experiments for [^68^Ga]**i** (**i = 3-6**) showed tumor (A431) uptake which ranged between 0.90-1.34 %ID/g at 5 min. Monomer **3** and dimer **6** showed a fast tumor clearance (0.49-0.34 %ID/g at 60 min p.i.), while for monomer **4** and dimer **5** tumor uptake remained rather constant after 60 min p.i. (0.73-0.70 %ID/g). All compounds showed extensive uptake in the intestines at 60 and 120 min p.i., ranging between 23.23-43.18 %ID/g. Among the ligands studied monomer **4** presented less off-target uptake in combination with the higher tumor accumulation.

**Conclusion:** Four novel quinazoline monomers and dimers based on the HBED-CC chelator (**3-6**) were synthesized, radiolabeled with ^68^Ga and evaluated as biomarkers for EGFR-TK imaging. Among the tracers studied [^68^Ga]**i** (**i = 3-6**), monomer **4** presented the best pharmacokinetic characteristics regarding tumor uptake and off-target accumulation. Tracer **4** is being considered for further evaluation as PET tracer for EGFR-TKI imaging.

**References**

1. Fernandes C, et al. [2008] Dalton Trans. 24: 3215–3225.

2. Bernard-Gauthier V; et al. [2015] Molecules, 20 (12): 22000–22027.

## PP13 Prostate-specific membrane antigen (PSMA) and gastrin-releasing peptide receptor (GRPr) PET-imaging for prostate and breast cancer; tumor models and interactions with clinical relevance

### C. Liolios^1^, C. Patsis^2,3^, M. Roscher^1^, U. Bauder-Wüst^1^, M. Eder^4^, K. Kopka^1^

#### ^1^Division of Radiopharmaceutical Chemistry, German Cancer Research Center (DKFZ), Heidelberg, Germany; ^2^Cell Plasticity and Epigenetic Remodeling, German Cancer Research Center (DKFZ), Heidelberg, Germany; ^3^Department of Translational Oncology, German Cancer Research Center (DKFZ), Heidelberg, Germany; ^4^Division of Radiopharmaceutical Development, German Cancer Consortium (DKTK), Freiburg, Germany

##### **Correspondence:** C. Liolios

**Aim:** PSMA and GRPr are both highly expressed on prostate cancer (PCa), and thus considered attractive targets for imaging and therapy. GRPr has been also been suggested for imaging breast cancer (BC), while on the contrary for PSMA a limited number of positive cases has been reported. We compared two ligands linked to the chelator N,N’-bis[2-hydroxy-5-(carboxyethyl)benzyl]ethylenediamine-N,N’-diacetic acid (HBED-CC), H_2_N-(Ahx)-Lys-NH-CO-NH-Glu (PSMA-11) [^68^Ga]**1** and a GRPr antagonist, H_2_N-4-amino-1-carboxymethyl piperidine-[(R)-Phe^6^, Sta^13^]BN(6–14) [^68^Ga]**2**^1,2^. Both tracers were tested *in vitro* in PC-3, LNCaP (both PCa), T47D, MDA-MB-231(both BC) and in the *PSMA*-transduced and *PSMA*-overexpressing cell lines PC-3^+^, T47D^+^ and MDA-MB-231^+^. In addition, a GRPr probe for fluorescence-based flow cytometry (FACS), **3**, was synthesized by the replacement of HBED-CC with Alkyne-BDP-FL (Jena Bioscience®).

**Methods:** PSMA overexpression in PC-3, T47D and MDA-MB-231 cell lines was accomplished by means of lentiviral transduction and was subsequently confirmed by western blot in the respective cell lysates. **1** and **2** were synthesized and radiolabeled with ^68^Ga according to published methods^3^. Total cell bound [^68^Ga]**1** and [^68^Ga]**2** over time (0-90 min) was determined according to previously published methods^1,2^. FACS analysis with **3** was also performed on the following cell lines T47D^+^ (PSMA^+^), T47D^Mock^ (vector only), T47D (wild type).

**Results:** Maximum cell binding of [^68^Ga]**1** and [^68^Ga]**2** (PSMA- and GRPr-specific respectively) was achieved after 45 min of incubation time. Cell binding of [^68^Ga]**1** and [^68^Ga]**2** was increased after *PSMA* transduction in all cell lines tested after 1h incubation. More specifically, (results are expressed as *times*x above control, % given radioactivity) [^68^Ga]**1** (PSMA) was higher for MDA-MB-231^+^ (*100*x, ~80 %), T47D^+^ (*4*x, ~2.5 %), PC-3^+^ (*10*x, ~13 %) than controls (Mock or blocked with addition of 10.000-fold excess pharmacophore), while for LNCaP it was ~66 %. Interestingly, alongside PSMA overexpression caused by the transduction the binding of the GRPr ligand was also increased, thus [^68^Ga]**2** was higher for MDA-MB-231^+^ (*2*x, ~3 %), T47D^+^ (*2*x, ~9 %), PC-3^+^ (*2*x, ~30 %) than controls (Mock or blocked with addition of 1000-fold excess pharmacophore). These observations have been also confirmed by FACS using **3** in T47D and T47D^+^.

**Conclusion:** The transduction of PSMA positively affected the amount of cell bound ligand for both [^68^Ga]**1** (PSMA) and [^68^Ga]**2** (GRPr) in all PCa and BC cell lines studied. These results indicate a possible connection between the two receptors (PSMA/GRPr); further studies are underway to prove this hypothesis. The findings of this study might be of high impact for the development of novel radiopharmaceutical treatment strategies addressing tumor heterogeneity.

**References**

(1). Eder M, et al. [2008], Eur. J. Nucl. Med. Mol. Imaging 35 (10): 1878–1886.

(2). Liolios C, et al. [2016], EANM Barcelona (EPW84): S238–S238.

(3). Liolios C; et al. [2016], Bioconjug. Chem. 27 (3): 737–751.

## PP14 Radiolabeling of single domain antibodies with Tc-99m: evaluation of the best parameters for complexation allowing to preserve 3D conformation

### S. Bacot, M. Ahmadi, M. Debiossat, C. Ghezzi and A. Broisat

#### Univ. Grenoble Alpes, Inserm U1039, LRB, 38000 Grenoble, France

##### **Correspondence:** S. Bacot

**Aim:** Camelid single-domain antibodies (sdAb), also called VHHs, constitute a promising “new” class of imaging agent for nuclear medicine. When using the tricarbonyl method for radiolabelling VHH with Tc-99m, the optimal conditions must be specifically determined 1) by determining the parameters such as temperature and heating times to obtain the maximum of complexation with 99mTc but also 2) by evaluating the effects of temperature on their secondary structure.

**Methods:** Five VHH (VHH-r1, VHH-r2, VHH-r3, VHH-r4 and VHH-r5) were radiolabeled with technetium-99m using tricarbonyl method at their C-terminal hexahistidine-tag (His-Tag). For each compound, various parameters such as specific activity, time and temperature of incubation were evaluated to determine the ones allowing for obtaining the most elevated complexation with 99mTc. The radiochemical purity (RCP) was determined by HPLC before and after purification step with a NAP-5 size exclusion column. Circular dichroism (CD) spectra were achieved from 205 to 260 nm at temperatures ranging from 25°C to 85°C.

**Results** The conditions enabling to acquire the highest complexation with Tc-99m were found to depend on the evaluated VHH. For instance, they were of 45 min at 75°C for VHH-r1, VHH-r4 and VHH-r5, of 60 min at 60°C for VHH-r2, and of 90 min at only 40°C for VHH- r3. Similarly, conformational changes observed by CD were found to vary between the evaluated VHHs, with maximal thermal stabilities ranging from 40°C to 75°C. As a result, for 3 VHHs out of 5, the incubation temperature had to be reduced to achieve the maximum of complexation in order to preserve their 3D conformation (50°C for 90min for VHH-r2 and 60°C for 60min for VHH-r4 and VHH-r5). Once successfully radiolabeled and purified (PRC>95°C), we showed that all 99mTc-VHHs had preserved affinity for their target, except for 99mTc-VHH-r2.

**Conclusion:** This study underlines the fact that despite their structural similarities, each VHH responds differently to the conditions of radiolabeling with Tc-99m. Therefore, to radiolabel a VHH using 99mTc-tricarbonyl method it is very important to define optimal conditions of incubation in agreement with the maximum of complexation without affecting its 3D conformation.

## PP15 A comparison of four different dose calibrators using various isotopes and sample geometries

### I. Pooters, M. Bauwens, J. Wildberger, F. Mottaghy and R. Wierts

#### Department of Radiology and Nuclear Medicine, Maastricht University Medical Center +, Maastricht, The Netherlands

##### **Correspondence:** I. Pooters

**Aim:** Dose calibrators are medical devices used in nuclear medicine to determine the amount of radioactivity to be administered to patients. According to Dutch Nuclear Medicine Guidelines, dose calibrators should be validated for all radionuclides and geometries with a maximum deviation of 5% with respect to the true radioactivity. This validation requires the use of well calibrated radioactive standards or measurements with calibrated equipment. Because this is expensive and time consuming, validation is often limited to a comparison to other dose calibrators. It is questionable if this limitation is justified, especially when looking at the effect of the (type of) isotope, the dose calibrator type/manufacturer and the geometry of the radioactive sample.

**Methods:** In this study, we used and compared four different dose calibrators (ISOMED 2000, ISOMED 2010, Capintec CRC-25R, Veenstra VIK-202). In these, we measured activity from four commonly used isotopes (^18^F, ^68^Ga, ^99m^Tc and ^111^In) using different volumes in both syringes and vials. Additionally, we analyzed the effect of using different geometries (3 different brands of syringes and 4 different brands of vials) on the measurements in more detail on one system. All measurements were compared to a fully calibrated gamma spectroscopy semi-conductor system (GR1018, Canberra) to determine the deviation from the true radioactivity.

**Results** For ^18^F and ^99m^Tc, the deviations were mostly within the 5% limit on all devices and for all geometries. For ^68^Ga and ^111^In however, deviations of more than 30% from the true radioactivity and 50% between different dose calibrators were observed. Within one type of dose calibrator, the deviations are small for all isotopes: the standard deviation for repeated measurements is below 3%, while the effect of varying syringes and vials is about 5-10%.

**Conclusion:** Even though all dose calibrators showed acceptable deviations for ^18^F and ^99m^Tc measurements, the deviations for ^68^Ga and ^111^In are not conform legal regulations and require on-site modifications of the dose calibrator settings. Although the effect of sample geometry was generally limited (compared to the differences between isotopes), it frequently still exceeded the 5% limit as posed in the guidelines. We therefore advise to validate dose calibrators (at least one per type) for every isotope and for every geometry, using a well-calibrated standard. As this may affect the ‘medical device’ status of the system, discussion with the manufacturer is required.

## PP16 A first synthesis of ^11^C-labelled analog of 4’-O-methylhonokiol as a potential PET radiotracer for inflammation

### M.M. Kiseleva^1^, O.F. Kuznetsova^2^, N.B. Viktorov^3^, K.V. Sivak^4^, A.G. Alexandrov^4^, D.D. Vaulina^2^, N.A. Gomzina^2^

#### ^1^Saint Petersburg State University, WHO National Influenza Centre of Russia, Saint-Petersburg; ^2^N.P.Beсhtereva Institute of the Human Brain, Russian Academy of Sciences, WHO National Influenza Centre of Russia, Saint-Petersburg; ^3^Saint Petersburg State Institute of Technology (Technical University) , WHO National Influenza Centre of Russia, Saint-Petersburg; ^4^Research Institute of Influenza, WHO National Influenza Centre of Russia, Saint-Petersburg

##### **Correspondence:** M.M. Kiseleva

**Aim:** Neolignan 4’-O-methylhonokiol (MH) isolated from *Magnolia grandiflora* is known to have several biological activities, including anti-cancer, anti-inflammatory and anti-neurodegenerative effects [1]. MH has recently been shown to inhibit cyclooxygenase (COX) activity with a higher selectivity for COX-2 (IC_50_=0.062 μM) over COX-1 (IC_50_=2.4 μM) [2]. The development of COX-2 inhibitor radiolabeled analogs is of special interest for PET visualization of inflammation and tumors. Therefore we suggested a MH analog labeled with carbon-11 (4’-[^11^C]methoxy-5-propyl-1,1’-biphenyl-2-ol or [^11^C]MPbP) as a potential PET radiotracer. Unlabeled MPbP has demonstrated a high anti-inflammatory activity comparable to that of specific COX-2 inhibitor celecoxib in the preliminary tests on mice with a carrageenan-induced inflammation. The present study describes the radiolabeling procedure of [^11^C]MPbP.

**Methods:**

**Fig. 1 (abstract PP16).**
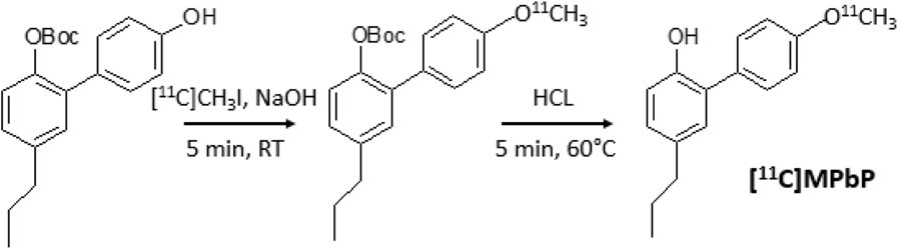
See text for description

The synthesis of [^11^C]MPbP was performed by two-step procedure starting from precursor with Boc-protecting group and operated with home-made fully automated module. The radionuclide carbon-11 (T_1/2_ = 20.4 min) in the form of [^11^C]CO_2_ was produced by a nuclear reaction ^14^N(p, α)^11^C in a PETtrace cyclotron (GE Healthcare). [^11^C]CH_3_I was produced using ‘wet’ method (LiAlH_4_/HI) and transferred under nitrogen flow (~10 ml/min) into reaction vessel with the precursor solution (2 mg in 1.2 ml of acetone and 0.7 М NaOH in water-ethanol solution (1:1, v/v) as a base). [^11^C]-methylation reaction was performed at room temperature in 5 min. The hydrolysis step was accomplished at 60°C with 12 M HCl in 5 min. The efficiency of O-^11^C-methylation as well as yield of [^11^C]MPbP were determined in crude reaction mixture using radio-TLC on Silicagel 60 F_254_ UV-plates with hexane/ethyl acetate (4/1) as a mobile phase and radio-HPLC (Waters X-Bridge column 4,6x150 mm, MeCN/H_2_O (60/40) as eluent with flow-1.3 ml/min). The radiochemical identity of [^11^C]MPbP was evaluated by comparison with the reference compound.

**Results** For the first time, [^11^C]MPbP, a novel potential COX-2 inhibitor was produced. The ^11^C-methylation yield was about 60% and [^11^C]MPbP was resulted in 45% yield based on [^11^C]CH_3_I.

Work is now in progress to further optimize synthesis and purification of [^11^C]MPbP and to investigate its biochemical characteristics (plasma stability, lipophilicity, BBB permeability, binding affinity to COX-2). This study was funded by RFBR according to the research project 17-04-02119 A.

**References**

1. Kumar A, Singh UK, Chaudhary A, [2013], Future Med. Chem. 5(7): 809-829

2. Kim HS et.al., [2015], Arch. Pharm. Res. 38(5):813-825

## PP17 Synthesis of WX-360-derived, uPAR-binding PET Tracers and Development of a Platform to Evaluate Their *in Vitro* Binding Affinities

### A. Wacker^1,2^, U. Bauder-Wüst^1^, M. Schäfer^1^, J. Schmidt^1^, A. Kopp-Schneider^3^, C. Liolios^1^, K. Kopka^1^

#### ^1^Division of Radiopharmaceutical Chemistry, German Cancer Research Center (DKFZ), Heidelberg, Germany; ^2^Faculty of Biosciences, University of Heidelberg, Heidelberg, Germany; ^3^Division of Biostatistics, German Cancer Research Center (DKFZ), Heidelberg, Germany

##### **Correspondence:** A. Wacker

**Aim:** Binding of the urokinase-type plasminogen activator (uPA) to the membrane anchored receptor uPAR is defined by its amino terminal fragment (ATF) consisting of a kringle and a growth factor-like domain (GFD). The latter is buried deep in the binding cavity and harbours the majority of the high-affinity interactions. A cyclic peptide (WX-360: *cyclo*^21,29^[D-Cys^21^]-uPA_21-30_[S21C;H29C]) was derived from this original structure demonstrating a capability to inhibit tumour growth and spread [1,2]. As the uPA system is frequently associated with the process of invasion/metastasis and a more aggressive phenotype in a great variety of cancers, there is a great interest in PET imaging using uPAR-directed tracers to visualise the tumour’s potential for metastasis formation. Hence, this study uses the cyclic peptide WX-360 as a starting point for the development of new PET tracers.

**Methods:** Ligands were synthesised on solid phase. A competitive bioassay based on immobilised rhuPAR was developed to assess their binding affinities. Briefly, increasing concentrations of ligand (0-1000 μM) were incubated in the presence of a 0.1 nM solution of a monoclonal mouse IgG_1_ antibody (clone #62022, R&D Systems) directed against the uPA-binding pocket of uPAR. Binding of a horseradish peroxidase conjugated secondary goat anti-mouse IgG_1_ antibody and subsequent conversion of a chromogenic substrate generated the signal which was read at 450 nm. A competitive cell-based binding assay to re-evaluate the initial results is currently being developed.

**Results** Saturation and competitive binding studies employing the natural ligand uPA revealed specific binding of the primary mAb (K_d_ = 1.0 ± 0.1 nM). Assay specific IC_50_ values of the peptide-based, literature-known structures DOTA-AE105 and WX-360 were in the low micromolar range while no binding was detected for DOTA-AE105M (non-binding mutant) [3,4]. Studies on the optimisation of the binding motif were promising for methylation of the central lysine residue and exchange of the disulphide bridge for a peptide bond. Conjugation of a DOTA chelator for radiometal complexation was accomplished by insertion of various linkers at the C-terminus of WX-360, the resulting compounds displaying similar inhibitory potentials as compared to the original structure. Placing the chelator at the N-terminus, however, slightly reduced the binding affinity.

**Conclusion:** With initial positive results, this study presents the potential of deriving uPAR-binding PET tracers from the peptide WX-360. The platform for compound characterisation is currently expanded to re-evaluation of IC_50_ values in a cell-based assay as well as determination of logP values and serum stabilities.

**References**

1. Schmiedeberg N, Schmitt M, Rölz C, Truffault V, Sukopp M, Bürgle M, Wilhelm OG, Schmalix W, Magdolen V, Kessler H, [2002], J. Med. Chem. 45: 4984-94.

2. Sato S, Kopitz C, Schmalix WA, Muehlenweg B, Kessler H, Schmitt M, Krüger A, Magdolen V, [2002], FEBS Lett. 528: 212-6.

3. Persson M, Madsen J, Østergaard S, Jensen MM, Jørgensen JT, Juhl K, Lehmann C, Ploug M, Kjaer A, [2012], J. Nucl. Med. 53: 138-45.

4. Persson M, Skovgaard D, Brandt-Larsen M, Christensen C, Madsen J, Nielsen CH, Thurison T, Klausen TL, Holm S, Loft A, Berthelsen AK, Ploug M, Pappot H, Brasso K, Kroman N, Højgaard L, Kjaer A, [2015], Theranostics 5: 1303-16.

## PP18 Fully-automated production of 2′-[^18^F]fluoroflumazenil without using gradient HPLC purification

### F. Trejo-Ballado, E. Zamora-Romo, H. Gama-Romero, J.C. Manrique-Arias, G. Contreras-Castañon, R. Tecuapetla-Chantes, A. Zarate-Morales, A. Flores-Moreno, M.A. Avila-Rodriguez

#### Unidad Radiofarmacia-Ciclotrón, Facultad de Medicina, Universidad Nacional Autónoma de México, Cd.Mx., 04510, Mexico

##### **Correspondence:** F. Trejo-Ballado

**Aim:** 2′-[^18^F]fluoroflumazenil (FFMZ) has proven useful for benzodiazepine receptor imaging. Our gold was to implement the fully-automated synthesis of [^18^F]FFMZ in a Tracerlab FXFN module.

**Methods:** Synthesis was performed via nucleophilic substitution using the chemical precursor Tosyloxyethylflumazenil (TFMZ), adapting the method reported by Yoon et al. [1] but avoiding the use of gradient HPLC. Briefly, [^18^F]Fluoride ion was trapped in a QMA cartridge and eluted with 1.5 mL solution of K.2.2.2. (5.3 mg/mL)/ K_2_CO_3_ (1.4 mg/mL) in MeCN/H2O (95/5). After drying of the azeotrophic mixture, 5 mg of TFMZ in 1.7 mL MeCN/0.3 mL DMSO were added to the reaction vessel and heated at 110°C for 12 min under intermittent purging with helium. The reaction mixture was diluted with 2 mL (×2) of water and passed through an activated Alumina cartridge to the HPLC-injection vial. Solution was then loaded onto the HPLC-loop for purification in a semi-preparative C18 reverse phase column (250/10 mm, Nucleosil 100-7 C18, Macherey-Nagel). Purification was performed under isocratic elution using two mobile phases at a flow rate of 3 mL/min: first with 100% water for 10 min then switching to 40% MeCN in water. The collected product-fraction was diluted with water (1:5) and loaded onto a preconditioned C18 cartridge. After rinsing with 3 mL of water, [^18^F]FFMZ was eluted with 1 mL of 70% EtOH, diluted with 10 mL of physiological saline, and sterilized by filtration (0.22 μm Millex-FG).

**Results:** [^18^F]FFMZ was eluted at 20 min after injection as shown in Fig. 1. Synthesis was satisfactorily accomplished yielding the final product in radiochemical yields of 20–25% decay corrected at the end of bombardment after a synthesis time of 60 min (*n* = 5), with a radiochemical purity >99% as determined by analytical HPLC.

**Conclusion:** A convenient and reliable synthesis of [^18^F]FFMZ was successfully adapted in an automated module by using two successive isocratic elutions instead of gradient HPLC. Final product showed no radioactive or non-radioactive impurities obtaining [^18^F]-FFMZ in enough quantity and quality for clinical applications.

**Reference**

1. Yoon YH, et al (2003) Nucl Med Biol 30:521-527.

**Fig. 1 (abstract PP18).**
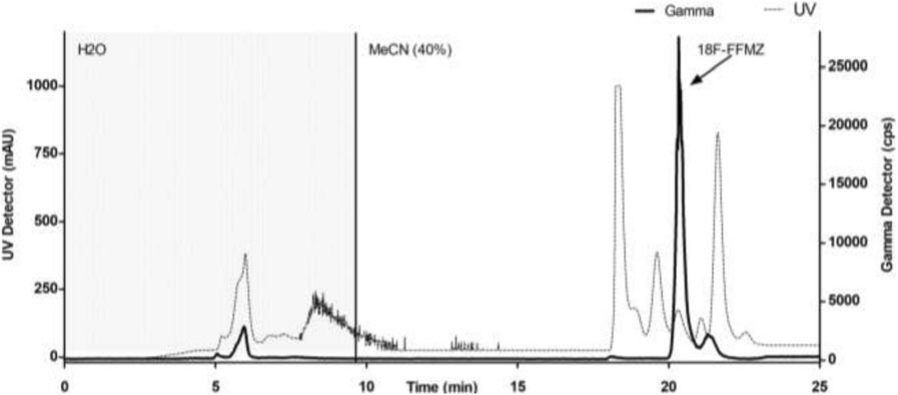
Full HPLC chromatogram of the purification of [^18^F]FFMZ

## PP19 Radioiodination of Small Molecules and Short Peptides; the Effect of Oxidant Reagents Choice on the Radiochemical Yields

### M. Al-Qahtani, H. Mutwali and Y. Al-Malki

#### Cyclotron & Radiopharmaceuticals Department, King Faisal Specialist Hospital and Research Center-Riyadh, Saudi Arabia

##### **Correspondence:** M. Al-Qahtani

**Aim:** The radioiodinated products are used as diagnostic or therapeutic agents. The diversity of the labelling methodologies that used to introduce the radioactive iodine into the target molecule are wide enough to make the selection of a specific route is of great interest. Several oxidant agents were used in labelling biomolecules. Three famous ones are Chloramine-T, Iodogen and Iodo-beads (the solid state of the Ch-T). However, the mentioned agents are producing varies radiochemical yields and more importantly stabilities of the produced labelling product always questioned. The current report is detailing the findings out of a study that was aimed in investigating several reaction factors on the above labelling agents when used with small molecules and short peptides.

**Methods:** The direct electrophilic method via Chloramine-T or similar oxidant was used. Following the end of the reaction the purification step was done using Sep-Pak C-18 Classic cartages that should be activated with 3 ml water prior the purification. Then the Sep-Pak was then washed with 1 ml water, then collect the product (labeled peptide) in the second wash which done by eluting with 0.5 ml MeCN and 1.5 ml mix of the HPLC system. The radioiodinated product was isolated by Sep-Pak and confirmed with the TLC and HPLC analysis methods.

**Results**

**Fig. 1 (abstract PP19).**
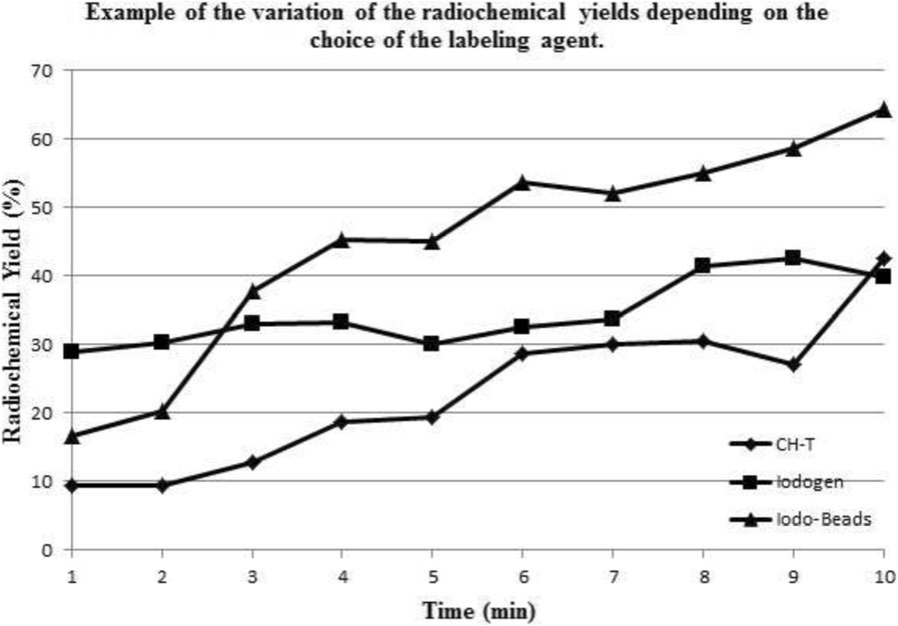
See text for description

Chloramine-T gives constant RCY most of the time under all conditions except at basic PH (8.5) in which heat is required. Iodogen gives better RCY when heated and higher PH 7.4 and 8.5 were used. The effect of the heat was clear at PH 6.5 as the RCY was nine times higher at 60°C. Also Iodo-beads gives higher RCY than the other two labeling agents except at PH 8.5 and 60°C, it was about 25 % (±5) less.

**Conclusion:** We can conclude that RCY and purity are very dependent on the right choice of the labeling agent and reaction conditions. However Iodo-beads give higher RCY than the other two labeling agents which make it the targeted intermediate oxidant reagents.

**Acknowledgements**

This work was supported by King Abdualaziz City for Science & Technology (AT-29-15), RAC # 2080 047.

## PP20 The Use of Nano-Sized Particles in Labelling New Class of Radiopharmaceuticals

### M. Al-Qahtani^1^, Y. Al-Malki^1^, H. Mutwali^1^ and M.E. Abouzied^2^

#### ^1^Cyclotron & Radiopharmaceuticals Department, King Faisal Specialist Hospital and Research Center-Riyadh, Saudi Arabia; ^2^Radiology Department, King Faisal Specialist Hospital and Research Center-Riyadh, Saudi Arabia

##### **Correspondence:** M. Al-Qahtani

**Aim:** Radiosynthesis an imaging radiotracer for a carefully selected biological process and choosing the right imaging modality become the top aims for all biomedical researchers. Positron Emission Tomography (PET) is the first on the list used for such investigations as it fulfils all informations required for imaging of biological process. On the other hand human cancer cells over express many peptide receptors which can be used as molecular targets. Therefore, radiolabeled receptor-binding peptides have emerged as an important class of radiopharmaceuticals for tumor diagnosis and therapy. As small peptides for receptor imaging and targeted radiotherapy have advantages over proteins, antibodies and antibody fragments as they are small and show rapid diffusion in target tissue and they have low molecular weight which will result in rapid clearance from blood and non-target tissues and that in turn will result in high tumor-to-background ratios.

**Methods:** In a routine process a 0.1mg of nanoparticles that been attached to a biological molecule (peptide) dissolved in 50μl H_2_O. ^68^GaCl_3_ eluent from a ^68^Ge/^68^Ga generator is used as the generator is fractionally eluted beginning with 5.0 ml of sterile 0.1N HCl. For fractional elution, the first 1.6 ml of eluate always discarded, the next 2.0 ml is collected for use and the final 1.4 ml also discarded. 200 μl of the fractionated Ga-68 eluate (approximately 1 mCi/37 MBq) is added to nanoparticle solution then 100μl of sodium acetate buffer (1.25M) or ammonium acetate buffer (0.25M) is added and allowed to react at (85-90°C) for 30 minutes, the pH of the reaction mixture should be kept at 5.5. Following incubation, the labeled nanoparticle is then tested for radiochemical yield and purity using Radio-TLC and HPLC.

**Results:** The labelled nanopatical attached to the biological molecule is monitered by both TLC and HPLC.

**Fig. 1 (abstract PP20).**
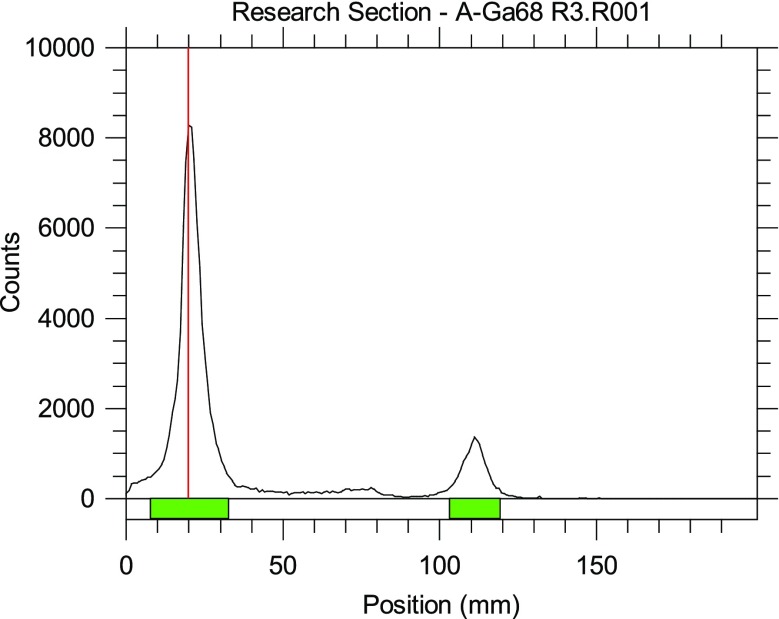
See text for description

Radio-TLC example of labelling reaction mixture; ^68^Ga-anoparticle remains at the origin with retention factor of 0.00, the purity approximately 85%, and the free ^68^Ga migrating to the solvent front with retention factor of 0.96.

**Conclusion:** New efficient methodology to label nanoparticles with Ga-68 was developed in order to promote the development of PET-nanoradiopharmaceuticals that in the future will overcome the limitation of the regular PET-radiopharmaceuticals in use nowadays.

## PP21 Biological Investigations of a laminin Class Peptide that has a potential as a Diagnostic and Therapeutic Properties

### Y. Al-Malki, and M. Al-Qahtani

#### Cyclotron & Radiopharmaceuticals Department, King Faisal Specialist Hospital and Research Center-Riyadh, Saudi Arabia

##### **Correspondence:** M. Al-Qahtani

**Aim:** Melanoma is a tumor of constantly accumulative occurrence for which novel methods of imaging and targeted therapy are generally pursued. Developing a newly laminin class peptide that been labeled with position emitter Ga-68 or I -124 and with SPECT emitter I-131 will be discuss. In order to evaluate the potential relevance of positron emitter labeled a laminin class short peptide with enhanced melanoma targeting capacity, the uptake of ^68^Ga/^124^I-peptide were investigated in SK-MEL 28 melanoma cell line. Also the same peptide was labelled with ^131^I to be tested as therapeutic approach.

**Methods:** High performance liquid chromatography (HPLC) system and Thin Layer Chromatography (TLC) were used for quality control purposes. The radiochemical purity were > 99.00% (*n* = 5). The stability was persistent over 6 h and amounted to > 98.55% ±0.35% (*n* = 15) for the ^68^Ga-peptide. In vitro receptor binding was performed on the mentioed cell line and biological evaluation was done in normal and mice bearing SK-MEL28 cell xenograft. Animal PET/CT using ^68^Ga-peptide and ^124^IGa-peptide were performed in murine models of melanoma.

**Results:** As determined by HPLC, the ^68^Ga-short peptide efficiency was >70% and radiochemical purities always > 99% in short reaction time. These synthetic approaches hold substantial promise as a rapid and efficient method amenable for automation for the labelled of peptides with high radiochemical yield and short synthesis time. For radioiodination direct electrophilic method were used with High RCY.

**Conclusion:** The uptake of ^68^Ga-peptide was high by pheomelanotic SK-MEL-28 human melanoma cells. In vivo characterization in normal mice revealed rapid blood clearance of ^68^Ga-peptide with excretion by both urinary and hepatobiliary pathways. In vivo imaging using animal PET/CT is confirming the later findings yielded a high tumor-to-background ratio at 1 h and at 2 h. In vitro tests have shown that significant amount of the ^68^Ga-peptide associated with melanoma cell fractions. Due to its easy handling and quite high uptake by melanoma cells, we expect that this peptide could be successfully used in routine application for melanoma imaging or eventual radiotherapy suggesting great potential for noninvasive clinical evaluation of suspected metastatic melanoma.

**Acknowledgements**

This work was supported by King Abdualaziz City for Science & Technology (AT-29-15), RAC # 2080 047.

## PP22 Improvement of iodide and iodate identification method in the radiochemical analysis of Iodine-131 radiopharmaceutical

### C. Y. Furukawa, V. Mendonça, N. T. O. Fukumori and M.M. N. Matsuda

#### Radiopharmacy Centre, Nuclear Energy and Research Institute, IPEN-CNEN/SP, Brazil

##### **Correspondence:** N. T. O. Fukumori

**Aim:** Sodium Iodide (131 I) is a radiopharmaceutical available as oral capsule or solution, and is largely used in nuclear medicine for tyroid scintigraphy imaging and in radioiodine therapy. Radiochemical purity (% RqP) is defined as the percent of total radioactivity in the desired chemical form. The main methods to determine % RqP is paper chromatography (PC), thin-layer chromatography (TLC) and high perfomance liquid chromatography (HPLC). Some compendial methods also establish procedures for retardation factor (R_f_) determination^1-2^. The objective of this work was to evaluate the mobile phase effect on the R_f_ of iodide (I^-^) and iodate (IO_3_^-^) in the % RqP analysis of Sodium Iodide (131 I).

**Methods:** Whatman 3MM PC (1.5 × 12.5 cm), methanol and glacial acetic acid were from Merck Millipore. Methanol and purified water in the proportion of 50%, 70%, 75% and 85% (v/v) were the mobile phases. Diluent solution containing 0.1 mg/mL KIO_3_, 0.2 mg/mL KI and 1.0 g/mL Na_2_CO_3_, and standard solutions of 0.2 g/mL KI and 0.4 g/mL KIO_3_ were prepared. In the origin position of a 10 cm paper strip, 5 μL of the diluent solution were applied. After chromatographic separation in each mobile phase, the suitable standard solution was dripped on the iodate and iodide expected positions followed by drops of glacial acetic acid to reveal the R_f_ of the species with formation of a brown spot^3^.

**Results:** The American and Argentine pharmacopoeial methods present iodate-131 as the main radiochemical impurity but differ in the mobile phase composition and in the color development method for iodide and/or iodate^1-2^. The systems advocate the use of 30 and 20 cm long stationary phases which require several hours for separation of the species, and methanol 70% and 75% (v/v), respectively. In a 10 cm strip and 70% (v/v) methanol, the R_f_ for iodate was 0.5-0.7 and for iodide was 0.7-0.9; with 85% (v/v) methanol, the R_f_ were 0.3-0.5 for iodate and 0.7-1.0 for iodide, respectively, taking an hour and a half for the chromatografic run. The resolution obtained with 85% (v/v) methanol was better when compared to methanol 70% (v/v).

**Conclusion:** By the use of the staining test for iodate and iodide proposed by the Argentine pharmacopoeia, it was possible to improve the % RqP analysis of Sodium Iodide (131 I), decreasing the analysis time of more than 4 hours to an hour and a half.

**References**

1. United States Pharmacopeia. 39 Ed. Rockville: United States Pharmacopeia Convention, 2017.

2. Farmacopeia Argentina http://www.anmat.gov.ar/fna/septima_edicion.htm

3. Ohlweiler OA, Química Analítica Quantitativa, Vol. 2, 3. Ed., LTC, RJ, 1982.

## PP23 Delivery of DTPA through liposomes as a good strategy for enhancing plutonium decorporation

### M. Mougin-Degraef^*1*^, F. Lelan^*1,2*^, C. Maurel^*1*^, L. Navarro^*1*^, P. Le Saëc^*1*^, S. Bohand^*3*^, L. Miccoli^*2*^ and O. Grémy^*2*^

#### ^1^CRCINA, Inserm, CNRS, Université d’Angers, Université de Nantes, Nantes, France; ^2^Laboratoire de RadioToxicologie, CEA, Université de Paris-Saclay, Bruyères-le-Châtel, France; ^3^AREVA MINES, Courbevoie, France

##### **Correspondence:** M. Mougin-Degraef

**Aim:** Internal contamination with plutonium (Pu), either in the context of accidental occupational exposure or as a result of possible terrorist use, continues nowadays to be a potential hazard. The practical way to reduce Pu body burden and the associated radiation risks is decorporation by chelation therapy by injection of marketed Na_3_-Ca-DTPA solution. This study aimed at assessing the efficacy of 110nm-sized liposomes encapsulating the DTPA in plutonium-exposed rats. The comparative effects of liposomal and free DTPA at similar doses were examined in terms of limitation of alpha activity burden in rats receiving various treatment regimens.

**Methods:** Unilamellar liposomes (DSPC/Cholesterol/DSPE 69:30:1) were used to encapsulate DTPA (25mM). For decorporation experiments, rats were first contaminated by intravenous administration of the soluble citrate form of ^238^Pu (4 to 10.3 kBq), before injection of DTPA, marketed free form or encapsulated in liposomes (2.25 at 6.74 μmol/kg). Treatment schedules started at one hour (prompt treatment) or at seven days (delayed treatment) after contamination and were given as a single injection or repeated injections. A prophylactic single treatment was also tested given at three days before contamination. Organs of interest (liver, bone and spleen mainly) and excreta were collected at different times for measurement of Pu alpha activity.

**Results** Liposomal DTPA given at 1h post-contamination reduced significantly Pu retention by 2.5-, 1.6- and 3-fold in liver, bone and spleen respectively, compared with free DTPA. For delayed administration, liposome-entrapped DTPA decreased hepatic, skeletal and splenic Pu levels by, respectively 68, 39 and 74% of the levels in untreated control rats (only 19, 12 and 24% with a similar dose of free DTPA). In addition, repeated injections of liposomal DTPA improved the removal of Pu compared to single injection and the efficacy of the prophylactic treatment was also observed.

**Conclusion:** The advantage of liposomal DTPA was undoubtedly directly and indirectly due to the better cell penetration of DTPA when loaded within liposomes, mainly in the tissues of the mononuclear phagocytic system. The decorporation induced by liposomal DTPA may result firstly from intracellular chelation of Pu deposited in soft tissues, predominantly in the liver. Afterwards, the slow release of free DTPA molecules from these tissues may enable a sustained action of DTPA, probably mainly by extracellular chelation of Pu available on bone surfaces. To conclude, Pu decorporation can be significantly improved by liposomal encapsulation of DTPA regardless of the treatment regimen applied.

## PP24 Validation of a clean room for the production of radiopharmaceuticals at Turku PET Centre

### S. Forsback^1,2^, N. Laurén^1^, J. Bergman^1^, R. Kivelä^3^

#### ^1^Turku PET Centre, University of Turku, Turku, Finland; ^2^Department of Chemistry, University of Turku, Turku, Finland; ^3^Hospital Pharmacy, Turku University Hospital, Turku, Finland

##### **Correspondence:** S. Forsback

**Aim:** According the EU GMP Guide the radiopharmaceuticals should be manufactured in controlled areas and the premises maintained so that the level of particles and microbiological contamination are low. At Turku PET Centre (TPC) the production of radiopharmaceuticals is performed in EU GMP class C clean room. Thus validation of the clean room shall ensure that the area fulfills all EU GMP requirements for this classification.

**Methods:** In order to grade our production premises, we have compiled a validation plan which was accepted by the quality assurance person of TPC. The validation process according the accepted validation plan was performed by an outside contractor according to valid standards (methods, calibrations of the measurement equipment and documentation). The tests include measurements of HEPA filter integrity, air volume flow, room differential pressure, airflow visualization, air change rate, airborne particle levels and viable microbial particles. The microbiological testing and measurement of airborne particles were performed both at rest and in operation. For microbiological testing, active and passive air samples using settle plates and surface samples using contact plates were taken from spots specified in the validation plan.

**Results:** The contractor compiled a validation report including all measurement records. All the requirements were fulfilled, the report was checked and accepted by the responsible person at TPC.

**Conclusion:** To maintain the classification, following measurements are performed annually: HEPA filter integrity test, air volume flow, room differential pressure, room air change rate and airborne particle levels. In addition, the room differential pressures are monitored automatically all the time giving alarm when out of specification. Microbiological sampling and measurement of airborne particles are performed regularly according to a standard operation procedure. Microbiological sampling is done at least once a month. Airborne particles are measured at least once a week during end product sterile filtration. While doing aseptic work, microbiological sampling and the measurement of particles are always performed.

## PP25 Use of Gravity Perfusion Method in PRRT: Experimental Evaluation and Optimization

### I. Rotaru and M. Ben Reguiga

#### Radiopharmacy Unit - Hôpital Beaujon, 100 Bd du Général LECLERC, 92110 Clichy, France

##### **Correspondence:** M. Ben Reguiga

**Aim:** 177Lu-DOTATATE (Lutathera®) indicated in endocrine tumors PRRT should be administered according to its SPC by a slow intravenous infusion (7.4GBq whitin 30min-or-50mL/h) with either the “2-pump” or the “gravity” methods. This latter method consists in using a saline solution placed in height and connected to the Lutathera® vial by an infusion line, which flows the vial causing volume expansion and overpressure, pushing the diluted product into a second infusion line that goes to the patient. The first trials we made with this technique showed that it was as imprecise as difficult to control. The aim of this work was to optimize exprimentally this technique and compare to the other ones.

**Methods:** A saline bag(250mLw) as placed at 2m height, connected to an infusion line (set at 16drops/min -or-50mL/h) ending with a 22G/30mm-needle. The needle was inserted into the Lutethera vial placed at 1,5m height. A second long 19G/88mm-needle deeply inserted into the vial septum until touching its bottom -was connected to the venous perfusion line going to the patient. For radiation safety reasons, 177Lu-DOTATATE was substituted by a cold saline solution stained by Red Ponceau and placed a Lutathera empty vial sealed with a new rubber stopper and an alumunium ring. The evaluation of photometric absorption replaced the radioactive measurments. Once the assembly of this system set up, the infusion was started, 150 mL the colored solution was graudally collected at the line outlet in 5mL fractions which content was assessed by UV-visible-spectrophotometry at 550nm. Measurments permitted to evaluate 3 parameters: volumic activity of the perfusate (VAp), (in %, AT) and residual activity in the bottle (in %, AR). Results are expressed as mean±SD (*n* = 3).

**Results:** Collected perfusate shohwed a volumic activity wich decreased gradually in exponential way [Vap=A0.e(-0,062. Perfused-Volume);R2=0.99]. Similar evolution was found in the residual activity of the vial, which disappeared according to the same mode. Symmetrically, the total perfused activity gradually increased in an exponential mode. After 30 minutes of infusion (infusion time requested in the SPC), a significant amount of the product (21.0±1.1%) remained in the vial. We calculated that to administer 99% the total delivered activity, it was necessary to infuse a a total volume between 81mL and 86 mL. After 100mL of infusion, only 0.45±0.26% remained in the vial.

**Conclusion:** While the pump infusion techniques produce uniform and homogeneous perfusates, gravity technique was found to deliver a variable content perfusate making difficult the control of cumulative perfused activity, and perilous the planning administration of partial activities following dose adjustments. The infusion of the entire content requires at least 81mL of aline, significantly higher than the SPC requirements.

## PP26 Inetraction of 177Lu-DOTATATE with pharmaceutical Vehicles and Peptides

### I. Rotaru and M. Ben Reguiga

#### Radiopharmacy Unit - Hôpital Beaujon, 100 Bd du Général LECLERC, 92110 Clichy, France

##### **Correspondence:** M. Ben Reguiga

**Aim:** 177Lu-DOTATATE is indicated in neuroendocine tumor PRRT. Its therapeutic efficiency and safety are tightly related the maintenance during all the treatment cycle of the chelate stucture integrity until its internalisation in tumour cells. Any alteration on its structure may lead to the complex destruction or unmettalation. These reactions may be suspected during co-infusion of other pharmaceuticals impacting pH, such as alkaline vehicles, or in patient freshly pretreated with cold somatostatin analogues which may be compettive to the bound peptide. The aim of this work is to study chemical interactions of 177Lu-DOTATATE in co-infusion conditions with various pharmaceutical vehicles and non-radioactive somatostatin analogues.

**Methods:** 177Lu-DOTATATE (Lutathera^®^) was diluted to 1/5th in saline, 5% Glucose, Bicarbonate 8.4% solution, Ringer-Lactate, octreotide solution, octreotide LAR or incubated at 37°C in plasma or plasma enriched with 10% de octreotide. Radiochemical purity (RPC) was determined by ITLC-SG in 0.1mol/L sodium citrate pH=3. The labelled peptide had retention factor (Rf) of 0.1 (0.2 in plasma) and the free radionuclide migrates at Rf=1.0. RPC was also determined using C18 Sep-Pack-Plus cartridge (Waters, USA). The free radionuclide was eluted with 5mL of 0.1mol/L acetate buffer pH=3 and the labelled peptide with 5mL of methanol, and hydroxylated Lu-177 remained inside the cartridge. Mixtures were then analyzed immediately, 30min and 24h after dilution. Results were expressed as mean±SD (*n* = 3-4) and RPC was considered as compliant when it was >95%. Binary comparisons to the undiluted product were made by a Mann-and-Whitney *U* test and stability over time checked by a 1-way ANOVA (*p* < 5%).

**Results:** 177Lu-DOTATATE diluted in saline or glucose or with cold peptides showed compliant RPC levels during the 24hours study duration. In Bicarbonate and Ringer Lactate, significant amounts of unbound Lutetium were found during Sep-Pack extraction, respectively 10.5±3.3% and 8.9±4.2%. In plasma mixtures, significant amounts of either hydroxylated Lutetium and or unbound lutetium were found only within 24h incubation: respectively 4.9%±1.3% and 4.2%±0.6% in plasma and respectively 6.3%±1.8% 4.8%±0.8% in plasma enriched with octreotide.

**Conclusion:** Hydroxylated Lutetium and unbound lutetium found in plasma 24 hours post-incubation may be due to the catalytic activity of plasma which slowly deteriorates the lutated complex. This is unlikely to occur in vivo: Lutatate is rapidly cleared from the bloodstream within few hours post-infusion. According to our results, the co-infusion of 177Lu-DOTATATE with alkaline pharmaceutical vehicles should be avoided: it affects the complex stability and induces Lu-177 release.

## PP27 Physiological ^89^Zr-Oxalate Solution for PET Diagnosis: Purification, Formulation, Biological Evaluation

### V.B. Bubenschikov, M.V. Zhukova, A.S. Krasnopyorova, A.Ya. Maruk, A.A. Larenkov

#### Burnasyan Federal Medical Biophysical Center, Moscow, Russia

##### **Correspondence:** A.A. Larenkov

**Aim:** The positron-emitting ^89^Zr (T_1/2_=78.42h) is widely studied for PET. Monoclonal antibodies labeling is the main focus of these studies. But should we disregard the diagnostic potential of simple ^89^Zr compounds?! The possibility of effective diagnosis of inflammation, skeletal disorders and even tumors using ^89^Zr-oxalate and other complexes was previously demonstrated [1,2]. The aim of this study is to develop handy procedure for production of ^89^Zr-oxalate in physiologically acceptable form and evaluate its potential for PET diagnosis.

**Methods:** All chemicals and solvents were of high-purity or pharmaceutical grade and were purchased from Sigma-Aldrich or Panreac. Quality control was carried out with TLC chromatography (iTLC-SG – 50 mM DTPA solution and other eluents; PET-MiniGita radio-TLC scanner). Dowex 1, Chelex-100 (Sigma-Aldridge), ZR (Triskem), Chromafix HCO_3_ (Macherey-Nagel) resins were used. ^89^ZrCl_4_ in 5 M HCl was purchased from Cyclotron Ltd, Obninsk, Russian Federation. *In vivo* experiments were carried out in BALB/C mice models with different pathologies. Wizard 2480 gamma-counter and G4 microPET (Sofie) were used for biological evaluation.

**Results:** It was found that ZR resin allows to obtain ^89^Zr-oxalate solution with highest yield and lowest metal impurities content. Chromafix HCO_3_ and Chelex-100 resins allow to obtain high purity as well. However, ^89^Zr-oxalate solution purified with ZR resin does not meet all the requirement for the PET radiopharmaceutical under development. Its virtues and shortcomings will be presented in detail. Using Chelex-100 resin allowed to develop the procedure for production of pH-neutral isotonic ^89^Zr-oxalate solution with high purity and specific activity. Radiochemical yield of the preparation and purification procedure is ≥90%. Radiopharmaceutical radiochemical purity remains ≥95% for at least 2 weeks. ^89^Zr complexes with other carboxylic acids were studied as well. Biological data indicate that ^89^Zr complexes with carboxylic acids are promising visualizing agents for infection and inflammation foci and musculoskeletal injuries. Toxicity tests were carried out as well. Experimental results will be presented in detail.

**Conclusion:** Handy procedure of production of ^89^Zr-oxalate physiologically acceptable solution was developed. Its potential for PET diagnosis was evaluated.

**Ethics approval:** Animal experiments were carried out according to European Convention for the Protection of Vertebrate Animals used for Experimental and Other Scientific Purposes. The work was financially supported by Ministry of Education and Science of the Russian Federation – State Contract № 14.N08.11.0162.

**References**

1. Park J-A, Lee YJ, Lee JW et al. [2016] MolPharm13(7): 2571–2577.

2. Larenkov A, Maruk A, Zhukova M, Krasnopyorova A [2017] EJNMMI 44(Suppl2): S554.

## PP28 Investigation of elution of the SnO_2_-based ^68^Ge/^68^Ga generator with different hydrochloric acid concentrations

### DM Prince^1^, D Rossouw^1^, SM Rubow^2^

#### University of Groningen, University Medical Center Groningen, Department of Nuclear Medicine and Molecular Imaging, Hanzeplein 1, 9713 GZ Groningen, The Netherlands

##### **Correspondence:** DM Prince

**Aim:** A SnO_2_-based generator requires higher HCl eluent concentrations than other commercially available generators. The manufacturer of the iThemba LABS generator recommends 0.6 M HCl as eluent, but use of 1 M HCl has been reported. There is only limited published data on the characteristics of eluates after elution of a SnO_2_-based generator with eluent concentrations lower than 0.6 M HCl. The aim of this investigation was to compare elution efficiencies, ^68^Ge breakthrough and metal contaminants of ^68^Ga eluates using 0.2 to 1 M HCl eluents and to qualify the possible benefits over the risks of any particular eluent over the recommended 0.6 M HCl.

**Methods:** ^68^Ga was eluted from a 1 to 2-month old 1110 MBq SnO_2_-based ^68^Ge/^68^Ga generator up to thrice daily on weekdays. The generator was eluted at least 6 times in total with each concentration of HCl (0.6 M, 0.4 M, 0.2 M, 0.8 M and 1.0 M) with rinses of 10 ml between concentrations. Rinses were done using the eluent intended for the subsequent elution. A period of at least 5 hours was allowed between elutions. The generator was eluted with 0.5 ml fractions up to a volume of 10 ml to construct elution profiles for each eluent. Elution yield was calculated as percentage of nominal ^68^Ge activity in the generator. ^68^Ge breakthrough was determined in each sample. Metal contaminants (Zn, Fe, Cu, Ga) in eluates were determined by ICP-OES.

**Results** Elution yield of the generator increased from 18% with 0.2 M HCl to 126% with 1.0 M HCl. ^68^Ge breakthrough increased from 0.004% with 0.2 M HCl to 0.62% with 1.0 M HCl. Fe content was fairly consistent around 0.32 ppm; Zn increased with increasing acidity; and Ga content remained fairly constant up to 0.6 M HCl and then increased consistently. Cu was only detected in 1.0 M HCl eluates.

**Conclusion:** An increase in the HCl concentration of the eluent resulted in increasing generator elution yields but also higher ^68^Ge breakthrough and Zn content. ^68^Ge breakthrough could be problematic with regards to radiation safety and radioactive waste control. In order to compromise optimal elution yield with minimal ^68^Ge breakthrough, 0.6 M HCl should therefore be the eluent of choice for a SnO_2_-based generator.

## PP29 Radiolabeling Efficiency of Peptides that Interact with Overexpressed Receptors on Tumor Cells. Relevance to the Glioblastoma

### D.V. Sobral^1^, A.C.R. Durante^2^, A.C.C. Miranda^2^, F.R. Cabral^1^, C.R. Nakaie^3^, M.F.F. Barboza^2^, L. Malavolta L^1^

#### ^1^Department of Physiological Sciences, Santa Casa de Sao Paulo School of Medical Sciences, Sao Paulo, Brazil; ^2^Instituto Israelita de Pesquisa Albert Einstein, Sao Paulo, Brazil

##### **Correspondence:** L. Malavolta L

**Aim:** Radiolabeled peptides have become very important in nuclear medicine and oncology in recent years mainly because they represent the molecular basis for *in vivo* imaging and radiopharmaceutical therapy with high specificity and affinity for overexpressed receptors in tumors. The objective was to evaluate the radiolabeling efficiency of the ^131^I-peptides, as well as their interaction with overexpressed receptors on tumor cells related to glioblastoma.

**Methods:** The EEEEYFELV peptide and its analogue DEDEYFELV both with affinity for EGFR receptor and the fragment GRGDYV with high affinity for the integrin receptor were synthesized accordingly to the Fmoc protocol and purified by preparative HPLC. All the peptides were radiolabeled with the radioisotope ^131^I-Na. The radioiodination was evaluated and optimized using the methodology of Chloramine-T. Radiochemical yield analyses of ^131^I-EEEEYFELV and ^131^I-DEDEYFELV peptides were performed by Whatmann 3MM using MeOH 95% and by thin layer chromatography on silica gel TLC-SG (Al) in ACN 95% for ^131^I-GRGDYV peptide. The stability studies *in vitro* were realized at 2, 24, 48 and 72 hours in room temperature and refrigerate and in human serum at 37°C up to 24 hours. Partition coefficient and binding to plasma proteins were determinate for all radiopeptides. The interaction of radiolabeled peptides with tumorigenic cells was assessed by using culture cells (C6) and brain homogenate of the glioblastoma animal models (*n* = 6).

**Results:** The peptides EEEEYFELV, DEDEYFELV and GRGDYV were efficiently synthesized, radiolabeled and showed radiochemical yield of 90.32% ± 0.30, 94.67% ± 0.27 and 97.95% ± 0.05 (*n* = 7), respectively. The stability studies showed that all the peptides were stable within 72 hours when stored in the refrigerator and up to 24 hours in human serum showing an average of radiochemical purity of 91.65% ± 1.33 (*n* = 3) for all peptides. All ^131^I-peptides have hydrophilic features and showed a binding percentage to plasma proteins of 59.47% ± 0.74, 57.68% ± 1.29 and 34.92% ± 2.53 (*n* = 3). Furthermore, the peptides presented an interaction affinity with the tumorigenic cells of 4.48% ± 0.22; 4.40% ± 0.30 and 1.73% ± 0.38, respectively and a internalization of approximately 45% for all fragments.

**Conclusion:** The peptides were efficiently synthesized and the tested radiolabeling strategies showed successful results. Moreover, all the peptides demonstrated affinity for the tumor cells evaluated. These results obtained in this study are consistent to adapt in the clinical application.

**Funding:** FAPESP, CAPES and FCMSCSP (FAP).

## PP30 An automated synthesis of Ga-68 labelled ubiquicidine

### J.S. Le Roux^1^, S.M. Rubow^1^, T. Ebenhan^2^, C.C.P. Wagener^3^

#### ^1^Stellenbosch University, South Africa; ^2^University of Pretoria, South Africa; ^3^South African Nuclear Energy Corporation, South Africa

##### **Correspondence:** S.M. Rubow

**Aim:** Ubiquicidine (UBI) 29-41 is currently being investigated as a potential infection imaging agent. All published methods for radiolabelling of this tracer to date describe manual processes. The current manual method for labelling UBI 29-41 with Ga-68 has several disadvantages, including unnecessary radiation exposure to operators, and difficulty to meet GMP requirements. The aim of this study was to develop an automated synthesis method for the labelling of Ga-68 UBI.

**Methods:** Ga-68 for radiolabelling was freshly eluted from an iThemba Labs Ge-68/Ga-68 generator, using fractional elution with 0.6M HCl. NOTA-UBI was provided by BL Biochem (Shanghai, China). The approach to developing an automated method was to first duplicate the manual method developed by Ebenhan et al* using the generator, eluant and consumables available at our PET Centre. Next, the manual method was adapted to suit a Scintomics protocol, e.g. adapting volumes for the synthesis unit. The radiolabelling yield and radiochemical purity were determined after each labelling experiment.

**Results** Initial manual labelling attempts indicated that the volume (± 0.55 ml) of sodium acetate required to adjust the pH of the labelling mixture was too small for use in an automated synthesis process. It was decided to use a 1.5 M HEPES buffer to adjust the eluate to an acceptable pH. Eluate volumes ranging from 1.0 to 2.0 ml were used to which 1.2 to 1.6 ml of HEPES buffer was added and the pH measured in order to find the most suitable combination of eluate volume and buffer volume to render a mixture with a pH between 3.5 and 4.0. Results indicated that 1.4 ml to 2.0 ml eluate and 1.2 ml HEPES buffer was suitable for use in the development of an automated synthesis method. Four successful automated labellings were performed using the HEPES buffer with an average decay-corrected radio-yield of 88.97% and a radiochemical purity of 99.49%.

**Conclusion:** An automated synthesis protocol using a Scintomics GRP Module has been successfully developed and tested. This protocol can be utilised for the routine synthesis of Ga-68 UBI under GMP conditions.

**Reference**

Ebenhan T.*et al.* [2014], *Nucl Med Biol* 41:390–400

## PP31 *In vitro* becteria-binding assays of radiolabeled Ampicillin loaded graphene oxide nanoflakes

### F. Yurt^1^, O. Alp Ersöz^1^, E. Harputlu^2^, K. Ocakoglu^2,3^

#### ^**1**^Ege University, Department of Nuclear Applications, Institute of Nuclear Science, Bornova, 35100, Izmir, Turkey; ^**2**^Advanced Technology, Research and Application Center, Mersin University, 33343, Mersin, Turkey; ^**3**^Department of Energy Systems Engineering, Faculty of Technology, Mersin University, 33480 Mersin, Turkey

##### **Correspondence:** F. Yurt

**Aim:** The current treatment methods such as chemotherapy have limited effects on cancer or other cells because of using conventional cytotoxic drugs. For this reason, using multifunctional nanoparticles in drug delivery systems become more important.[1,2] Some drug molecules are encapsulated nanoparticles as drug delivery systems.[3] Ampicillin is a one of effective antibiotics against Gram-positive and Gram-negative bacteria. The present study aims to label ampicillin loaded graphene oxide nanoflake (AMP-GO) with ^99m^Tc and evaluate of its *in vitro* binding to *S. aureus* and *E. coli*.

**Methods:** Graphene oxide nanoflakes were prepared according to Hummers’ method.[4] Ampicillin was loaded onto graphene oxide nanoflake prepared. AMP-GO was characterized by Fourier Transform Infrared Spectroscopy (FTIR) and Scanning Electron Microscope (SEM) techniques, and the amount of loaded ampicillin onto GO was determined by UV-Vis absorption spectroscopy. AMP and AMP-GO were labeled with ^99m^Tc using stannous chloride reducing agent. The stability of ^99m^Tc-AMP and ^99m^Tc-AMP-GO were determined in phosphate buffered saline (pH=7.2). The *in vitro* bacterial binding studies were performed by using both radiolabeled AMP and AMP-GO to *S. aureus* and *E. coli*.

**Results** Labeling efficiency of ^99m^Tc-AMP was 92.6±5.94 % while labeling efficiency of ^99m^Tc-AMP-GO was found to be 97.66±2.06%. The stability results of radiolabeled compounds in phosphate buffered saline are summarized in Fig. 1.

**Fig. 1 (abstract PP31).**
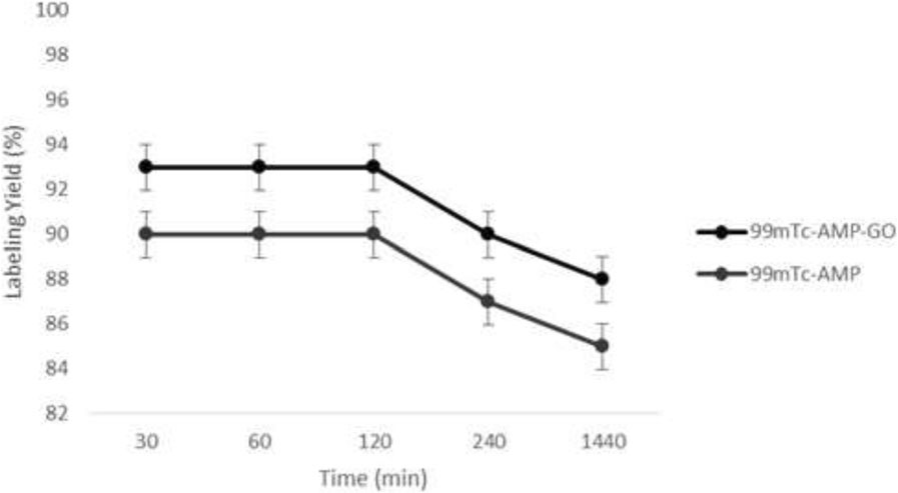
The in vitro stability of ^99m^Tc-AMP and ^99m^Tc-AMP-GO at various times after incubation in phosphate buffered saline at room temperature. Data are presented as mean ± standard deviation

In vitro binding results of ^99m^Tc-AMP/^99m^Tc- AMP-GO to S. aureus are seen in Fig. 2.

**Fig. 2 (abstract PP31).**
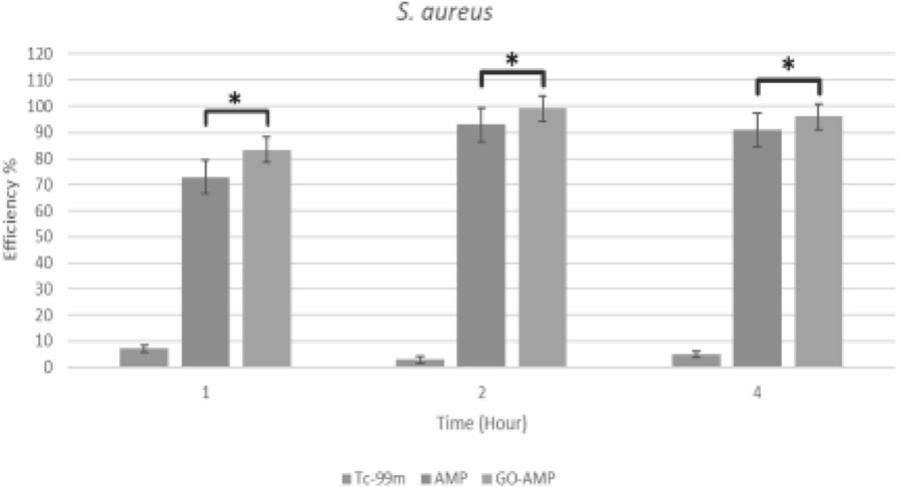
In vitro binding efficiencies of ^99m^Tc, ^99m^Tc-AMP, and ^99m^Tc-AMP-GO to S.aureus. Data are presented as mean ± standard deviation (*n* = 3) *p < 0.05

**Fig. 3 (abstract PP31).**
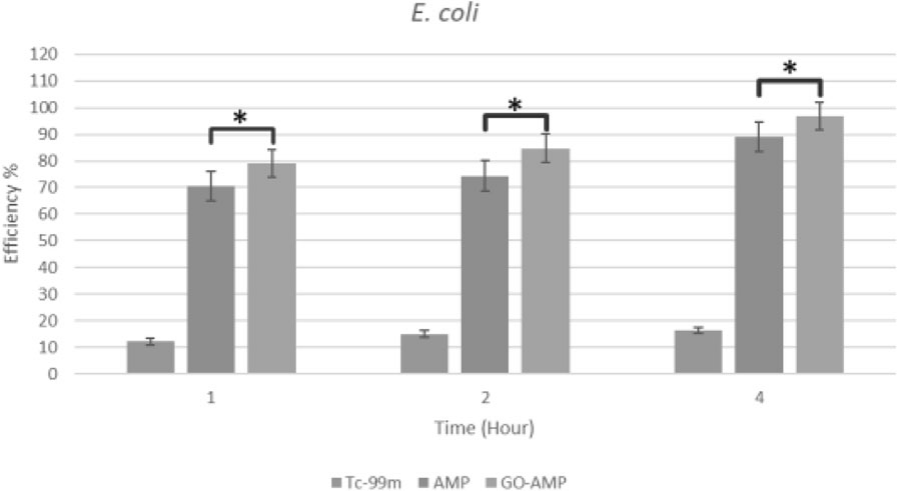
*In vitro* binding efficiencies of ^99m^Tc, ^99m^Tc-AMP, and ^99m^Tc-AMP-GO to *E. coli*. Data are presented as mean ± standard deviation (*n* = 3) * *p* < 0.05

In vitro binding results of ^99m^Tc-AMP/^99m^Tc- AMP-GO to E. Coli are seen in Fig. 3.

**Conclusion:** AMP and AMP-GO were labeled with ^99m^Tc with high yield. According to i*n vitro* binding results, the binding efficiency of^99m^Tc-AMP-GO was higher to *S. aureus* and *E. coli* than ^99m^Tc-AMP. ^99m^Tc-AMP-GO could be promising candidate as agent infection nuclear imaging. Furthermore, *in vivo* studies of ^99m^Tc-AMP-GO with infected rats are planned to be done.

**References**

1. Soppimath KS, Aminabhavi TM, Kulkarni AR, Rudzinski WE, [2001] J Control 70:1–20.

2. Slowing II JL, Vivero-Escoto CW, Wu, Lin VSY, [2008] Adv Drug Deliv Rev 60:1278–1288.

3. Hans M, Lowman A, [2002] Curr. Opin Solid State Mater Sci 6:319–327.

4. Hummers WS, Offeman Jr RE, [1958], J Am Chem Soc 80:1339.

## PP32 Room-temperature radiolabeling can be achieved by Al-^18^F chelation

### R. Pal, N. Soni, P. Tu Huynh, J. Yoo

#### Kyungpook National University, Department of Molecular Medicine, BK21 Plus KNU Biomedical Convergence Program, Daegu, Korea Republic (South)

##### **Correspondence:** R. Pal

**Aim:** ^**18**^F is the most commonly used radionuclide for PET imaging. Its half-life of approximately 110 min is suited for same-day imaging of many compounds that clear quickly from the body to allow visualization of uptake in the intended target. Recently, ^18^F-labeling method using Al−F complex in aqueous solution was devised and offered a straightforward ^18^F-labeling procedure. The radiolabeling of various chelates with Al-^18^F at high temperature (~100-110^o^C) is well documented in literature^1^. But high temperature radiolabeling is unsuitable for heat sensitive biomolecules. So facile method for radiolabeling at lower temperature is highly needed. We examine the efficiency of Al-^18^F chelation by a variety of available macrocyclic chelators at low temperature (Fig. 1).

**Methods:** ^18^F^−^ aqueous solution was first treated using a Sep-Pak cartridge equilibrated with 0.4 M KHCO_3_. The sample was then loaded, washed with water and eluted with 200-μL fractions_._ The radiolabeling was performed by adding 10 μL of the 2 mM Al^3+^ stock solution, 10 μL of the chelator stock solution, and ~500 μCi Na^18^F to a vial containing 100 μL 0.1 M ammonium acetate buffer (pH 5.5) solution and 100 μL ethanol at different temperature.

**Results:** It was found that, chelators with TACN backbones bearing two acetate pendant arms and one benzyl-NCS moiety (NODA-MP-NCS) resulted quantitative labeling in a temperature as low as 30 °C within 2 min.

**Conclusion:** These results represent an important step toward rapid and efficient radiofluorination of biomolecules at room temperature.

**Fig. 1 (abstract PP32).**
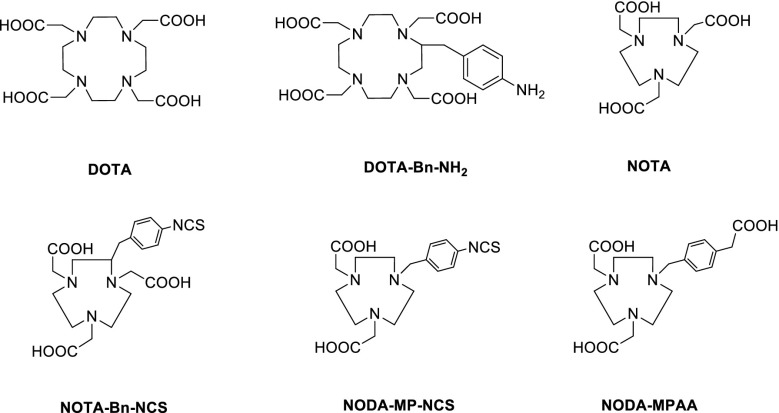
Chemical structures of the chelators used in this study

**Acknowledgements**

This work was supported by NRF (2016R1A2B4011546, 2013R1A4A1069507, 2017M2C2A1014006, 2017M2A2A6A02018506, HI17C0221) and BK21 Plus KNU Biomedical Convergence Program, Korea.

**Reference**

[1]. McBride W J, Sharkey R M, Goldenberg D M, [2013], EJNMMI Res, 3: 36.

## PP33 Pd catalyzed cross-coupling for ^11^C-PET tracer synthesis

### H. Helbert^1,2^, G. Luurtsema^2^, W. Szymanski^3^, B. L. Feringa^1^ and P.H. Elsinga^2^

#### ^1^Stratingh Institute for Chemistry, University of Groningen; ^2^Department of Nuclear Medicine and Molecular Imaging, University of Groningen, University Medical Center Groningen; ^3^Department of Radiology University of Groningen, University Medical Center Groningen

##### **Correspondence:** H. Helbert

**Aim:** A key factor for the expansion of positron emission tomography (PET) is the development of new synthetic routes for the incorporation of radionuclides. Short half-life isotopes such as carbon-11 (t_1/2_ = 20.33 min) particularly suffer from a limited number of labeling strategies. In order to expand the labeling toolbox for carbon-11, a recently discovered ultrafast cross-coupling methodology^[1]^ was applied to the labeling of several clinically interesting tracers.

**Methods:** [^11^C]MeLi was prepared via lithium-halogen exchange by trapping [^11^C]MeI in a solution of *n-*BuLi. The prepared [^11^C]MeLi was further used in a palladium catalyzed cross-coupling reaction with aryl bromides.

**Fig. 1 (abstract PP33).**
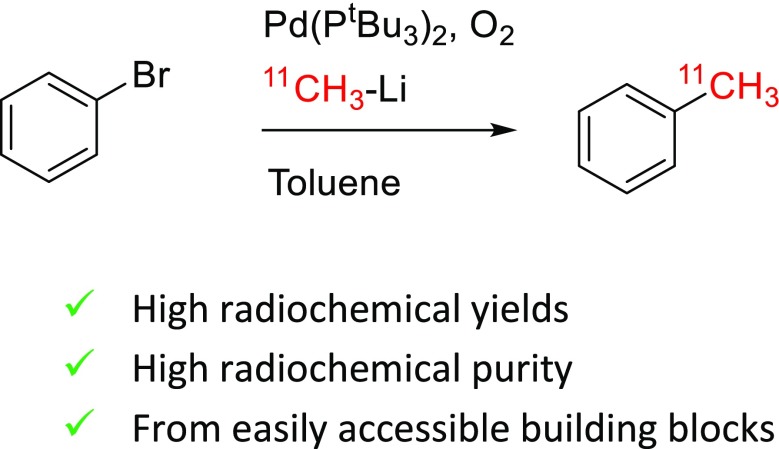
See text for description

**Results** Using this procedure, three classes of tracers with applications in amyloid plaques imaging, breast cancer imaging and VAChT imaging had been successfully labelled. Radiolabeled products were obtained in good yields with high radiochemical purity (>99%).

**Conclusion:** A new labeling methodology was developed and successfully applied to the synthesis of clinically interesting radiotracers. This procedure fulfilled essential requirements for the introduction of carbon-11, providing the target molecule in high yields and high radiochemical purity within 30 to 40 minutes from the end of bombardment (EOB).

**Reference**

1. Heijnen D, Tosi F, Vila C, Stuart M, Elsinga P, Szymanski W, Feringa B, **[2017]** *Angew. Chem. Int. Ed.* 56 (12): 3354-3359

## PP34 Effect of structural forms on the stability of linear and cyclic apoptosis-targeting peptides

### Y. Su Ha, N. Soni, J. Yoo

#### Kyungpook National University, Department of Molecular Medicine, BK21 Plus KNU Biomedical Convergence Program, Daegu, South Korea

##### **Correspondence:** Y. Su Ha

**Aim:** Apoptosis, a genetically determined process of programmed cell death, has a crucial role in various processes including normal cell turnover, tissue homeostasis, and development. Moreover, apoptosis occurs in many medical disorders and hence the development of non-invasive evaluation method is highly demanded. We have reported high potential of the radiolabeled apoptosis-targeting peptide (ApoPep-1) for apoptosis detection, however in vivo stability of radiolabeled peptide was not acceptable to monitor apoptosis for a long time [1]. In this study, we prepared cyclic ApoPep-1 peptides (Fig. 1) to compare the stability with original linear ApoPep-1. A targeting ability of cyclic ApoPep-1 peptide for apoptosis was also investigated in acute myocardial infarct model.

**Methods:** Linear and cyclic ApoPep-1 peptides were synthesized by a standard Fmoc method and an extra tyrosine residue was added for labeling with radio-iodine. The in vitro stability of the radiolabeled peptides was determined in FBS, and the in vivo stability test was performed by blood analysis. To evaluate its targeting efficacy of radiolabeled cyclic ApoPep-1, autoradiography was done in isoprenaline-induced acute myocardial infarct model.

**Results:** All linear and cyclic peptide was synthesized and radiolabeled with I-131 in good yield. The radiolabeled peptides showed significant difference in vitro stability at 24 h (linear ApoPep-1: 72%, cyclic ApoPep-1: ~95%). In vivo stability of radiolabeled linear and cyclic peptides at 1 h showed drastic difference between linear and cyclic peptides (linear ApoPep-1: 60%, cyclic ApoPep-1: 87%). Apoptosis induced in heart by myocardial infarction was clearly detected by the radiolabeled cyclic ApoPep-1.

**Fig. 1 (abstract PP34).**
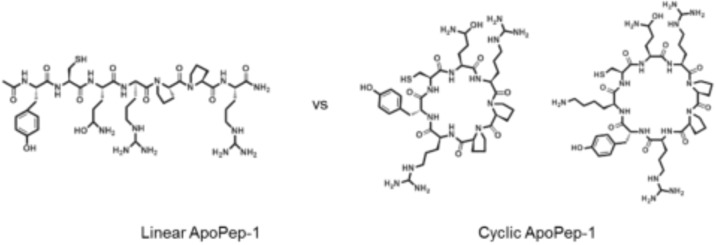
Chemical structure of linear and cyclic ApoPep-1 peptides

**Conclusion:** Effect of structural forms on the stability of linear and cyclic ApoPep-1 was demonstrated.

**Acknowledgement**

This work was supported by NRF (2016R1A2B4011546, 2013R1A4A1069507, 2017M2C2A1014006, 2017M2A2A6A02018506, 2017R1D1A1B03033974, HI17C0221, & KRF 7072016H1D3A1907667) and BK21 Plus KNU Biomedical Convergence Program, Korea.

**Reference**

1. Wonjung Kwak, et al (2015), Apoptosis, 20, 110-21.

## PP35 Synthesis and evaluation of a [^18^F]fluorinated quaternary α-amino acid-based arginase inhibitor

### G. S. Clemente^1^, I. F. Antunes^1^, S. Kurhade^2^, A. Dömling^2^, P. H. Elsinga^1^

#### ^1^Department of Nuclear Medicine and Molecular Imaging, University Medical Center Groningen, University of Groningen, Groningen, The Netherlands; ^2^Department of Drug Design, University of Groningen, Groningen, The Netherlands

##### **Correspondence:** G. S. Clemente

**Aim:** Arginase catalyzes the hydrolysis of arginine to ornithine and urea, being the final enzyme of the urea cycle, which is a ubiquitous pathway to excrete toxic ammonia from organisms. Lately, it also has emerged as a key regulator of nitric oxide by competing with NO synthase for the same substrate. Therefore, arginase overexpression has been associated with a series of pathogenic processes that can go from cardiovascular, immune-mediated or inflammatory conditions to tumor cell metabolism.^(1)^ Several research groups have been involved in the development of small-molecule arginase inhibitors reaching to data holding great promise. However, only recently the drug development industry is attaining clinical trials.^(2)^ Since the association to PET labeling techniques has great relevance not only for the evaluation and characterization of some of this molecules, but also to increase the library of radiotracers available, our goal with this work was to synthesize and evaluate the [^18^F]fluorinated equivalent of a late-generation arginase inhibitor.^(3)^

**Methods:** An arylboronic acid pinacol ester-derived precursor was synthesized in view of a Cu-mediated nucleophilic [^18^F]fluorination. Radiochemical yield for the conversion of the intermediate species was assessed by TLC-SG and/or radioHPLC. Deprotection of the amino acid moiety was achieved by hydrolysis and the final radiotracer was purified by semi-preparative HPLC and obtained, after reformulation, as a physiological and injectable solution. Further stability and distribution-coefficient studies were performed.

**Fig. 1 (abstract PP35).**
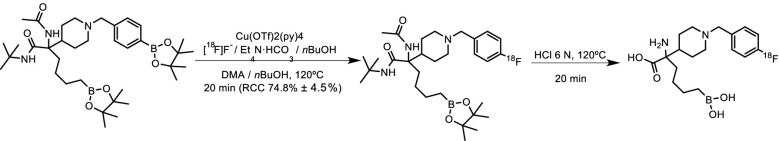
See text for description

**Results:** In summary, radiochemical yield of the conversion of the arylboronic ester-derived precursor reached ~80% when using 60 to 15 μmol (lower amounts brought significant losses in yield). Final [^18^F]fluorinated compound was obtained with radiochemical purity ≥95% in an overall yield of 11% (d.c., non-automated synthesis). The radiotracer showed stability in solution up to 4 h and an experimental *log* D of -0.67±0.05.

**Conclusion:** The [^18^F]fluorinated arginase inhibitor was efficiently labeled with fluorine-18 in good yield. Preliminary in vitro studies using R22v1 cell lines, which express Arg2, revealed a cellular uptake of the radiotracer susceptible of being blocked after treatment with arginase inhibitors. Further studies are currently being performed in different cell lines which either overexpress Arg1 or Arg2 to evaluate the potential of the developed radiotracer towards arginase mapping.

**References**

1. Caldwell RB, Toque HA, Narayanan SP, Caldwell RW, [2015], Trends Pharmacol Sci, 36(6):395-405

2. Pudlo M, Demougeot C, Girard-Thernier C, [2017], Med Res Rev, 37(3):475-513; (3) Van Zandt M, Golebiowski A, Ji MK, Whitehouse D, Ryder T, Beckett P, [2011], WO Patent 2011/133653A1.

## PP36 Facile synthesis of 6-L-[^18^F]fluoro-*m*-tyrosine via alcohol-enhanced Cu-mediated radiofluorination of Bpin-substituted chiral Ni-BPB-AA complex

### ^1^N.P. Bechtereva Institute of Human Brain, Russian Academy of Science, St-Petersburg, Russian Federation; ^2^Institute of Neuroscience and Medicine, INM-5: Nuclear Chemistry, Forschungszentrum Jülich, Germany; ^3^Institute of Radiochemistry and Experimental Molecular Imaging University Clinic Cologne; ^4^Max Planck Institute for Metabolism Research, Cologne, Germany

**Aim:** The improved signal to noise ratio and simplified kinetic modeling^1^ has presented 6-L-[^18^F]fluoro-*m*-tyrosine (6-[^18^F]FMT) as a valuable alternative to 6-L-[^18^F]FDOPA, a well-known PET tracer for the visualization of dopaminergic neurons and neuroendocrine tumors. However, clinical applications of 6-[^18^F]FMT have been limited by the paucity of practical production methods. Cu-mediated radiofluorination of pinacol arylboronates (aryl-Bpin)^2^ enables the preparation of radiolabeled aromatics regardless of their electronic properties using nucleophilic [^18^F]fluoride. We have applied our procedure for alcohol-enhanced Cu-mediated ^18^F-fluorination^3,4^ to prepare 6-[^18^F]FMT using an easily accessible Bpin-substituted Ni-BPB-AA complex as a dual-protecting framework.

**Methods:** Radiolabeling precursor was synthesized by alkylation of (*S*)-Ni-BPB-Gly with 6-Bpin-3-MOMO-benzyl bromide prepared via radical bromination of the corresponding toluene. Aqueous [^18^F]fluoride was loaded onto a QMA vac 1cc cartidge from the male side, the cartridge was rinsed by 1.5 mL of *i*-PrOH and dried with helium. ^18^F was eluted in the opposite direction using a solution of 12.5 μmol of TBAOTf in 0.6 mL *i*-PrOH directly to a solution of 7.5 μmol of Cu(OTf)_2_Py_4_, 15 μmol of **I** in 0.3 mL DMA. The mixture was heated in a sealed vial at 110°C for 20 min under air. 1.0 mL of 12 M HCl was added and the reaction mixture was heated (100^o^C, 10 min) furnishing the crude 6-[^18^F]FMT which was purified by HPLC using 2% EtOH in 0.1% AcOH as eluent. The product fraction was additionally purified using a SepPak Alumina N light cartridge.

**Results:** Precursor for radiolabeling was prepared in 45% yield over two steps. The developed radiolabeling protocol provides a high ^18^F-elution efficiency of 86-94% and ^18^F-incorporation rate of > 90% (determined by TLC). Deprotection afforded the desired tracer in a RCC of 60%. 6-[^18^F]FMT was produced in a RCY of 32%, RCP of 97% and enantiomeric purity > 99% within 70 min (EOB).

**Conclusion:** Boronated Ni-BPB-AA complexes are valuable substrates for the preparation of labeled aromatic amino acids using alcohol-enhanced Cu-mediated radiofluorination. The novel protocol enables the simple and efficient production of 6-[^18^F]FMT without time consuming solvent evaporation and intermediate SPE purification steps.

Research support: RFBR grant № 16-54-12062\16 and DFG grant ZL 65/1-1.

**References**

1. C. Li et al., *Synapse*, **2014**, 68(8), 325-331.

2. M. Tredwell et al., *Angew. Chem. Int. Ed.*, **2014**, 53(30), 7751-7755.

3. J. Zischler et al., *Chem. Eur. J.,* **2017**, 23, 3251-3256.

4. B. Zlatopolskiy et al., *J. Med. Chem.,* **2017**, doi: 10.1021/acs.jmedchem.7b01245e.

## PP37 Physico-chemical assessment of labeled freeze dried kits of trastuzumab-immunoconjugates significant for breast cancer therapy

### M. Sterjova^1,2^, P. Džodić^2^, P. Makreski^3^, T. Ruskovska^1^, E. Janevik-Ivanovska^1^

#### ^1^University ‘Goce Delčev’, Faculty of Medical Sciences, str. “Krste Misirkov” No. 10-А, 2000 Štip, Republic of Macedonia; ^2^University of Niš, Faculty of Medicine, Department of Pharmacy, str. dr Zoran Đindić 81, 18000 Niš, Serbia; ^3^Institute of Chemistry, Faculty of Natural Sciences and Mathematics, Ss. Cyril and Methodius University, 1000 Skopje, Republic of Macedonia

##### **Correspondence:** M. Sterjova

**Aim:** Monoclonal antibodies appears as an important therapeutic agents for cancer treatment and have shown high complexity in the manner of action and their biological properties. Due to the significant potency in various malignancies and easy detection of radioactivity with outside scintigraphy, radioimmunoconjugates have become a part of many clinical trials. The antibody manipulation and exposure to stress conditions during the processes of conjugation, lyophilization and labeling, can cause disruption of the native structure of the protein. The most appropriate technique for protein integrity and purity examinations is reducing sodium dodecyl sulfate polyacrylamide gel electrophoresis (SDS-PAGE). Attenuated total reflectance-infrared (ATR-IR) and Raman spectroscopy as powerful, non-destructive and easy-to-use techniques provide valuable molecular structure information and are convenient for verification of changes in the secondary structure. The successful experience in formulation of ready-to-use kits of rituximab-immunoconjugates, inspire us to use another attractive monoclonal antibody for therapy of HER2 positive breast cancer. The same protocols, with minor adjustments, were implemented for structural characterization of labeled and non-labeled lyophilized trastuzumab-immunoconjugates formulations.

**Methods:** The purified trastuzumab from Herceptin^®^ was conjugated with bifunctional chelators (BFCAs), p-SCN-Bn-DTPA, p-SCN-Bn-DOTA, *p*-SCN-Bn-1B4M-DTPA in ratio of 1:20 and lyophilized to solid state. The freeze dried conjugates and cold labeled samples with LuCl_3_ and YCl_3_ were used for further examinations and physico-chemical characterization by applying of SDS-PAGE, ATR-IR and Raman spectroscopy.

**Results** Under reducing conditions migration of the trastuzumab provided separation of two bands of fragments with molecular weight of 25 kDa for light chain and 50 kDa for heavy chain, proven with the Wide Range, Molecular Weight marker. The same intensity of the fragments of lyophilized and labeled conjugates with the fragments of pure trastuzumab was indicated that there is no degradation of the antibody. ATR-IR and Raman spectra also have indicated that all samples have retained native structure expressed in terms of assignment of the amide bands (amide I, II and III bands), characteristic for IgG1 structures principally composed of β-sheets. Characteristic amide I band at ~1670 cm^-1^ and amide III band (1230-1300 cm^-1^) were detected in Raman spectra. IR spectra also contain the amide I (1700-1600 cm^-1^), amide II (1480-1575 cm^-1^) and amide III bands (1255-1244 cm^-1^) specific for secondary structure of the proteins.

**Conclusion:** The promising results from electrophoresis and vibrational spectroscopy are good basis for further radiolabeling of immunoconjugates with ^177^Lu and ^90^Y for treatment and imaging of HER2 positive lesions.

**References**

1. Fabian H, Mantele W, [2002] BA. 5: 1-27.

2. Gjorgieva Ackova D, Smilkov K, Janevik-Ivanovska E, [2014], WJMS 11: 535-540.

3. Gjorgieva Ackova D, Smilkov K, Janevik-Ivanovska E, [2016], IJPR 3: 295-302.

4. Gjorgieva Ackova D, Smilkov K, Janevik-Ivanovska E, Stafilov T, Arsova-Sarafinovska Z, Makreski P, [2015], MJCCE 34: 351-362.

5. IAEA Coordinated Research Project (CRP): Therapeutic radiopharmaceuticals based on 177Lu and 90Y labeled monoclonal antibodies and peptides: Development and preclinical evaluations.

6. Kengne-Momo RP, Daniel P, Lagarde F, Jeyachandran JL, Pilard JF, Durand-Thouand MJ, Thouand G, [2012] IJS 2012: 1-7.

7. Nebija D, Noe CR, Urban E, Lachmann B, [2014] IJM 15: 6399-6411.

## PP38 Development of activity-based PET probes for selective detection of active caspase-3

### F. Elvas^1,2,3^, A. Solaina^4^, P. Van der Veken^2^, K. Augustyns^2^, S. Stroobants^1,3^, D.W. Wolan^4^, L. Wyffels^1,3^

#### ^1^Molecular Imaging Center Antwerp, University of Antwerp, Antwerp, Belgium; ^2^Laboratory of Medicinal Chemistry, University of Antwerp, Antwerp, Belgium; ^3^Department of Nuclear Medicine, Antwerp University Hospital, Antwerp, Belgium; ^4^Departments of Molecular and Experimental Medicine and Chemical Physiology, The Scripps Research Institute, La Jolla, California, United States

##### **Correspondence:** F. Elvas

**Aim:** Apoptosis represents the most abundant type of regulated cell death, and plays a key role in disease treatment. Many therapies targeted at promoting tumor apoptosis are currently in use for the treatment of cancer patients. Therefore, assessment of response to anticancer therapies can be achieved by readout of apoptosis in tumors. Non-invasive molecular imaging of therapy-induced apoptosis can be achieved by using positron emission tomography (PET) radiotracers that specifically target hallmarks of the apoptosis process such as the activation of caspase-3. We therefore aimed to develop ^18^F-labeled activity-based probes (ABPs) to selectively monitor caspase-3 activity by PET imaging.

**Methods:** Our concept is based on turning selective and potent peptide-based inhibitors into ABPs. For this Hyx-(Ahx)_2_-DW3-KE was developed bearing an optimized peptide sequence (DW3) for the selective detection and inhibition of caspase-3 in cells, and an electrophilic center (warhead, KE) for covalent binding to the enzyme active site. Radiolabeling of Hyx-Ahx_2_-DW3-KE with ^18^F was performed using the copper-catalyzed azide-alkyne cycloaddition reaction. The binding efficiency of the ^18^F-labeled ABP, [^18^F]MICA-304, to human caspases will be evaluated using SDS-PAGE analysis. Radiotracer metabolic stability will be evaluated *in vitro*. The *in vitro* affinity and selectivity of the non-radioactive ABP will be profiled against a panel of human caspases.

**Results** “Click radiolabeling” was used to obtain [^18^F]MICA-304 from [^18^F]fluoroethylazide in a radiochemical yield ranging from 55 to 81% (based on HPLC analysis of the crude product, Table 1).

**Table 1 (abstract PP38).** Radiochemical yield of [^18^F]MICA-304

	Reaction time		
Amount of precursor	10 min	20 min	
4 mg	81%	55%*	76%
8 mg		78%	

Click reaction mixture: 2.2 eq. CuSO_4_; 8.2 eq. sodium ascorbate; 1.1 eq. bathophenanthroline disulfonate. *Using half of the amount of the click reaction mixture

**Conclusion:** Optimal radiochemical reaction conditions were defined to be 10 minutes at room temperature. *In vitro* and preliminary *in vivo* data will be reported. [^18^F]MICA-304 is a candidate ABP for selective detection of caspase-3 activity, and will be modified for *in vivo* PET imaging in mouse models of apoptosis.

## PP39 Chemical analysis of cyclotron-based [^68^Ga]GaCl_3_ by ICP-MS

### J. Frigell, K. Gagnon

#### GE Healthcare, Uppsala, Sweden

##### **Correspondence:** K. Gagnon

**Aim:** P Current limitations with existing ^68^Ge/^68^Ga generators have led to a recent interest in the direct cyclotron production of ^68^Ga by the ^68^Zn(p,n)^68^Ga reaction. To this end, we recently presented a new automated two-column scheme to isolate [^68^Ga]GaCl_3_ following proton irradiation of enriched ^68^Zn (Nair M. et al [2017], Eur J Nucl Med Mol Imaging 44: S119–S956). Optimization of this process was focused not only on maximizing ^68^Ga recovery, but also on meeting specifications (e.g. <10 μg/GBq iron and zinc) of the European Pharmacopoeia (EUP) generator-based [^68^Ga]GaCl_3_ monograph. A detailed assessment of other chemical impurities was, however, considered warranted and forms the basis for this study.

**Methods:** A selection of sample retains (Zn Lot A: Feb-May, 2017; Zn Lot B: Aug-Oct, 2017) from experiments performed on a GE PETtrace ^68^Ga liquid target/FASTlab system were submitted for ICP-MS analysis (ALS Scandinavia). Samples were either irradiated (but not chemically processed), or, samples were chemically isolated as ~2.8 mL [^68^Ga]GaCl_3_. For the analysis of Zn, it is critical to note that the m/z = 68 channel was used, along with appropriate scaling to account for the non-natural abundance of ^68^Zn. Natural isotopic composition was assumed for the analysis of ~70 additional elements. It is also imperative to note that samples were taken during the development phase, and may include sub-optimal chemistry processing and in some cases, ACS grade reagents. This wide range of samples was nevertheless included to provide insight to the process variability and robustness.

**Results:** Data is tabulated below for both Zn and Fe (in μg/mL), as well as 8 other impurities (in ng/mL). These additional impurities were identified to be of interest due to known target contact materials, noted chemical variation between ^68^Zn lots, potential concern for DOTA chelation competition with Ga, etc. Data is reported as the average and standard deviation for the noted number of samples. It should also be noted that current levels of ^68^Zn are routinely below 1 μg/mL, with further reduction demonstrated with additional column rinsing.

**Table 1 (abstract PP39).** See text for description

		*Zn Lot A*		*Zn Lot B*	
		Irradiation only (*n* = 3)	Isolated [^68^Ga]GaCl_3_ (*n* = 9)	Irradiation only (*n* = 5)	Isolated [^68^Ga]GaCl_3_ (*n* = 6)
*μg/mL*	Zn	48583 ± 365	2.8 ± 3.4	55183 ± 1066	0.73 ± 0.48
	Fe	1.13 ± 0.12	0.24 ± 0.12	0.98 ± 0.13	0.18 ± 0.12
*ng/mL*	Ni	1318 ± 118	18 ± 11	214 ± 35	19 ± 13
	Cu	1615 ± 121	8.8 ± 6.1	1147 ± 152	4.7 ± 1.2
	Co	85 ± 74	0.28 ± 0.19	3.8 ± 1.1	0.150 ± 0.055
	Cr	37 ± 20	22 ± 24	46.0 ± 8.7	10.2 ± 5.2
	Y	0.60 ± 0.10	0.38 ± 0.14	0.380 ± 0.045	0.283 ± 0.075
	Nb	21 ± 35	0.046 ± 0.014	18 ± 35	0.14 ± 0.11
	Sn	9 ± 12	1.19 ± 0.61	335 ± 192	21 ± 29
	Cd	4.6 ± 3.5	0.19 ± 0.15	1940 ± 87	0.22 ± 0.12

**Conclusion:** While the levels of Zn and Fe have been optimized to meet EUP specifications, a detailed assessment of additional chemical impurities has revealed that the starting contribution (i.e. post irradiation, but pre-chemical processing) for impurities of potential concern is generally quite low – e.g. at most ~1-2 μg/mL, with post-purification reducing such impurities to the single-to-low-tens of ng/mL range. This analysis supports the current applicability of cyclotron-based production of ^68^Ga and anticipated robustness as novel ligands and chelators are developed and introduced.

## PP40 Automation of Click Chemistry for the synthesis of ^18^F-labelled PSMA-tracers using the FlowSafe

### V. I. Böhmer^1^, D. van der Born^4^, W. Szymanski^2,3^, M. Klopstra^5^, T. J. Visser^5^, B.L. Feringa^3^, P.H. Elsinga^1^

#### ^1^Department of Nuclear Medicine and Molecular Imaging, University of Groningen, University Medical Center Groningen, Hanzeplein 1, 9713 GZ Groningen, The Netherlands; ^2^Department of Radiology, University of Groningen, University Medical Center Groningen, Hanzeplein 1, 9713 GZ Groningen, The Netherlands; ^3^University of Groningen, Stratingh Institute for Chemistry, Nijenborgh 4, 9747 AF Groningen, The Netherlands; ^4^FutureChemistry, Toernooiveld 100, 6525 EC, Nijmegen, The Netherlands; ^5^Syncom, Kadijk 3, 9747 AT Groningen, The Netherlands

##### **Correspondence:** V. I. Böhmer

**Aim:** Automated PET-tracer syntheses are essential to enable productions in a quick and robust way for (pre)clinical studies [1]. In this study, azide-alkyne Huisgens cycloadditions of ^18^F-labelled azide building blocks (azide-BB) with acetylene-ligands are used for the development of ^18^F-labelled PET-tracers for diagnosis of prostate cancer by targeting prostate specific membrane antigen (PSMA). Due to purification issues of the azide-BB’s, the radiochemical yields (RCY) of these click reactions (CR) were low. Automated microfluidic systems, such as the FlowSafe, could improve these syntheses. The advantages of ^18^F-labelling in flow-mode comprises the more dynamic mixing and heating of the azide-BB precursor and [^18^F]fluoride, leading to higher yields and less side-products due to shorter reaction times and higher surface-to-volume ratios [2]. Therefore, we elaborated an automated synthesis route for ^18^F-labelled PSMA-tracers (^18^F-PSMA-tracer) in the FlowSafe, using a 2-steps procedure in which an azide-BB is ^18^F-fluorinated in a microreactor, purified by Solid Phase Extraction (SPE) and subsequently underwent an in-batch CR.

**Methods:** Azeotropically dried [18F]fluoride and the precursor for azide-BB are redissolved in acetonitrile, transferred through a 100μL glass microreactor with a flowrate of 80μL/min at 110oC, giving an effective reaction time of 75s. A back-pressure regulator adjusted the pressure inside the microreactor to 5.0bar and increased the boiling point of acetonitrile. The intermediate is dissolved in an excess of water and transferred to an Oasis HLB Plus-cartridge. It is eluted with ethanol and collected in a vial containing click reagents and PSMA-targeting acetylene-ligand. CR is performed in a batch-reactor in at least 50% water, heated to 80oC for 20min and subsequently purified by HPLC.

**Results:** Manually 18F-fluorination of the azide-BB gave a RCY of 21%. Performing the 18F-fluorination of the azide-BB in the FlowSafe reduced radiochemical impurities from 2.7% “by hand” to 0.3%. With the purer intermediate, the SPE purification-method was improved, providing the azide-BB in >99% radiochemical purity and a RCY of 39%. With this purified azide-BB, a CR was performed yielding a 18F-PSMA-tracer with an isolated RCY of 5%.

**Conclusion:** Automation of ^18^F- PSMA-tracer synthesis was successful. Less side-products were formed during the ^18^F-fluorination of the azide-BB in the microreactor, which simplified the purification and led to an improved RCY. With the purified ^18^F-labelled azide-BB, a novel ^18^F-PSMA-tracer was successfully coupled in-batch by CR in the FlowSafe.

**References**

1. Pascali G. *et al*, **2013**, *J Nuc Med Bio*, 40, 776 – 787

2. Rensch C. *et al*. **2013,** *Molecules*, 18, 7930-7956

## PP41 [^18^F]-FEPPA, a 2^nd^ generation of TSPO radioligand: optimized radiosynthesis and quality control

### N. Vignal^1,2^, C. San^1^, F. Hontonnou^1,3^, T. Gelé^1^, A. Cossé^1^, X. Declèves^4^, L. Sarda-Mantel^1,5,6^, S. Cisternino^7^, N. Rizzo-Padoin^1,2^, B. Hosten^1,2^

#### ^1^Assistance Publique – Hôpitaux de Paris, Hôpital Saint-Louis, Unité Claude Kellershohn, Paris, France; ^2^Inserm UMR-S 1144, Faculté de Pharmacie de Paris, Université Paris Descartes, Paris, France; ^3^Université Paris Diderot, Institut Universitaire d’Hématologie, Paris, France; ^4^Assistance Publique – Hôpitaux de Paris, Hôpital Cochin, Paris, France; ^5^Assistance Publique – Hôpitaux de Paris, Hôpital Lariboisière, Paris, France; ^6^Inserm UMR-S 942, Université Paris Diderot, Paris, France; ^7^Assistance Publique – Hôpitaux de Paris, Hôpital Necker – Enfants Malades, Paris, France

##### **Correspondence:** N. Vignal

**Aim:** [^18^F]-FEPPA, a radiotracer specific for TSPO (18 kDa translocator protein), is used in PET imaging (Positron Emission Tomography) as a biomarker of cerebral inflammation. Literature [1] describes the use of methanol and formic acid in the mobile phase during semi-preparative HPLC purification. Aims of this work were to implement a radiosynthesis of [^18^F]-FEPPA using HPLC purification more suitable for *in vivo* use and to optimize the analytical HPLC control method.

**Methods:** [^18^F]-FEPPA is synthesized by nucleophilic substitution (90°C, 10 min) from a tosylateprecursor (N-(2-((N-(4-phenoxypyridin-3-yl)acetamido)methyl)phenoxy)ethyl-4-methybenzene sulfonate) followed by semi-preparative HPLC purification (mobile phase: ethanol/water for injection 70/30 v/v + 0.1% phosphoric acid, FEPPA retention time = 13 min) on an AllInOne^®^ radiosynthesis automate. Chemical, radiochemical purity (RCP) and specific activity (SA) are determined by analytical HPLC (Kinetex^®^ C18 column (50 × 2.1 mm, 2.6 μm), injection volume: 5 μL, mobile phase: H_2_O/CH_3_CN 75/25 v/v at 0.6 mL/min).

**Results** [^18^F]-FEPPA radiosynthesis were carried out in 49 min with a non-decay corrected yield enhanced from 19 to 29%. The RCP obtained was greater than 99%. The mean SA was 198 GBq/μmol. The new analytical HPLC method allowed a quality control in 7 min. Solvent consumption and injection volume have been reduced by a factor 12 and 4 respectively. This new control method induced a 3 fold decrease of the received dose by the operator in terms of ionizing radiation.

**Conclusion:** [^18^F]-FEPPA radiosynthesis was implemented with high yields. The final product was obtained in a new formulation more suitable for *in vivo* studies. Operator exposure was improved with the new faster control method.

**References**

[1]. Vasdev N, Green DE, Vines DC, McLarty K, McCormick PN, Moran MD, Houle S

[2]. Wilson AA, Reilly RM, [2013], Cancer Biother Radiopharm 28: 254-259

## PP42 Bioorthogonal chemistry applied to bispecific antibody manufacturing

### L. Navarro^1,*^, P. Remaud-Le Saëc^1,*^, AS. Navarro^1^, M. Mougin-Degraef^1^, F. Guérard^1^, JF. Gestin^1^, M. Chérel^1,2,3^ and A. Faivre-Chauvet^1^

#### ^1^CRCINA, INSERM, CNRS, University of Angers, University of Nantes, Nantes, France; ^2^Nuclear Medicine Department, ICO-René Gauducheau Cancer Center, Saint Herblain, France; ^3^GIP ARRONAX, Saint Herblain, France

##### **Correspondence:** L. Navarro

^*^Contributed equally to this work

**Aim:** Bispecific antibody (BsAb) can be obtained both chemically and bio-engineered for imaging and therapeutic applications in nuclear medicine^(1-2)^. If all the described tools are satisfactory for imaging, the *in vivo* instability of engineered BsAb or the poor yield of BsAb obtained by chemically limit their use in radioimmunotherapy (RIT). We propose in this study a new way to generate BsAb using click chemistry tools.

**Methods:** The 9E7.4 IgG_2a_ (anti-CD138) antibody was chosen for proof of concept. 9E7.4 was first modified with 100-500 equivalents of *N*-ethylmaleimide (NEM) to saturate the accessible thiol groups on the antibody surface. The modified antibody was then purified by gel filtration chromatography. The modified (9E7.4-NEM) and unmodified antibodies (9E7.4) were cleaved with optimized pepsin quantities under acidic conditions to give (Fab’)_2_ fragments. The (Fab’)_2_ obtained were then reduced with 2-mercaptoethylamine to generate the Fab’ fragments. These fragments were separated into two fractions and functionalized with dibenzocyclooctyne (DBCO) for the first fraction and with azide (N_3_) for the second one. Both functionalized Fab’ were then mixed 1 hour or1 month after production to obtain BsAb in order to validate their stability and their reactivity over time. 9E7.4 IgG, 9E7.4-NEM IgG, 9E7.4 (Fab’)_2_, 9E7.4-NEM (Fab’_2_) and BsAb were monitored by UPLC and electrophoresis for chemical purity, and after ^125^I radiolabeling for antigen recognition.

**Results** Immunoreactivity was verified after each step described above. For BsAb, the antibody tested had the best final yield (20%) and the lowest aggregates percentage correscponding to a pre-saturation with 100 eq NEM and addition of 20 eq of DBCO and 20 eq of N3 for bioorthogonal chemistry.

The results are summarized in the following Table 1.

**Table 1 (abstract PP42).** See text for description

Antibody (9E7.4)	IgG	IgG-NEM	IgG-NEM	IgG-NEM	IgG-NEM	(Fab’)_2_	(Fab’)_2_-NEM	BsAb (Fab’)_2_-NEM
		100 eq NEM/2h	100 eq NEM/12h	500 eq NEM/2h	500 eq NEM/12h		100 eq NEM/2h	100 eq NEM/2h
Immunoreactivity (%)	73	70	71	52	45	74	73	45

**Conclusion:** We demonstrated that click chemistry can be used to generate BsAb and that cysteine pre-saturation by NEM limits the formation of aggregates during the antibody functionalization. The yields obtained are comparable to those described in the literature with phenylenebismaleimide^(3)^. The immunoreactivity decrease is probably due to the large spacing between the Fab’ fragments provided by the PEG chains. This problem can be solved by the use of new click modifiers presenting a minimal distance between the fragment and the click function.

**References**

1. Scheer JM, Vandlen R, [2012], Plose one, 7, 12: 51817

2. Luo H, Cai W, [2015], PNAS, 112: 12806-12811

3. Glennie MJ, McBride HM, [1987], J Immunol, 139 :2367-2375

## PP43 A fast and robust quantification of residual solvents in radiopharmaceuticals by UHPLC

### J. Fouque, S. Huguet, E. Da Costa Branquinho, T. Rusu, S. Blondeel, K. Rezaï and O. Madar

#### Department of Radiopharmacology, Institut Curie, Saint Cloud, France

##### **Correspondence:** E. Da Costa Branquinho

**Aim:** Radiopharmaceutical preparations for human purpose may contain residual solvents. Headspace Gas Chromatography (HS-GC) remains the standard method for the analysis of volatile solvents, according to the European Pharmacopeia. Dimethylsulfoxide (DMSO), dimethylformamide (DMF) and dimethylacetamide (DMA) are less volatile solvents that are widely used in carbon-11 and fluorine-18 labeling. However, in that case, HS-GC quantification takes too much time for the release of short half-lives radiopharmaceuticals. Therefore, we developed a fast and robust Ultra High Pressure Liquid Chromatography (UHPLC) method for the simultaneous quantification of those 3 solvents.

**Methods:** Analyses were carried out on an UHPLC (Waters) using a BEH C18 column (Waters). Validation was performed within the requirements of ICH validation guidelines (Q2A, Q2B and Q3C) and based on Good Laboratory Practices (GLP). It consisted in the evaluation of linearity, limit of quantification (LOQ), limit of detection (LOD), selectivity, specificity, carry-over, accuracy, precision and matrix effect.

**Results** The raw data were collected on six different days with a minimum of five days of exploitable data for each solvent. Separation of the three solvents can be achieved within 4 minutes with high resolutions. Solvents in samples can be quantified with good inter or intraday accuracy and precision. LOQ is far below the permitted daily exposure of each solvent. Selectivity and specifity were satisfactory. No matrix effect was observed.

**Conclusion:** This fast UHPLC method is routinely used for the quality control of radiopharmaceuticals produced in our facilities. Moreover, stability of stock solutions and frozen quality controls is achieved for at least 10 months. The use of a 98% aqueous mobile phase reduces the risk of coelution of radiolabeled compounds which makes this method suitable with most of them.

## PP44 Comparison between two octreotide derivatives for somatostatin receptor scintigraphy

### A. Toumi^1,2^, A. Arab^1,2^, N. Ayachi^1,2^, M. Guezguez^2^

#### ^1^Radiopharmacy Unit, Sahloul Hospital, Sousse, Tunisia; ^2^Nuclear Medecine department, Sahloul Hospital, Sousse, Tunisia

##### **Correspondence:** A. Toumi

**Aim:** Somatostatin receptor scintigraphy reveals various types of neuroendocrine tumors. The radiotracer historically used is Indium-111 pentetreotide (^111^In-DTPA-Phe-Octreotide) = Octreoscan®. It is an analog of somatostatin’s specific membrane receptors labeled with Indium111. A new derivative of octreotide coupled to Technetium 99m (^99m^Tc-HYNIC-[D-Phe,Tyr-octreotide) = Tektrotyd® is marketed. In this study, we propose to compare between these two products according to our experience.

**Methods:** This comparative study focused on several criteria: method of preparation and administration of the product, dosimetry, patient exam preparation, image acquisition and quality. For this, we relied on the summary of products characteristics and on the examinations made at the department.

**Results** Both products require extemporaneous labeling; Tektrotyd® nevertheless has some preparation steps in addition to Octreoscan®. The preparations quality control is carried out for both of them by the same method (Thin Layer Chromatography) which is convenient for the hospital routine. The dosage varies from one product to another. Therefore, for an adult, the effective dose received for the maximum dose of 220 MBq of ^111^In-pentreotide is 12 mSv while 740 MBq of ^99m^Tc-Octreotide gives an effective dose of 3.7mSv. Image acquisition is done the day of the injection for Tektrotyd® (after 4 hours) unlike Octreoscan®, where the acquisition is done after 24 hours. Patient exam preparation is done the same way for both products. Our physicians noticed that scintigraphy performed with Tektrotyd® showed a better image quality making it easier to interpret.

**Conclusion:** Technetium 99m is a gamma emitter (141Kev) with short half-life (6 hours), while Indium 111 is a 94% gamma emitter (247Kev) with a longer half-life (2.8 days). From a dosimetric point of view, Indium 111 is much more irradiant. Thus Tektrotyd® is more convenient to use either for patients or for the staff. Some studies demonstrated that both products are equally effective despite all these differences. In our department, doctors reported a better image quality with Tektrotyd®. The fact that Tektrotyd® is presented in the form of cold kit with Technetium 99m labeling, with less radiations and better images’ quality than Indium 111, makes us chose it for scintigraphy of neuroendocrine tumors in our department.

## PP45 Comparison of automated and manual labeling methods for somatostatin analogues with the example of DOTANOC and SomaKit TOC®

### M. Frindel^1^ and A. Rauscher^1^, P. Baumgartner^1^, A. Faivre-Chauvet^2,3^, N. Varmenot^1,3^, F. Kraeber-Bodéré^1,2,3^, C. Ansquer^2^

#### ^1^Institut de Cancérologie de l’Ouest – René Gauducheau, Boulevard Jacques Monod 44805 SAINT-HERBLAIN (NANTES), France; ^2^Centre Hospitalo-Universitaire de Nantes, 1 place Alexis Ricordeau 44000 NANTES, France; ^3^Nantes-Angers Cancer Research Center, INSERM U892, CNRS UMR 6299, University of Nantes, Nantes, France

##### **Correspondence:** M. Frindel; A. Rauscher

**Aim:** ^68^Ga-edotreotide, PET-agent for somatostatin receptor imaging in GEP-NET, has recently obtained a marketing authorization as a radiopharmaceutical kit (SomaKit TOC®). We wanted to compare the performance, ease of realization, radiation protection parameters of automated labeling of DOTANOC as compared to the labeling of DOTATOC (using SomaKit TOC) with Ga-68.

**Methods:** Automated radiolabeling of DOTANOC was performed on the ModularLab PharmTracer® module (Eckert & Ziegler) with C4-Ga68-PP cassette. Process is fully automatized and includes generator elution, cation exchange post-processing, heating, purification on C18 cartridge and dilution with sterilizing filtration. Manual preparation of SomaKit was performed according recommendations and with equipment supplied by AAA. The generator is eluted with a syringe pump directly into the SomaKit vial. Buffer is then added by hand before transferring the vial to a heating block with a forceps. Radiochemical purity (RCP) was evaluated with reverse HPLC for DOTANOC and ITLC for SomaKit. Overall yields, including elution yield and synthesis yield, were calculated corrected for decay in order to compare the labeling performance. Hand radiation exposure was evaluated using APVL-ED3 detector with two probes fixed on the first phalanx of each middle finger.

**Results** Overall yields were respectively 57.0 +/- 11.3 % (*n* = 157) and 64.8 +/- 5.6 % (*n* = 35) respectively for DOTANOC and SomaKit. RCP was always > 98 % for DOTANOC and 96.9 +/- 6.9 % for Somakit include 5 (15 %) non-compliant RCP. To note that 30 to 35 min are required between generator elution and batch release for both process, but preparative step lasts at least 30 min for DOTANOC versus less than 10 min for Somakit. The radiation exposure measurements (extrapolated for a generator at calibration) gave 70 and 132 μSv for the middle finger (left and right hand) and 179 and 152 μSv respectively for DOTANOC and SomaKit.

**Conclusion:** Somakit radiopharmaceutical kit not only allowed to turn the preparation of ^68^Ga-DOTATOC into a straightforward routine process, but it also led to an increase in the overall synthesis yield compared to automated DOTANOC radiolabeling. However, the lack of purification step in Somakit radiolabeling process prevents its injection to patient in case of incomplete complexation of Ga-68. Also, process including purification provides PRC consistently higher for DOTANOC *vs.* Somakit. Shifting from an automated to a manual process in routine also raises the question of radiation safety. This last encourage automation of Somakit labeling which should also improve the reproducibility and allow moving towards full GMP. This work is in progress.

## PP46 Optimization of automated ^68^Ga-PSMA-11 preparation by switch from tubing to GMP synthesizer

### A. Rauscher^1^ and M. Frindel^1^, P. Baumgartner^1^, M. Le Thiec^1^, F. Kraeber-Bodéré^1,2^, C. Rousseau^1,2^

#### ^1^Institut de Cancérologie de l’Ouest – René Gauducheau, Boulevard Jacques Monod 44805 Saint-Herblain (Nantes), France; ^2^Nantes-Angers Cancer Research Center, INSERM U892, CNRS UMR 6299, University of Nantes, Nantes, France

##### **Correspondence:** A. Rauscher; M. Frindel

**Aim:** The value of ^68^Ga-PSMA-directed PET imaging as a diagnostic procedure for recurrent prostate cancer is assessed and more particularly in early biochemical relapse. Synthesis of ^68^Ga-PSMA-11 is relatively straightforward due to HBED-CC chelation allowing significant synthetic yield even at room temperature. Automation of ^68^Ga-PSMA-11 synthesis can nevertheless be optimized to increase overall radiochemical yield i.e. increase of the available activity to inject more patients by synthesis during the shelf life of gallium-68 generator.

**Methods:** PSMA-11 was purchased from IASON and gallium-68 chloride was obtained by elution of GalliaPharm® generator. Current preparation process has been developed on the ModularLab Standard® (Eckert&Zielger AG, Berlin) tubing synthesizer in a procedure similar to that described by Ceci *et al*. Briefly, the process includes an acetone-free cation exchange post-processing purification (Mueller *et al.*), 95°C heating for 5 min in acetate buffer (2 M, pH 4.5) and purification on disposable Sep-Pak C18 Plus® cartridge (Waters SAS France) by elution with 2 mL ethanol/water (1:1). The resulting product is sterilized by filtration and diluted in physiological saline. Then, the process was transferred on the ModularLab PharmTracer® (Eckert&Ziegler AG) cassette-based synthesizer with the C4-Ga68-PSMA cassette and the same steps and reagents as above. Radiochemical purity was evaluated by reversed-phase HPLC.

**Results** Up to now, 62 syntheses were performed on the tubing synthesizer. Mean observed radiochemical yields (RCY) was 86.1 +/- 4.6 % and mean radiochemical purity (RCP) was 99.0 +/- 0.9 %. Among 10 % of the initial activity is retained on the C18 cartridge. With this process, synthesis is completed in 14 min. Validation of the GMP-cassette synthesis is ongoing. First results were 96.8 % RCY and 99.2 % RCP in 17 min process.

**Conclusion:** Optimization of the process was motivated by safety improvement by using GMP cassettes. Transfer from tubing to cassette module lead also to an increase in RCY. This is explained by the opportunity of using a Sep-Pak C18 Light® cartridge with a lower residual ^68^Ga-PSMA-11 retention. In spite of the intrinsic longer time process, the final product activity is increased and should allow to inject more patient per batch.

**References**

1. Ceci F, Uprimny C, Nilica B, [2015], EJNMMI, 42 : 1284-1294

2. Mueller D, Klette I, Baum RP et al., [2012], Bioconjugate Chem., 23: 1712–1717

## PP47 Effective treatment of microscopic cancers with Tb-161

### Ø.A. Rusten^1^, E. Mengshoel Brevik^1^, S. Hassfjell^1^, K. Tkacz-Stachowska^1^, O. Hjelstuen^1^, H. Hjort Tønnesen^2^

#### ^1^Institute for Energy Technology, Kjeller, Norway, ^2^Department of pharmacy, School of pharmacy, University of Oslo

##### **Correspondence:** Ø.A. Rusten

**Aim:** Prostate cancer is one of the most common cancers among men. Prostate specific membrane antigen (PSMA) has proven to be a useful diagnostic and therapeutic agent in radiotherapy. PSMA-617 is currently one of the most interesting molecules that binds specifically to the PSMA-receptor. ^177^Lu and ^161^Tb are beta emitting radionuclides with similar radiochemical properties. In addition, Tb-161 emits a high number of low energetic conversion- and auger-electrons. Therefore, Tb-161 could be a promising radionuclide for treatment of small tumors and metastases.

**Methods:** PSMA-617 was purchased from ABX, Germany, and used without further modifications. The PSMA-617 was radiolabeled with ^177^Lu (ITG, Germany) and ^161^Tb (IFE, Norway). Cell toxicity studies with ^177^Lu-PSMA-617 and ^161^Tb-PSMA-617 were conducted on the cell line LVCaP (ATCC, USA).

**Results** In this study, PSMA-617 was labeled with Lu-177 and Tb-161 using different reaction conditions to optimize radiochemical yield and purity. The formed complexes ^177^Lu-PSMA-617 and ^161^Tb-PSMA-617 were compared with regard to specific activity, stability and shelf-life.

**Conclusion:** ^177^Lu-PSMA-617 and ^161^Tb-PSMA-617 complexes have been obtained in high radiochemical yield and purity. In the next step, we will use an AR-positive hormone responsive prostate cancer cell line to investigate the therapeutic efficacy of ^177^Lu-PSMA-617 and ^161^Tb-PSMA-617 *in vitro.*

## PP48 Development of targeted radiopharmaceuticals with terbium-161 for use in radioimmunotherapy

### K. Eckell Skåre, E. Mengshoel Brevik, S. Hassfjell, K. Tkacz-Stachowska, O.Hjelstuen

#### Institute for Energy Technology, Kjeller, Norway

##### **Correspondence:** K. Eckell Skåre

**Aim:** Breast cancer is the most common cancer in women and causes over 500 000 deaths worldwide every year. Overexpression of HER2 receptor tyrosine kinase occurs in 30 % of metastatic breast cancers and correlates with poor prognosis. Trastuzumab (Herceptin^®^, Roche, United Kingdom) is a recombinant humanized anti-HER2 monoclonal antibody used for treatment of HER2-positive breast cancer. Studies have shown that anti-HER2 radioimmunotherapy (RIT) with ^177^Lu-labelled trastuzumab is effective in treatment of breast cancer *in vitro*. ^161^Tb exhibits similar radiochemical properties to ^177^Lu, but also yields a significant number of short-range Auger/conversion electrons (≤50 keV). In this study, we want to label and compare the therapeutic efficacy of ^177^Lu-DOTA-trastuzumab and ^161^Tb-DOTA-trastuzumab on the HER2-overexpressing cell line SKBR3.

**Methods:** Trastuzumab was conjugated with *p*-SCN-Bz-DOTA (Macrocyclics, USA) to give the DOTA-trastuzumab conjugate, which was radiolabelled with ^177^Lu (ITG, Germany) and ^161^Tb (IFE, Norway) under identical reaction conditions. Immunoreactivity studies with ^177^Lu-DOTA-trastuzumab and ^161^Tb-DOTA-trastuzumab on the SKBR3 cell line (ATCC, USA) are ongoing.

**Results** The average number of *p-*SCN-Bz-DOTA molecules per antibody was determined radiochemically to 5.4 ± 1.1. Radiochemical yield and purity >98 % was achieved for both ^177^Lu and ^161^Tb. Quality control parameters like pH, appearance, radiochemical purity and *in vitro* stability were studied. The radiolabeled preparations were stable up to 120 h within the tested range of specific activities.

**Conclusion:** This study indicates that the used conjugation and radiolabeling procedures give preparations of high stability and high radiochemical purity. Further evaluation of the efficacy of ^161^Tb-DOTA-trastuzumab and ^177^Lu-DOTA-trastuzumab *in vivo* should be pursued.

## PP49 National regulations in Radiopharmacy: Is the present situation generally acceptable?

### M. Darkovska Serafimovska^1^, E. Janevik-Ivanovska^1,3^,T. Balkanov^2^

#### ^1^Goce Delcev University, Faculty of Medical Sciences, Stip, Republic of Macedonia; ^2^University St. Cyril and Methodius, Faculty of Medicine, Skopje, Republic of Macedonia; ^3^University Institute for positron-emission Tomography, Skopje, Republic of Macedonia

##### **Correspondence:** M. Darkovska Serafimovska

**Aim:** The aim of this overview was to evaluate the regulations in Republic of Macedonia dealing with radiopharmaceuticals with emphasis on Marketing authorisation, keeping in mind that the way how radiopharmaceuticals is developed and introduced to the users are completely different from what was typical for conventional pharmaceutical products.

**Specific issues:** The quality of the radiopharmaceuticals is defined in the applicable pharmacopeia monographs of individual radiopharmaceuticals. Regarding the processes of registration of radiopharmaceuticals and production of radiopharmaceuticals according to Good Manufacturing Practises, similar uniformity is not present in all countries. The Good Radiopharmaceutcial Practice Code was defined to comprise the application of the GMP guidelines for Pharmaceuticals with the relevant guidelines for radiation protection during production, distribution and hospital handling of radiopharmaceuticals to ensure the safety and efficacy of the product administered to the patient. Over the years the need for regulation of the radiopharmaceuticals became necessary. The discussions lead to the realisation that radiopharmaceuticals could not be considered as something extraordinary, and that the same quality standards would have to apply for these products as for conventional Pharmaceuticals. The first formal systems for obtaining market authorisation for radiopharmaceuticals were established in the USA, United Kingdom and Denmark. It was obvious that the requirements 653 used for non-radioactive drugs could not be applied directly to the radiopharmaceuticals. Most countries have established national registration procedures to obtain official market authorisation for a radiopharmaceutical. In Republic of Macedonia radiopharmaceuticals are include under the general legislation for medicinal products. Republic of Macedonia is a small country and use of radiopharmaceuticals is small. Therefore the interest in registering these products is small.

Status of radiopharmaceutical regulations in Republic of Macedonia

1. Law on Medicines and Medical Devices (Official gazette No. 106/07,88/2010, 36/11, 53/11, 136/11, 11/12, 147/13, 27/14, 43/14, 88/15) in which radiopharmaceuticals are regulated
2. Rulebook on the contents of the application, the documentation and detailed requirements in respect of the premises, equipment and staff for granting authorisation for production of medicinal products (Official gazette No. 106/07,88/2010, 36/11, 53/11, 136/11, 11/12, 147/13, 27/14, 43/14, 88/15)
3. Rulebook on Good Manufacturing Practices (fully in a compliance with EudraLex Volume 4 Good Manufacturing Practices Annex 1 for Manufacture of Sterile Medicinal Products 01/fully 03/2009, Annex 3 for Manufacture of Radiopharmaceuticals, Annex 11 for Computerized Systems and Annex 15 for Qualification and validation.
4. Law on Ionizing Radiation Protection and Safety (Official gazette No.48/02, 135/07, 53/11, 164/13, 43/14, 149/15)

**Conclusion:** The Law on medicinal products and medical devices of the Republic of Macedonia is completely in compliance with the EU regulative. But, it is obvious that the requirements used for non-radioactive drugs could not be applied directly to the radiopharmaceuticals. Several special characteristics of the radiopharmaceuticals had to be taken into consideration. Keeping in mind that recently in Republic of Macedonia PET centre was established and we have our own production of radiopharmacuticals it is necessary to take some steps forward and make the effort to separate radiopharmaceuticals from general legislation for medicinal products. Most requirements concerning documentation of the safety and efficacy of a non-radioactive drug must be also appled to radiopharmaceuticals. But, it must be recognised that radiopharmaceuticals do not have a measurable pharmacodynamic effect. In the same time radiopharmaceuticals have a changing composition with time due to the radioactive decay of the ultra-short lived radionculides, so sometimes it is impossible to perform quality control.

Studies of chronic toxicity would only be required in special cases. Clinical documentation would normally be more limited than for non-radioactive drugs. Therefore, Republic of Macedonia must to establish national registration procedures to obtain official market authorisation for a radiopharmaceutical, taking bearing in mind that we did not have a drug legislation that did also cover radiopharmaceuticals.

## PP50 Optimization of production of [^11^C]CH_3_I with Methylator II for synthesis and development of ^11^C radiopharmaceuticals

### M. Atanasova^1,2^, M. Chochevska^1,2^, K. Kolevska^1,2^, M. Velickovska^1^, F. Jolevski^1^, E. Janevik-Ivanovska^1,2^

#### ^1^University Institute for positron-emission Tomography, Skopje, Republic of Macedonia; ^2^Faculty of Medical Sciences, University Goce Delčev Stip, Republic of Macedonia

##### **Correspondence:** E. Janevik-Ivanovska

**Aim:** University Institute of Positron Emission Tomography Skopje is equipped with the Methylator II (Comecer Spa. Former Veenstra Instruments BV.), a module designed for the production of high specific activity MethylIodide ([^11^C]CH_3_I) and/or Methyl Triflate ([^11^C]CH_3_OSO_2_CF_3_) and CarbonSynthon I (Comecer Spa.) for production of simple 11C radiopharmaceuticals. The synthesis process starts with the production of [^11^C]CO_2_ in the cyclotron (GE PETtrace 16.5MeV) via the ^14^N(p,α)^11^C nuclear reaction. The produced [^11^C]CO_2_ is delivered into the Methylator, where it first was trapped and subsequently reduced to [^11^C]CH4 and converted thereafter into [^11^C]CH_3_I and/or [^11^C]CH_3_OSO_2_CF_3_. The trapped [^11^C]CO_2_ in the Methanizer was reduced into a [^11^C]CH_4_ with hydrogen on a nickel catalyst (Shinwasorb) at a rather moderate temperature 350 ^0^C. The next step was the purification of the [^11^C]CH_4_ over a Carboxen 1000 column, with the knowledge that the H_2_ will flow about 7 times faster than [^11^C]CH_4_ through carbon packing causing the separation of H_2_ and CH_4_. This is one of the most important steps in the production process which affects directly the equilibrium reaction which forms the [^11^C]CH_3_I and HI, which is formed in the iodine oven by the reaction of H2 and I2 as well

**Methods:** Optimization experiments where performed maximizing the yield of [^11^C]CH_3_I. By changing the time for switching the valve V04 (see diagram) the effectiveness of the purification was influenced. In ‘Active’ state the formed [^11^C]CH_4_ and excess of H_2_ was directed toward waste, but in ‘Inactive’ state in direction of the Iodine Oven. If the time was too short the reduced [^11^C]CH_4_ would not be separated thorougly enough from the H_2_, but when the time was too long the produced [^11^C]CH_4_ would be lost into waste. The first syntheses were performed with V04 active for 25 sec upon release of the [^11^C]CH_4_, after which it was deactivated. Different timings for switching the valve were tested and the different yields were obtained.

**Fig. 1 (abstract PP50).**
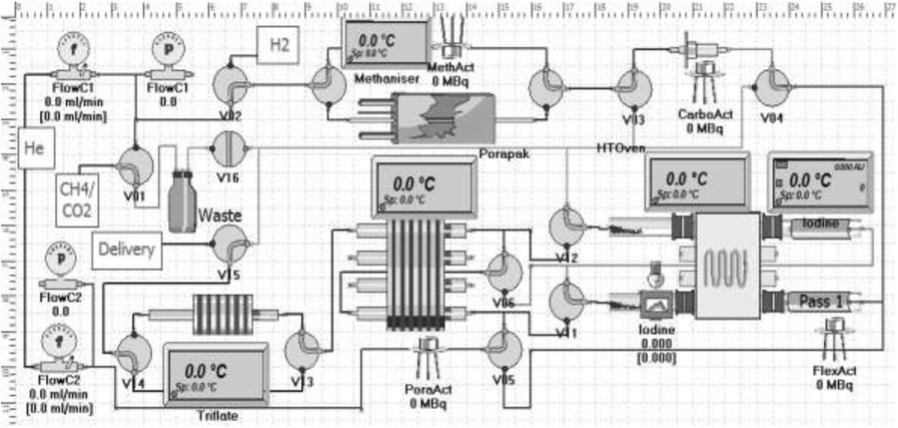
See text for description

**Results** Our result presented in the Table showed that yield of [^11^C] CH_3_I and [^11^C] Choline is purification time depended. By increasing the time of purification (from 20 to 37 seconds) obtained trapped [^11^C] CO_2,_ is more than four time higher and harvested [^11^C] CH_3_I as well. After 37 seconds we obtained 41% of [^11^C] CH_3_I that is directly reflected to the yield of [^11^C] Choline (34.6), fitting with our protocol for synthesis of [^11^C] Choline.

**Table 1 (abstract PP50).** Results of testing

Purification time [s]	Trapped [^11^C]CO_2_ [GBq]	Harvested [^11^C]CH_3_I [GBq]	Yield [^11^C]CH_3_I dc [%]	Yield [^11^C] Choline d.c [%]
20	4.5GBq@17:47	0.8 GBq@17:59 1.2 GBq@17:47	27	22
25	7.2 GBq@1280s	1.25 GBq@2500s 2.5 GBq@1280s	35	25
37	19.564GBq@12:32	6.280GBq@12:39 8.1GBq@12:32	41.4	34.6

**Conclusion:** The module and software give us a big opportunity and flexibility for testing and optimization of the production achieving a better yield, and also the development of new ^11^C radiopharmaceuticals.

## PP51 Formulation and Characterization Studies of Radiolabeled, Active Folate Targeted Theranostic Co-Delivery Liposomes for Non-Small Cell Lung Cancer

### M. Karpuz-Oguz^1,2^, M. Silindir-Gunay^1^, A. Yekta Ozer^1*^

#### Department of Radiopharmacy, Faculty of Pharmacy, Hacettepe University, 06100, Ankara, Turkey; ^2^Department of Radiopharmacy, Faculty of Pharmacy, İzmir Katip Celebi University, 35620, İzmir, Turkey

##### **Correspondence:** A. Yekta Ozer

**Aim:** Cancer is the main cause of the deaths in the worldwide. Among cancer types, lung cancer is the most common one and it is currently the leading cause of cancer death. 80% to 85% of lung cancers compose non-small cell lung cancer (NSCLC) [1]. Combination therapy in cancer has many advantages including reducing the toxic effects exists in monotherapy, minimizing multidrug resistance and ensuring synergistic effect for clinical response in comparison with monotherapy [2]. Hybrid diagnostic imaging modalities like SPECT/CT can provide both metabolic, functional and anatomic information about the diseases. Using specific agents is essential for sufficient functional information, better diagnosis and effective therapy of NSCLC. Passively or actively targeted systems such as liposomes have different interests for either therapy or diagnosis. Recent studies generally depend on the development of new delivery systems, theranostics, in which diagnosis can be managed together with cancer therapy by evaluating therapeutic effect.

**Methods:** Theranostic liposomes were formulated by polyethylene glycole (PEG)ylated, nanosized, either passive or actively (folate conjugated) targeted, ^99m^Tc labeled for SPECT/CT imaging and vinorelbine and paclitaxel encapsulated for NSCLC therapy. Their characterization studies were conducted.

**Results** Both passive and active targeted liposomes showed proper characterization with about 10% encapsulation efficiency of vinorelbine and 60% encapsulation efficiency of paclitaxel; around 100 nm particle size of passive targeted liposomes and around 120 nm particle size of active targeted formulations. All formulations designated a zeta potential around -10 - -13 mV and phospholipid efficiency of 85%. All formulations designated a stable radiolabeling with 99mTc.

**Conclusion:** Promising characterization profiles were obtained with both nanosized, PEGylated passive targeted and active targeted co-delivery theranostic liposomes for both diagnosis and therapy of NSCLC. Our studies are continuing.

**Funding**: This study was supported by the grant of H.U. BAB, Project No: 12852. Paclitaxel is obtained from Bristol-Meyers Squibb as a generous gift for our non-clinical research.

**References**

1. Torre LA, Bray F, Siegel RL, Ferlay J, Lortet-Tieulent J, Jemal A, 2012, Cancer J Clin 65: 87-108.

2. Wu M, Sirota M, Butte AJ, Chen B,2015, Pac Symp Biocomput: 68-79.

## PP52 HPLC method for analysis of DOTA-TOC

### M. Radzik, A. Jaroń, M. Maurin, P. Garnuszek

#### National Centre for Nuclear Research Radioisotope Centre POLATOM, Otwock, Poland

##### **Correspondence:** P. Garnuszek

**Aim:** Main impurities in the ^90^Y-DOTA-TOC labelling process, apart from the “free” yttrium-90, are DOTA-TOC complexes with other metals that could be present in the solution. The most common method for determination of radiochemical purity (RCP) of labeled peptides is RP-HPLC. However, the standard gradient HPLC methods may not allow determination of the DOTA-TOC complexes with “cold” metals. The information about presence of cold metal complexes might be very important in particular for investigation of reasons of radiolabeling failure and poor RCP. The aim of the study was to develop a new HPLC method that would be suitable for determination of RCP as well as for identification and determination of cold metal complexes that could be present in ^90^Y-DOTA-TOC.

**Methods:** The new isocratic RP-HPLC method (Phenomenex Kinetex 150x4.6mm column and isocratic flow of 80% 0.1% TFA in water and 20% of 0.1% TFA in acetonitrile, 1mL/min) has been developed and validated. Accuracy, repeatability, precision, linearity and range of the method was checked. DOTA-TOC complexes with “hot” yttrium-90 and lutetium-177 and “cold” complexes with indium, lutetium, yttrium, gallium, zinc, copper, iron and lead were evaluated. The formation of complexes has been confirmed by LC-MS.

**Results** A very good chromatographic separation of the individual DOTA-TOC metal complexes has been achieved. Each of the metal complexes can be easily identified by their characteristic retention times and could be quantitated using estimated molar absorption coefficients. The method enabled quantitative determination of unlabeled peptide in the radioactive preparation in the range of linearity from 10 mcg to 100 mcg of DOTA-TOC (R2 = 0.999).

**Conclusion:** The developed isocratic HPLC method is suitable for RCP determination of radiolabeled DOTA-TOC. In single HPLC run both the RCP and the contribution of DOTA-TOC complexes with cold metals can be assess, thus reflecting the specific activity of the radiolabeled peptide. Validation confirmed that the method is accurate, repeatable and precise.

## PP53 Radiochemical purity determination of ^68^Ga-labelled radiopharmaceuticals. Are the TLC and HPLC results complementary?

### P. Garnuszek, M. Maurin, M. Radzik

#### National Centre for Nuclear Research Radioisotope Centre POLATOM, Otwock, Poland

##### **Correspondence:** P. Garnuszek

**Aim:** European Pharmacopoeia monograph for Gallium (^68^Ga) edotreotide [1] recommends HPLC and TLC as complementary methods for radiochemical purity (RCP) determination. In TLC [^68^Ga]gallium in colloidal form is determined while HPLC separates the free [^68^Ga]gallium(III) ion and [^68^Ga]gallium edotreotide. Mobile phases used in these methods have different pH values. Being aware that gallium speciation in aqueous solution depends on pH and temperature [2], the intrinsic questions arise: (1) what ^68^Ga species are realy determined, (2) are the TLC results matching the HPLC results? Herein we are answering these questions based on our experience with ^68^Ga-labelled radiopharmaceutical preparations.

**Methods:** TLC and HPLC methods for RCP determination of ^68^Ga-DOTA-TOC and ^68^Ga-DOTA-TATE were based on Ph.Eur. [1], for ^68^Ga-PSMA on the draft monograph [3]. Other TLC mobile phases were also used: 1M ammonium acetate, 1M sodium acetate/methanol, 5M sodium acetate/methanol, citrate buffer pH 5.5. Reference solutions of ^68^Ga were prepared [2], in HCl (prepared by addition of 1.5mL of H_2_O to 1mL of ^68^Ga eluate in 0.1M HCl) and in sodium acetate (24mg/ml). RCP in all samples was assessed by HPLC and TLC followed by calculation of ^68^Ga recovery.

**Results** In HPLC the differences in recovery of ^68^Ga(III) and ^68^Ga in colloidal form, 107%±1.7% and 106.2%±1.3%, respectively, were not statistically significant. In ^68^Ga-PSMA11 (*n* = 12) both HPLC and TLC revealed very similar results of free ^68^Ga: 1.64%±0.59% (HPLC) and 1.55%±0.65% (TLC). Similar observations were made in ^68^Ga-DOTA-TOC and ^68^Ga-DOTA-TATE.

**Conclusion:** Depending on pH of mobile phase, the non-bound ^68^Ga may exists in various ionic forms. At pH around 2, HPLC analysis of ^68^Ga reference sample revealed significant transition to Ga^3+^ or Ga(OH)^2+^ cations, allowing quantitation of free ^68^Ga with mobile phase containing 0.1%TFA. TLC results varie depending on mobile phase composition. Hence, we suggest that TLC is not to be used as complementary to HPLC but as alternative method for free ^68^Ga determination, even though when using 1M sodium acetate/methanol (50/50) the results of free ^68^Ga were consistent with HPLC. It should be stressed that methods for determination of non-bound ^68^Ga in radiopharmaceutical preparations do not reflect gallium speciation in aqueous solutions.

**Funding**: This work was partly financed by grant No. POIR/01.02.00-00-0041/15 from the National Centre for Research and Development

**References**

1. Gallium (^68^Ga) edotreotide injection. Ph.Eur. 01/2013:2482.

2. Benezeth, P., et al., [1997] Geochim. Cosmochim. Acta 61(7) : 1345-1357

3. Gallium (68Ga) PSMA-11 Injection. Ph.Eur. draft monograph 3044. PA/PH/Exp. 14/T (16) 45 ANP. Pharmeuropa 29.4

